# ^1^H nuclear magnetic resonance‐based metabolomics study of serum and pectoralis major for different commercial chicken breeds

**DOI:** 10.1002/fsn3.2968

**Published:** 2023-04-07

**Authors:** Chengkeng Tan, Jinap Selamat, Nuzul Noorahya Jambari, Rashidah Sukor, Suganya Murugesu, Azira Muhamad, Alfi Khatib

**Affiliations:** ^1^ Laboratory of Food Safety and Food Integrity (FOSFI), Institute of Tropical Agriculture and Food Security Universiti Putra Malaysia (UPM) Serdang Malaysia; ^2^ National Public Health Laboratory Ministry of Health Malaysia Sungai Buloh Malaysia; ^3^ Department of Food Science, Faculty of Food Science and Technology Universiti Putra Malaysia (UPM) Serdang Malaysia; ^4^ Malaysia Genome Institute National Institutes of Biotechnology Malaysia (NIBM) Kajang Malaysia; ^5^ Department of Pharmaceutical Chemistry, Faculty of Pharmacy International Islamic University Malaysia Kuantan Malaysia; ^6^ Faculty of Pharmacy Airlangga University Surabaya Indonesia

**Keywords:** chicken, metabolomics, NMR, pectoralis major, serum

## Abstract

This study aimed to characterize the metabolic composition of four types of commercially available chicken breeds [village chicken, colored broiler (Hubbard), broiler (Cobb), and spent layers (Dekalb)] by ^1^H NMR coupling and discriminate them using multivariate analysis. Five chickens were collected for each chicken breed based on the marketing age from the respective commercial farms. The orthogonal partial least squares discriminant analysis (OPLS‐DA) results showed an obvious separation of local village chickens from the other breeds based on the metabolites present in their serum and meat (pectoralis major). The cumulative values of *Q* ^2^, *R* ^2^ *X*, and *R* ^2^ *Y* of the OPLS‐DA model for chicken serum were 0.722, 0.877, and 0.841. For the pectoralis major muscle, the cumulative values of *Q* ^2^, *R* ^2^ *X*, and *R* ^2^ *Y* of the OPLS‐DA model were reported as 0.684, 0.781, and 0.786, respectively. The quality of both OPLS‐DA models was accepted by the cumulative values of *Q* ^2^ ≥ 0.5 and *R* ^2^ ≥ 0.65. The ^1^H NMR result with multivariate analysis has successfully distinguished local village chicken from the other three commercial chicken breeds based on serum and pectoralis major muscle. Nonetheless, colored broiler (Hubbard) was not distinguished from broiler (Cobb) and spent layers (Dekalb) in serum and pectoralis major, respectively. The OPLS‐DA assessment in this study identified 19 and 15 potential metabolites for discriminating different chicken breeds in serum and pectoralis major muscle, respectively. Some of the prominent metabolites identified include amino acids (betaine, glycine, glutamine, guanidoacetate, phenylalanine, and valine), nucleotides (IMP and NAD+), organic acids (lactate, malate, and succinate), peptide (anserine), and sugar alcohol (myo‐inositol).

## Graphical abstract

This study aimed to characterize the metabolic composition in four types of commercially available chicken breeds (village chicken, colored broiler [Hubbard], broiler [Cobb], and spent layers [Dekalb]) by 1H NMR coupled and discriminate them using multivariate analysis. The orthogonal partial least squares discriminant analysis (OPLS‐DA) results showed an obvious separation of local village chicken from the other breeds based on the metabolites present in their serum and meat (pectoralis major). The OPLS‐DA assessment identified 19 and 15 potential metabolites for discriminating different chicken breeds in serum and pectoralis major muscle, respectively.

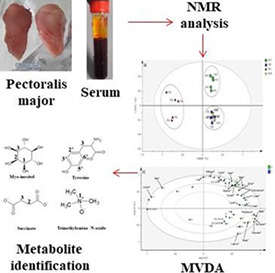

## 1 INTRODUCTION

Chicken is perceived as the main source of protein for human consumption worldwide. Generally, broiler chicken is the major breed raised for meat production in the poultry meat sector. Nonetheless, the demand for local village chicken is also continually increasing and is famous for its health benefits, better meat quality, and palatability (Mohd Shahmi Hakimi et al., 2019). For instance, village chicken is a slow‐growing chicken that reaches an average market size of 1–1.5 kg at 14–20 weeks of marketing age (Ramlah, 1996), with a price that is approximately two to four times higher than fast‐growing chicken (Feng et al., 2018). For instance, food fraud involving the sale of cheaper chicken breeds and claimed as village chicken, which sells at a higher price, might occur in markets driven by the increasing demand for village chicken (Azhar, 2019; Hakim, 2019). Furthermore, such counterfeiting risks and food fraud can occur in the poultry meat sector by manipulating the marketing age of cheaper chicken breeds due to the similarities in size and physical appearance for economic gain (Fontanesi, 2017). Therefore, a feasible solution is urgently required to warrant fair trading and protect the consumers' rights.

The metabolomics study focuses on small metabolites (<1500 Da) in living organisms (Miggiels et al., 2019). The information gained, such as metabolic responses to the changes in genetic and environmental conditions, could reliably characterize the physiological state of the living organisms (Markley et al., 2017). Mass spectrometry, including liquid and gas chromatography and nuclear magnetic resonance (NMR), has been employed extensively in metabolomics studies. NMR is a non‐biased and non‐destructive analytical platform for metabolite identification and elucidation in both targeted and untargeted analyses (Bisht et al., 2021). Although the sensitivity of the NMR analytical platform is not similar to mass spectrometry, NMR is known as a reliable method capable of characterizing small molecules with minimal sample preparation and high throughput. In recent years, metabolomic studies using NMR have been employed in meat authentication, including beef from different geographical origins (Jung et al., 2010), beef and horse meat (Jakes et al., 2015), duck breeds (Wang et al., 2016), and different species of chicken, beef, chevon, and donkey meat (Mukhtar et al., 2021).

In the present study, four commercial chicken breeds, namely local village chicken, broiler chicken (Cobb), spent layers (Dekalb), and colored broiler (Hubbard), were directly purchased from the corresponding commercial farms. All chicken breeds were raised at commercial farms under similar standard conditions. To the best of the author's knowledge, official biomarkers to discriminate the local village chicken from other commercial breeds through the NMR‐based metabolomics approach have not yet been reported. Thus, the present study aimed to discriminate four different types of commercial chicken breeds with an untargeted NMR‐based metabolomics approach and further investigate the metabolite profiles of serum and pectoralis major muscle applied for chicken breeds discrimination.

## 2 MATERIALS AND METHODS

### 2.1 Reagents

Deuterated water (D_2_O) was procured from Sigma‐Aldrich (St. Louis, MO, USA). The analytical grade of chemicals and organic solvents procured from Merck (Frankfurter Strasse, Darmstadt, Germany) is methanol, sodium 3‐(trimethylsilyl)‐propionate‐2, 2, 3, 3‐d_4_ (TSP), sodium deuteroxide solution (NaOD), and potassium dihydrogen phosphate (KH_2_PO_4_).

### 2.2 Sample collection

Four different chicken breeds (female) were directly procured from the local commercial farms according to their respective marketing age, including local village chicken, broiler chicken (Cobb), spent layers (Dekalb), and colored broiler (Hubbard). Five chickens were collected for each chicken breed from the commercial farms. The village chickens obtained in the present study are from crossbreeding of red jungle fowl with other breeds from European countries through a random and unplanned mating system (Azahan, 1994). The details of marketing age, live weights, rearing conditions, and stocking densities of the birds are summarized in Table 1. The four chicken breeds were fed ad libitum with the same brand (Gold Coin) of corn and soybean‐based commercial feeds and water and reared under standard commercial conditions at respective commercial farms. All birds were sacrificed in the slaughterhouse at the Faculty of Agriculture, UPM, by the authorized personnel. Before slaughtering, all chickens were fasted for 8 h. The collected pectoralis major (PM) muscle samples were immediately subjected to snap‐frozen using liquid nitrogen and stored at −80°C during the sampling. Meanwhile, the chicken blood samples were obtained during bleeding and kept at room temperature for approximately 15 min. Then, all coagulated blood samples were further centrifuged (10 min × 4000 *g*) for separation. After centrifugation, the chicken serum was filled into individual microcentrifuge tubes accordingly for storage at −80°C (Beauclercq et al., 2016). All the experimental animals and procedures conducted in this study were approved by the Institutional Animal Care and Use Committee (IACUC), Universiti Putra Malaysia (approval number: UPM/IACUC/AUP‐R022/2019).

**TABLE 1:** Four chicken breeds, marketing age, live weight, rearing condition, and farm locations

Chicken breeds	Marketing age (weeks)	Live weight (g)	Rearing condition	Stocking density (birds per m^2^)	Farm location
Authentic village chicken	14	1010.5 ± 47.6 g	Opened house floor system	8	Ladang Pahang Tua, Pahang
Colored broiler (Hubbard)	10	2055.5 ± 66.4 g	Opened house floor system	8	Ladang Bukit Mertajam, Penang
Broiler (Cobb)	6	2487.5 ± 53.5 g	Closed house floor system	8	Ladang Permatang Tinggi, Penang
Spent layers (Dekalb)	72	1847.9 ± 58.7 g	Closed house cages system	11	Ladang Butterworth, Penang

### 2.3 Preparation of chicken serum samples for NMR analysis

Chicken serum samples were prepared as described by Maulidiani et al. (2016), with some modifications. All chicken serum samples were thawed at room temperature before analysis. About 400 μl of phosphate buffer (0.01 M, pH 7.4) with 0.2% of TSP in 99% D_2_O was added to 200 μl of serum samples. All samples were mixed well and centrifuged (4000 *g*, 15 min, 4°C). About 500 μl of the supernatant was transferred carefully into NMR tubes for NMR analysis.

### 2.4 Preparation of pectoralis major muscle samples for NMR analysis

PM muscle samples were prepared as described by Yang et al., 2019, with some modifications. About 400 mg of PM muscle was homogenized with 600 μl mixed solution of methanol and ultrapure water (2:1, v/v) for 2 min and sonicated in an ice water bath for another 2 min. The samples were further subjected to centrifugation (12,000 rpm, 4°C, 10 min). After centrifugation, the supernatant was collected in a new microcentrifuge tube. These steps were repeated three times for all samples. Next, the collected supernatant was combined in the same microcentrifuge tube and dried under a nitrogen gas flow. After dryness, the sample was reconstituted with 120 μl of 99% D_2_O (containing 0.005% TSP) and 480 μl of 99% D_2_O. After centrifugation (12,000 rpm, 4°C) for 10 min, 500 μl of supernatant was inserted carefully into NMR tubes for NMR analysis. In addition, all samples were kept in chilled condition (2–5°C) before NMR analysis.

### 2.5 NMR analysis

^1^H NMR spectra of chicken serum and PM muscle samples were measured using a Bruker Avance DRX 700 MHz NMR spectrometer at 298 K, coupled with a TXI probe. Water pre‐saturation and the Carr–Purcell–Meiboom–Gill (CPMG) pulse sequence were employed for suppressing protein resonances and water signals. All the samples were analyzed with three technical replications. The acquisition setting is as follows; the spectrum width was 8403 Hz, the relaxation delay was 4 s, and the number of acquisitions was 128 scans. For NMR signal assignments in both matrices, two‐dimensional (2D) NMR spectra, including ^1^H J‐resolved spectroscopy (J‐Res) and ^1^H–^13^C heteronuclear multiple‐bond correlation spectra (HMBC), were conducted on selected chicken serum and PM muscle samples.

### 2.6 NMR data pre‐processing and analysis

Data pre‐processing of NMR data was performed according to Razali et al. (2018). In short, manual phasing and baseline correction were performed to all ^1^H NMR spectra for chicken serum and PM muscle, respectively, by employing Chenomx NMR Suite 8.2 (Chenomx Inc). All signals were referenced against the TSP, an internal reference with a known concentration, and further analyzed against Chenomx Library. The bucketing procedure was conducted for the spectra, ranging from 10.0 to 0.5 ppm with a bucket width of 0.04 ppm. The water region (4.68–4.88 ppm) and methanol region (3.24–3.33 ppm) were excluded from bucketing. A total of 233 bins of X‐variables were obtained for each spectrum. The pre‐processed NMR dataset was transformed into Excel sheet form and analyzed by multivariate analysis (PCA and OPLS‐DA) using SIMCA P^+^15.0 software (version 15.0; Umetrics). PCA models displayed as score plots were evaluated with the summary of the fit test. Furthermore, OPLS‐DA models were generated with a 7‐fold cross‐validation method and displayed as score and loading plots. OPLS‐DA models were validated by cumulative values of *R* ^2^ and *Q* ^2^ for the models' interpretation and predictability ability, respectively. Furthermore, permutation tests were conducted on the OPLS‐DA models for robustness evaluation (Wheelock & Wheelock, 2013). The characteristic metabolites were identified by checking the chemical shift, multiplicity, and coupling constant values (by 2D J‐Res) of each proton NMR signal, as well as the proton and carbon coupling (by HMBC spectra). This information was compared to the database supplied by Chenomx library, Human Metabolome Database (HMDB), ChemSpider, and PubChem.

### 2.7 Semi‐quantitation of chicken serum and pectoralis major

Semi‐quantitation was performed on all metabolites identified in chicken serum and PM muscle. Additionally, two‐sample T‐tests were conducted using Minitab 17 to determine the significant difference in the relative intensity mean values of ^1^H resonances between two chicken breeds from different clustering for metabolites identified in chicken serum and PM muscle, respectively.

## 3 RESULTS

The four chicken breeds observed in this study include local village chicken, broiler chicken (Cobb), spent layers (Dekalb), and colored broiler (Hubbard); hereafter referred to as AVC, CBC, DSL, and HCB, respectively.

### 3.1 ^1^H NMR spectra of serum and pectoralis major samples

Representative 700 MHz ^1^H NMR spectra data of chicken serum and PM muscle in the present study are displayed in Figure S1. A total of 30 and 29 metabolites for chicken serum and PM muscle have been identified, respectively. The numerical keys for annotated metabolites for chicken serum and PM muscle are displayed in Tables 2 and 3, respectively. Metabolites identified in both chicken serum and PM muscle consisted of an abundance of metabolites including amino acids (betaine, creatine, glutamine, glycine, leucine, N,N‐dimethylglycine, and phenylalanine), nucleotides (inosine monophosphate [IMP] and NAD+), organic acids (3‐hydroxybutyrate, fumarate, and lactate), peptide (anserine), sugar (glucose and mannose), sugar alcohol (myo‐inositol), and others. The chemical structures of identified metabolites for chicken serum and PM muscle are presented in Figures S2 and S3. Additionally, 2D J‐resolved NMR spectra were obtained from topspins for all metabolites identified in chicken serum and PM muscle, as reported in Figures S4 and S5.

**TABLE 2:** ^1^H NMR (**δ**) chemical shift, proton number, multiplicity, and coupling constants (Hz) of tentative metabolites identified in chicken serum

Key signal	Metabolites	Chemical shift, ppm (proton number; multiplicity^a^; and coupling constant)^b^
1	3‐Hydroxybutyrate	1.18 (H‐4; d; 6.1 Hz), 2.30 (H‐2; dd; 14.5, 6.4 Hz), 2.50 (H‐2; dd; 14.5, 6.3 Hz)
2	Acetone	2.22 (H‐1,3; s)
3	Alanine	1.46 (H‐3; d; 7.7 Hz), 3.78 (H‐2; q; 4.8 Hz)
4	Betaine	3.30 (H‐3; s), 3.90 (H‐1; s)
5	Citrate	2.54 (H‐1; d; 15.2 Hz), 2.70 (H‐3; d; 15.6 Hz)
6	Creatine	3.02 (H‐2′; s), 3.94 (H‐2; s)
7	Creatinine	3.06 (H‐1; s)
8	Formate	8.46 (H‐1; s)
9	Fumarate	6.50 (H‐2,3; s)
10	Glutamine	2.14 (H‐3; m), 2.46 (H‐4; m)
11	Glycine	3.58 (H‐2; s)
12	Homoserine	2.10 (H‐3; m), 3.74 (H‐2; m)
13	Hypoxanthine	8.18 (H‐4; s)
14	Lactate	1.38 (H‐3; d; 6.8 Hz), 4.14 (H‐2; q; 7.0 Hz)
15	Leucine	0.94 (H‐5; d; 6.5 Hz), 1.70 (H‐3; m)
16	Lysine	1.74 (H‐5; m), 1.86 (H‐3; m)
17	Malate	2.42 (H‐3; dd; 14, 7.4 Hz), 2.66 (H‐3; dd; 14.1, 7.2 Hz)
18	Mannose	5.10 (H‐2,3,4,5,6; s)
19	Methionine	2.62 (H‐4; t; 7.6 Hz)
20	3‐methylhistidine	6.82 (H‐5′; s)
21	Myo‐inositol	3.54 (H‐1,3; dd; 9.5, 2.6 Hz), 4.06 (H‐2; t; 9.4 Hz)
22	O‐phosphocholine	3.18 (H‐1; s)
23	Phenylalanine	3.22 (H‐3; dd; 13.9, 7.5 Hz), 7.30 (H‐2′,6′; t; 8.4 Hz), 7.42 (H‐3′,5′; t; 8.4 Hz)
24	Succinate	2.38 (H‐2,3; s)
25	Trimethylamine N‐oxide	3.26 (H‐1; s)
26	Tyrosine	3.10 (H‐3; dd; 14.1, 9.4 Hz), 6.90 (H‐3′,5′; d; 8.5 Hz), 7.18 (H‐2′,6′; d; 8.4 Hz)
27	Valine	0.98 (H‐4; d; 6.9 Hz), 2.26 (H‐3; m)
28	Xanthine	7.86 (H‐8; s)
29	α‐Glucose	5.22 (H‐5; d; 4.2 Hz)
30	β‐Glucose	4.66 (H‐5; d; 4.8 Hz)

^a^ Multiplicity of the peak: s, singlet; d, doublet; t, triplet; q, quartet; m, multiplet.

^b^ Chemical shift, multiplicity, and coupling constant are based on J‐resolved spectra as shown in Figure S4.

**TABLE 3:** ^1^H NMR (**δ**) chemical shift, proton number, multiplicity, and coupling constants (Hz) of tentative metabolites identified in pectoralis major muscle

Key signal	Metabolites	Chemical shift, ppm (proton number; multiplicity^a^; and coupling constant)^b^
1	3‐Hydroxybutyrate	1.18 (H‐4; d; 6.3 Hz), 2.30 (H‐2; dd; 14.4, 6.2 Hz), 2.50 (H‐2; dd; 14.5, 6.9 Hz)
2	Acetate	1.94 (H‐2; s)
3	Alanine	1.46 (H‐3; d; 7.4 Hz)
4	Anserine	3.78 (H‐3′; s), 8.22 (H‐1; s)
5	Betaine	3.30 (H‐3; s), 3.90 (H‐1; s)
6	Creatine	3.02 (H‐2′; s), 3.94 (H‐2; s)
7	Formate	8.46 (H‐1; s)
8	Fumarate	6.50 (H‐2,3; s)
9	Glutamate	2.06 (H‐3; m), 2.34 (H‐4; m)
10	Glutamine	2.14 (H‐3; m), 2.46 (H‐4; m)
11	Glycine	3.58 (H‐2; s)
12	Guanidoacetate	3.82 (H‐2; s)
13	Hypoxanthine	8.18 (H‐4; s)
14	IMP	4.02 (H‐1; m), 4.46 (H‐3; dd; 4.8, 4.3 Hz), 6.14 (H‐5; d; 5.9 Hz)
15	Lactate	1.38 (H‐3; d; 6.9 Hz), 4.14 (H‐2; q; 6.9 Hz)
16	Leucine	0.94 (H‐5; d; 6.9 Hz), 1.70 (H‐3; m)
17	Mannose	5.10 (H‐2,3,4,5,6; s)
18	Myo‐inositol	3.54 (H‐1,3; dd; 9.6, 2.4 Hz), 3.62 (H‐4,6; t; 9.8 Hz)
19	N,N‐dimethylglycine	2.94 (H‐3; s), 3.70 (H‐2; s)
20	NAD+	6.02 (H‐5; d; 5.9 Hz), 8.14 (H‐5′; s), 8.38 (H‐3′; s), 8.82 (H‐4′; d; 6.9 Hz)
21	Niacinamide	7.58 (H‐4′; m), 8.70 (H‐5′; s), 8.90(H‐1; s)
22	Phenylalanine	7.30 (H‐4′; d; 9.3 Hz), 7.34 (H‐2′,6′; t; 7.5 Hz), 7.42 (H‐3′,5′; t; 8.2 Hz)
23	Taurine	3.22 (H‐2; t; 7.0 Hz), 3.42 (H‐1; t; 7.2 Hz)
24	Trimethylamine	2.86 (H‐1; s)
25	Uracil	5.78 (H‐3; d; 7.8 Hz), 7.54 (H‐4; d; 7.5 Hz)
26	Valine	0.98 (H‐4; d; 6.7 Hz), 2.26 (H‐3; m)
27	α‐glucose	5.22 (H‐2; d; 4.1 Hz)
28	β‐alanine	2.54 (H‐2; t; 6.5 Hz), 3.18 (H‐3; t; 6.8 Hz)
29	β‐glucose	4.66 (H‐2; d; 5.3 Hz)

^a^ Multiplicity of the peak: s, singlet; d, doublet; t, triplet; q, quartet; m, multiplet.

^b^ Chemical shift, multiplicity, and coupling constant are based on J‐resolved spectra as shown in Figure S5.

### 3.2 Multivariate analysis

**FIGURE 1:**
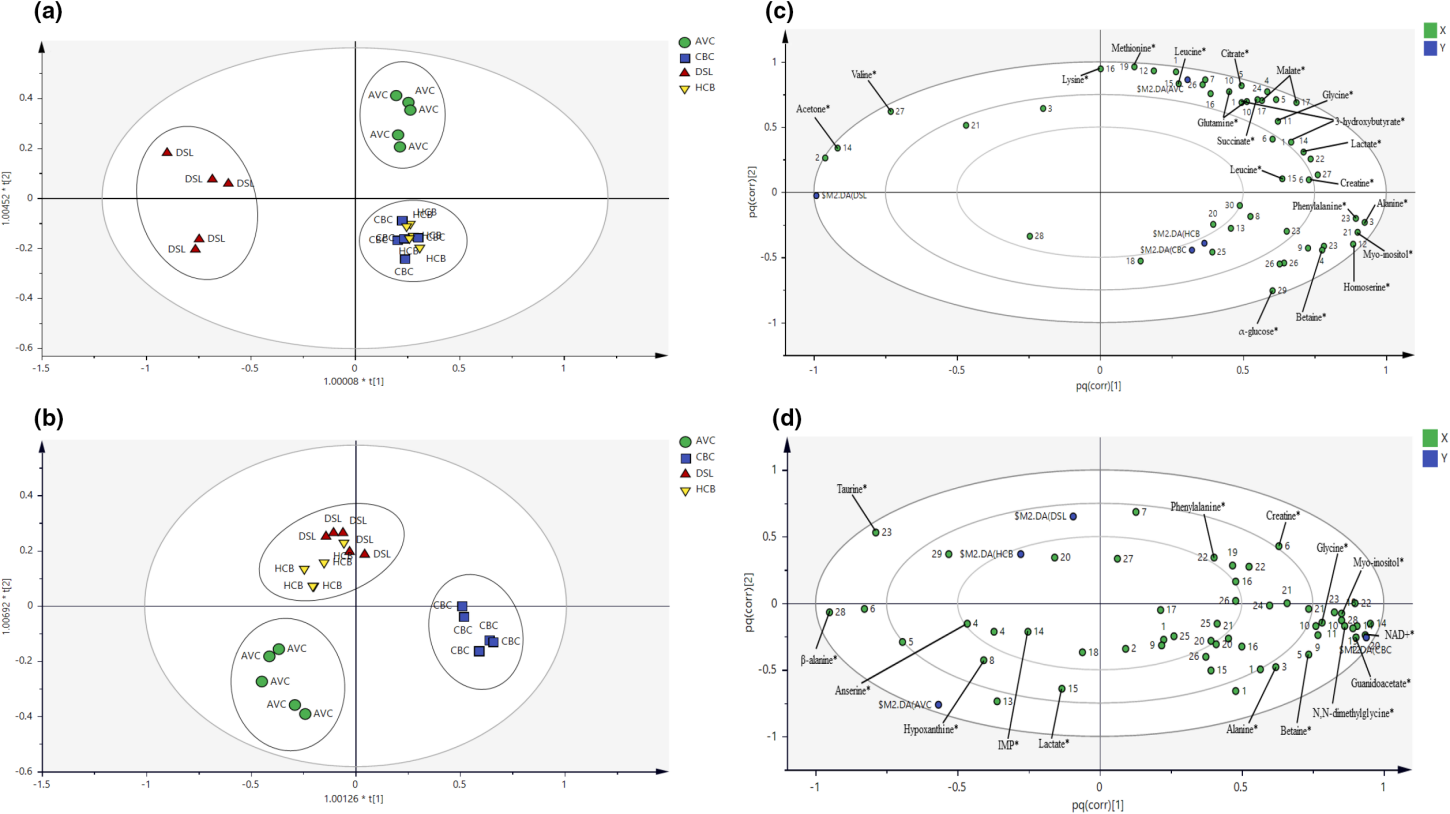
OPLS‐DA score and loading plots for serum and PM of four chicken breeds. (a,b) OPLS‐DA score plots showing the group clustering in serum and PM, respectively. (c,d) OPLS‐DA loading plots showing the metabolites correlated with the clusters in serum and PM, respectively. † Indicates that the metabolites with VIP values >1, and jackknife error bar do not cross zero, as shown in respective loading column plots

Multivariate analysis through principal component analysis (PCA) and orthogonal partial least squares discriminant analysis (OPLS‐DA) was conducted in this study. PCA provides an overview of the distribution of metabolic profiles and differences between four chicken breeds in chicken serum and PM muscle, respectively. The PCA models' validity was evaluated by the summary‐of‐fit test, with a difference between *R* ^2^ *X* and *Q* ^2^ values not larger than 0.3 (Eriksson et al., 2006). In the PCA score plots as displayed in Figure S6, four chicken breeds were discriminated into three clusters for chicken serum and PM muscle. Furthermore, PCA score plots for both matrices showed a clear separation of AVC from other chicken breeds. Besides that, OPLS‐DA was further conducted with the ^1^H NMR spectral data of chicken serum and PM muscle for chicken breed discrimination and putative metabolites selection. In addition, the chicken serum and PM muscle samples were classified into three distinct clusters with 100% correct classification (Table 4) with the chicken breeds. The number of orthogonal components for serum and PM muscle is three and two, respectively. The OPLS‐DA score and loading plots for both matrices are presented in Figure 1. Figure 1a,b show that four chicken breeds were grouped into three distinct clusters for chicken serum and PM muscle, respectively. Also, it is worth noting that similar clusters of four chicken breeds were observed in the PCA score plots. An obvious separation was observed for AVC from other chicken breeds for both matrices in the OPLS‐DA models. For chicken serum, HCB and CBC were mixed in the same cluster located at the lower right of the plot and could not be discriminated (Figure 1a). Furthermore, AVC and DSL were separated into two distinct clusters located at the plot's upper right and left sides. Meanwhile, for PM muscle, HCB and DSL were mixed in the same cluster located at the upper left of the plot and could not be discriminated (Figure 1b). Besides that, AVC and CBC were separated into two distinct clusters located at the lower left and lower right quadrants of the plot, respectively.

**TABLE 4:** The misclassification table for OPLS‐DA models of chicken serum and PM muscles

Chicken breed	% Correct	Classes of chicken serum (OPLS‐DA)			Classes of PM muscles (OPLS‐DA)		
		AVC	CBC & HCB	DSL	AVC	CBC	HCB & DSL
AVC	100	5	0	0	5	0	0
HCB	100	0	5	0	0	0	5
CBC	100	0	5	0	0	5	0
DSL	100	0	0	5	0	0	5
Total average	100						

This study evaluated the quality of OPLS‐DA models based on the cumulative (cum) *Q* ^2^ and *R* ^2^ values. Generally, the model's quality acceptance is indicated by *Q* ^2^ ≥ 0.5 and *R* ^2^ ≥ 0.65 (Eriksson et al., 2006; Williams & Norris, 1987). For chicken serum, the values of *Q* ^2^ (cum), *R* ^2^ *X* (cum), and *R* ^2^ *Y* (cum) were reported as 0.722, 0.877, and 0.841, respectively. Meanwhile, the values of *Q* ^2^ (cum), *R* ^2^ *X* (cum), and *R* ^2^ *Y* (cum) for PM muscle were reported as 0.684, 0.781, and 0.786, respectively. Additionally, OPLS‐DA models were further validated with permutation tests (Figures S7 and S8). The *Q* ^2^ and *R* ^2^ intersect values at the y‐axis of the plot were accessed and validated, as summarized in Table S1. The intersect values of *Q* ^2^ *Y* and *R* ^2^ *Y* do not exceed −0.05 and 0.4, respectively, indicating the validity of the models (Eriksson et al., 2006; Wheelock & Wheelock, 2013).

### 3.3 Characterization of discriminating metabolites

**FIGURE 2:**
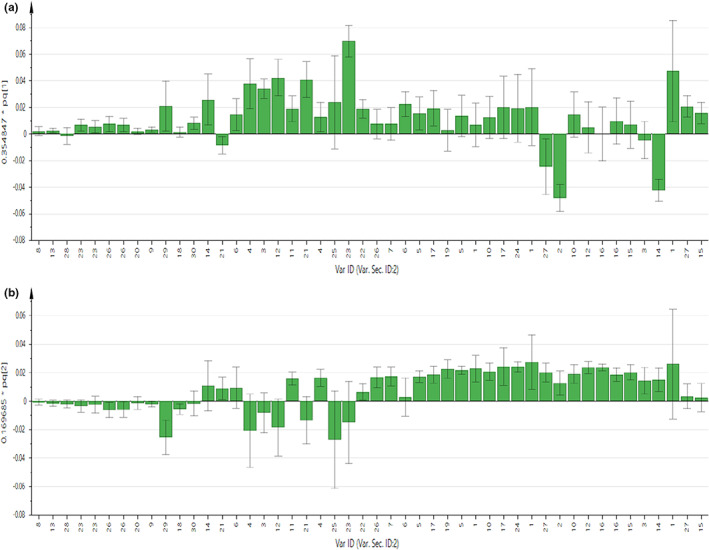
(a,b) OPLS‐DA loading column plots show the contribution of metabolites in the chicken serum for breeds clustering based on PC1 and 2 in score and loading plots, respectively. Key signals: 1. 3‐hydroxybutyrate, 2. Acetone, 3. Alanine, 4. Betaine, 5. Citrate, 6. Creatine, 7. Creatinine, 8. Formate, 9. Fumarate, 10. Glutamine, 11. Glycine, 12. Homoserine, 13. Hypoxanthine, 14. Lactate, 15. Leucine, 16. Lysine, 17. Malate, 18. Mannose, 19. Methionine, 20. 3‐methylhistidine, 21. Myo‐inositol, 22. O‐phosphocholine, 23. Phenylalanine, 24. Succinate, 25. Trimethylamine N‐oxide, 26. Tyrosine, 27. Valine, 28. Xanthine, 29. α‐glucose, and 30. β‐glucose

**FIGURE 3:**
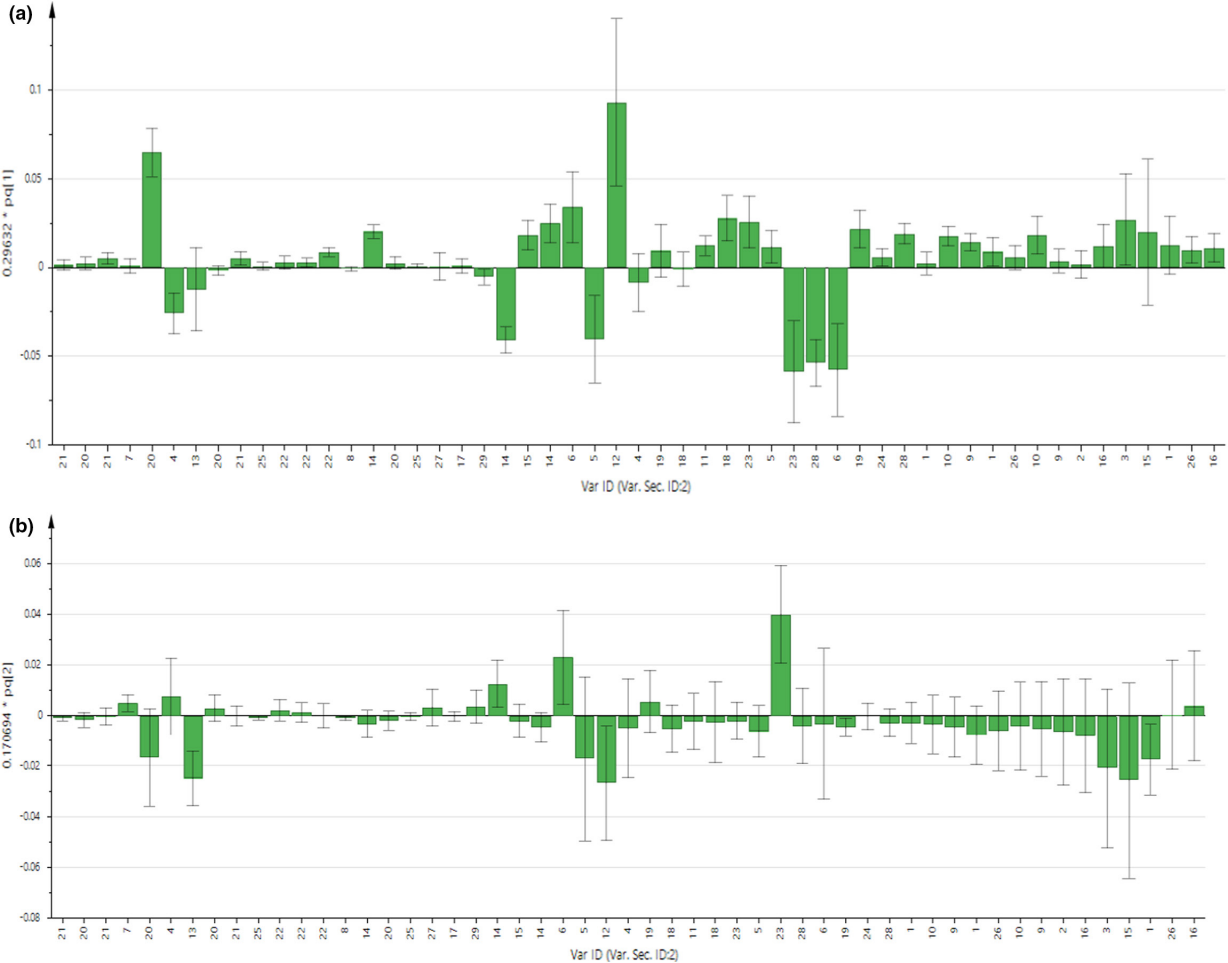
(a,b) OPLS‐DA loading column plots show the contribution of metabolites in the PM muscle for breeds clustering based on PC1 and 2 in score and loading plots, respectively. Key signals: 1. 3‐hydroxybutyrate, 2. Acetate, 3. Alanine, 4. Anserine, 5. Betaine, 6. Creatine, 7. Formate, 8. Fumarate, 9. Glutamate, 10. Glutamine, 11. Glycine, 12. Guanidoacetate, 13. Hypoxanthine, 14. IMP, 15. Lactate, 16. Leucine, 17. Mannose, 18. Myo‐inositol, 19. N,N‐dimethylglycine, 20. NAD+, 21. Niacinamide, 22. Phenylalanine, 23. Taurine, 24. Trimethylamine, 25. Uracil, 26. Valine, 27. α‐glucose, 28. β‐alanine, and 29. β‐glucose

As displayed in Figures 2 and 3, the contribution of each metabolite to the separation of four chicken breeds for chicken serum and PM muscle was illustrated in the respective loading column plots with jackknife error bars. Metabolite variables with valuable influence on projection (VIP) value >1, and jackknife error bar do not cross zero in the respective OPLS‐DA loading column plot were assigned as discriminating metabolites. The correlation of the discriminating metabolites to the chicken breeds clustering in the OPLS‐DA score plot for both matrices was further shown in the respective OPLS‐DA loading plots (Figure 1c,d). All discriminating metabolites for chicken serum and PM muscle are listed in Tables S2 and S3, respectively.

In Figure 1c for chicken serum, based on principal component (PC) 1 and PC2, 11 discriminating metabolites were correlated with the AVC cluster, including 3‐hydroxybutyrate, citrate, creatine, glutamine, glycine, lactate, leucine, lysine, malate, methionine, and succinate. Furthermore, another two discriminating metabolites (i.e., acetone and valine) were correlated with the DSL cluster. Besides that, based on PC 2, metabolic components of α‐glucose, alanine, betaine, homoserine, myo‐inositol, and phenylalanine were correlated with the HCB and CBC cluster. Meanwhile, based on PC1 and PC2 as displayed in Figure 1d for PM muscle, several metabolic components such as β‐alanine, anserine, hypoxanthine, lactate, and IMP were correlated with the AVC cluster. Besides, based on PC2, metabolic components of taurine, phenylalanine, and creatine were correlated with the cluster of HCB and DSL, while alanine, betaine, glycine, myo‐inositol, N,N‐dimethylglycine, guanidoacetate, and NAD+ were correlated with the CBC cluster.

### 3.4 Semi‐quantitation of samples

Semi‐quantitation results of all metabolites identified in chicken serum and PM muscle were determined, as reported in Tables S4 and S5, respectively. The comparison of semi‐quantitation values between any two chicken breeds from different clustering in OPLS‐DA models is displayed in Tables S6 and S7.

## 4 DISCUSSION

^1^H NMR coupled with multivariate analysis through OPLS‐DA has successfully discriminated the four commercial chicken breeds in this study. Applying OPLS‐DA in the present study reduces the technical bias under validation conditions by statistical parameters (Eriksson et al., 2006). The permutation tests of the OPLS‐DA models showed the validity of these statistical calculations. Besides, in PCA and OPLS‐DA score plots, all breeds were clustered into three main groups for the chicken serum and PM muscle, respectively, as displayed in Figures 1 and S6. Furthermore, our results showed that clustering trends of all chicken breeds for serum and PM muscle in OPLS‐DA models were consistent and in agreement with the PCA models. Interestingly, for both chicken serum and PM muscle matrices, distinct clustering was observed for the AVC breed from other commercial breeds in OPLS‐DA score plots, confirming the suitability and applicability of both matrices in AVC breed discrimination under the NMR platform. Nonetheless, the present result showed that HCB was indistinguishable from CBC and DSL for the chicken serum and PM muscle, respectively. In this study, all commercial chicken breeds were procured directly from local commercial farms according to the individual birds' marketing age, reflecting the market condition, as summarized in Table 1. For instance, the difference in marketing age between the commercial breeds could lead to variation in metabolism and metabolic composition (Jayasena, Jung, Kim, et al., 2015; Xiao et al., 2019). Nonetheless, all chicken breeds were raised under similar standard conditions and fed with the same brand of commercial feeds (Gold Coin). In the present study, serum matrix could be a better option compared to PM muscle for chicken breeds discrimination due to the simplicity of serum sample extraction and high throughput under the NMR platform (Zhu et al., 2019). From the multivariate data analysis, the contribution of the discriminating metabolites to the differentiation of chicken breeds was further supported by the semi‐quantitation results obtained in the CPMG spectra. In previous studies, the internal standard of TSP has been applied for the semi‐quantitation of the identified metabolites in the CPMG spectra of muscle and serum (Behan et al., 2021; Nagana Gowda et al., 2018; Nagesh Babu et al., 2018; Soglia et al., 2019; Zampiga et al., 2021). The present semi‐quantitation results for serum and pectoralis major successfully demonstrated and verified the correlation of the discriminating metabolites with the respective chicken breeds in the OPLS‐DA models.

Village chicken is perceived by consumers worldwide as superior meat with better taste, nutrition, and meat quality compared to other fast‐growing chicken meat (Choe et al., 2010; Mohd Shahmi Hakimi et al., 2019; Zotte et al., 2020). For instance, inosine monophosphate (IMP), a flavor precursor related to the umami taste, has been reported as a characteristic metabolite for slow‐growing native chickens in several countries such as China, Japan, and Korea (Jung et al., 2011; Rikimaru & Takahashi, 2010;Xiao et al., 2021; Zhang et al., 2018). In previous studies, several Chinese native chicken breeds, such as Lueyang black‐bone native chicken, Wuding chicken, and Yanjin silky fowl chicken, were found to have a high amount of IMP in the breast meat (Xiao et al., 2021; Zhang et al., 2018). Similar results were also reported for commercial Korean native chicken (*Woorimatdag*™) and Hinai‐jidori chicken, a crossbreed of Hinai‐dori native chicken with Rhode Island Red breed in Japan (Jung et al., 2011; Rikimaru & Takahashi, 2010). Intriguingly, consistent with previous findings, IMP and hypoxanthine were positively correlated with the AVC for PM muscle. IMP is one of the degradation products related to adenosine‐5′‐triphosphate (ATP) after slaughtering. For instance, ATP is unstable in muscle and prone to be degraded into adenosine diphosphate (ADP), adenosine monophosphate (AMP), and IMP. Moreover, IMP was further degraded into hypoxanthine and inosine (Mabuchi et al., 2018). Based on the semi‐quantitation result, both IMP and hypoxanthine contents in AVC had 3‐ to 5‐folds and 2‐ to 4‐folds greater than CBC, DSL, and HCB, respectively. Thus, these current results indicated that both metabolites are effective metabolites in PM muscle for discriminating AVC from other breeds.

From the results of the multivariate analysis, the AVC cluster for PM muscle was correlated with an important biomarker of anserine, a bioactive histidine dipeptide. Anserine is a derivative of carnosine, which is present naturally in poultry and shows therapeutic effects similar to carnosine, such as antioxidant, antiaging, and pH buffering in muscle (Jung et al., 2013; Łukasiewicz et al., 2015). In this study, anserine content in AVC was in good agreement with approximately 5‐ to 6‐folds higher than other chicken breeds. For instance, recent studies have shown that native chicken in Thailand and Korea contains a significantly high amount of anserine in the breast meat (Ali et al., 2019; Charoensin et al., 2021). Furthermore, a study (Charoensin et al., 2021) has proposed that Thai native chicken is a functional meat source for bioactive dipeptides, including anserine and carnosine. Meanwhile, Ali et al. (2019) reported that the anserine content of commercial Korean native chickens was significantly higher than commercial broilers in the comparison study between both breeds in Korea. These findings indicate that this dipeptide could be a potential metabolic biomarker for AVC.

Interestingly, both clusters of AVC in chicken serum and PM muscle were correlated with lactate. According to Wang et al. (2018), the chicken soup of two native chicken breeds (black‐bone silky and Sanhuang breed) in South China was detected with lactate as the predominant organic acid. Besides, previous studies also reported a positive correlation between the lactate content and animals' exercise and locomotor activities (Mabuchi et al., 2019; Zotte et al., 2020). Therefore, AVC, which is more physically active than other commercial chicken breeds in the present study, may lead to lactate accumulation in chicken serum and PM muscle. Additionally, lactate formation and accumulation were associated with glycolysis and post mortem handling condition (Mukhtar et al., 2021). Nonetheless, the PM muscle of all chicken breeds in this study was subjected to immediate snap freezing after the sampling to prevent further post mortem degradation. Thus, different deterioration rates involved between chicken breeds may explain this finding.

As displayed in the loading plots (Figure 1c) for chicken serum, the cluster of AVC was predominantly correlated with several major amino acids such as glutamine, glycine, leucine, lysine, and methionine. In addition, the content of these amino acids was further quantified to confirm their contribution and consistency in OPLS‐DA models. Furthermore, the present results showed the potential of these metabolites as discriminating metabolites in the serum of AVC. On the other hand, for PM muscle samples, our results in Figure 1d show that the CBC cluster and HCB and DSL cluster were also correlated with several amino acids, including glycine and phenylalanine, respectively. For instance, given the actual condition of chicken meat sold in the local markets, potential candidate metabolites for chicken breed discrimination should be present in high amounts in the samples with no significant changes at different storage times and temperatures. Nonetheless, previous studies have shown that the amino acid profile of chicken meat was affected by different temperature and storage conditions (i.e., frozen and refrozen) (Mohammed et al., 2021; Triki et al., 2018; Wen et al., 2020). For example, a study by Triki et al. (2018) found that both amino acids' content of glycine and phenylalanine in chicken breast meat changed significantly at different storage periods under chilled conditions. Besides, the study by Mohammed et al. (2021) showed that phenylalanine content in chicken meat was increased after the freeze–thaw cycles while glycine remained unaffected. Therefore, further studies on the amino acid profiles of chicken meat at different storage times and conditions, reflecting the market condition, are warranted to further confirm the potential of these amino acid metabolites in PM muscle meat for chicken breeds discrimination.

The present NMR‐based metabolomics result demonstrated the contribution of myo‐inositol and betaine to the cluster of CBC in both chicken serum and PM muscle. For instance, myo‐inositol is contained naturally in maize, nuts, and beans (Moroni et al., 2021). Therefore, consistent with expectation, all four breeds fed with similar corn and soybean‐based commercial feeds were detected with myo‐inositol in chicken serum and PM muscle, respectively. Meanwhile, betaine is an alpha‐amino acid that naturally occurs in chickens, and plays an essential role in chicken metabolism (Jayasena, Jung, Bae, et al., 2015; Jayasena, Jung, Kim, et al., 2015; Lengkidworraphiphat et al., 2021; Saeed et al., 2017). In a previous comparison study between commercial broiler and Korean native chicken, the content of betaine in broiler muscle was reported significantly higher than in Korean native chicken (Jayasena, Jung, Bae, et al., 2015; Jayasena, Jung, Kim, et al., 2015). Similarly, Lengkidworraphiphat et al. (2021) also reported that broiler breast muscle had a higher betaine content than other native chicken breeds in Thailand. In the present study, PM muscle of CBC had betaine content of approximately 4‐, 3‐, and 6‐fold higher than AVC, HCB, and DSL, respectively. Besides, the betaine content in serum for CBC was approximately 2‐ and 3‐fold greater than AVC and DSL, respectively. Furthermore, our results showed that the DSL breed with a marketing age of approximately 72 weeks had the lowest betaine content compared to other breeds, which agrees with both authors, who reported a decrease in betaine content uptake with age. Thus, the present results for chicken serum and PM muscle could indicate the potential of betaine as an effective biomarker for CBC. It is also worth noting that the cluster of CBC for PM muscle was correlated with guanidoacetate, which occurs naturally in chicken (Dilger et al., 2013). Nevertheless, according to European Food Safety Authority, guoanidoacetate has been approved as a feed additive for the fatting purposes of chicken (EFSA, 2009). In addition, several studies have been conducted on dietary supplementation of guanidoacetate in commercial feeds for better growth performance of chicken production in recent years (de Souza et al., 2021; Khajali et al., 2020). Taken together, further studies are required to confirm the suitability of guanidoacetate as a candidate biomarker for CBC.

## 5 CONCLUSION

The present study successfully discriminated AVC from the other three commercial breeds (CBC, HCB, and DSL) by employing an NMR‐based untargeted metabolomics study. A clear separation for four different chicken breeds was observed in multivariate analysis through OPLS‐DA models. Subsequently, the discriminating metabolites for AVC and the other three breeds were identified in chicken serum and PM muscle, respectively. These characteristic metabolites are effective metabolites that could be utilized in discriminating different chicken breeds in markets. In conclusion, the overall results provide useful metabolic pattern information regarding commercial chicken breed discrimination and serve as baseline data for future investigation and monitoring by respective agencies. Additionally, the present results could be applied as a complementary analysis with other omic approaches, including genomic and proteomic as a practical test for chicken breed discrimination.

## CONFLICT OF INTEREST

The authors declare that they do not have any conflict of interest.

## ETHICAL APPROVAL

This study was approved by the Institutional Animal Care and Use Committee, Universiti Putra Malaysia (Approval number: UPM/IACUC/AUP‐R022/2019, Dated May 23, 2019).

## Supporting information


Figure S1

## Data Availability

The data that support the findings of this study are available in the Supplementary Material of this article.
