# Supplementary material for: 1H nuclear magnetic resonance‐based metabolomics study of serum and pectoralis major for different commercial chicken breeds
Source: Food Sci Nutr. 2023 Apr 7;11(5):2106–17. doi: 10.1002/fsn3.2968 (PMC10171504; doi:10.1002/fsn3.2968)
Supplement: Supplementary file 1 — Figure S1 [file FSN3-11-2106-s001.docx]

Supplementary Documents

**Figure S1** Representative 700 MHz ^1^H NMR spectra for chicken serum and PM

**Figure S2** Chemical structure of characteristic metabolites identified in chicken serum

**Figure S3** Chemical structure of characteristic metabolites identified in pectroralis major muscle

**Figure S4** 700 MHz 2D J-resolved NMR spectra for metabolites identified in chicken serum

**Figure S5** 700 MHz 2D J-resolved NMR spectra for metabolites identified in PM

**Figure S6** PCA score plots for chicken serum and PM

**Figure S7** Permutation test validation plots of OPLS-DA model for chicken serum

**Figure S8** Permutation test validation plots of OPLS-DA model for PM muscle

**Table S1** R^2^Y and Q^2^Y intercept values of OPLS-DA model permutation test

**Table S2** VIP values of characteristic metabolites in chicken serum

**Table S3** VIP values of characteristic metabolites in PM muscle

**Table S4** Semi-quantitation on metabolites identified for chicken serum (mM)

**Table S5** Semi-quantitation on metabolites identified for PM muscle (mM)

**Table S6** Comparison of relative intensity mean values of ^1^H resonances for metabolites in chicken serum from any two chicken breeds based on the clustering

**Table S7** Comparison of relative intensity mean values of ^1^H resonances for identified metabolites in PM muscle from any two chicken breeds based on the clustering


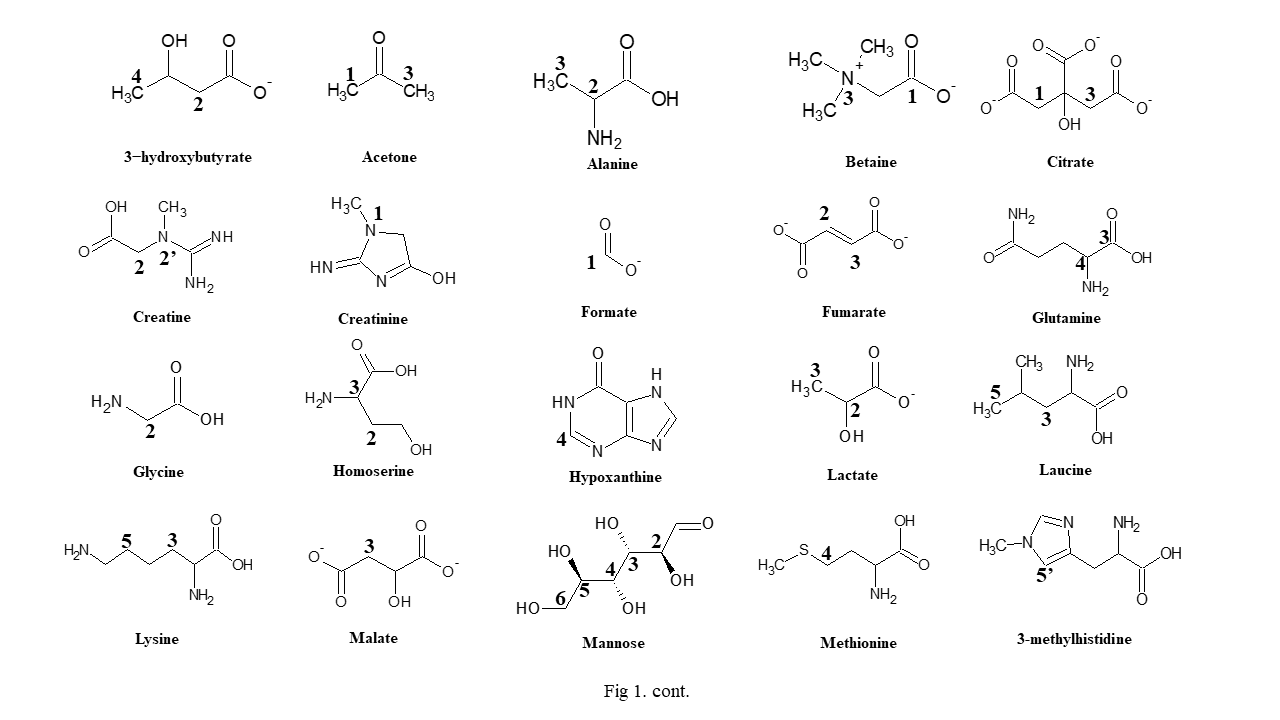


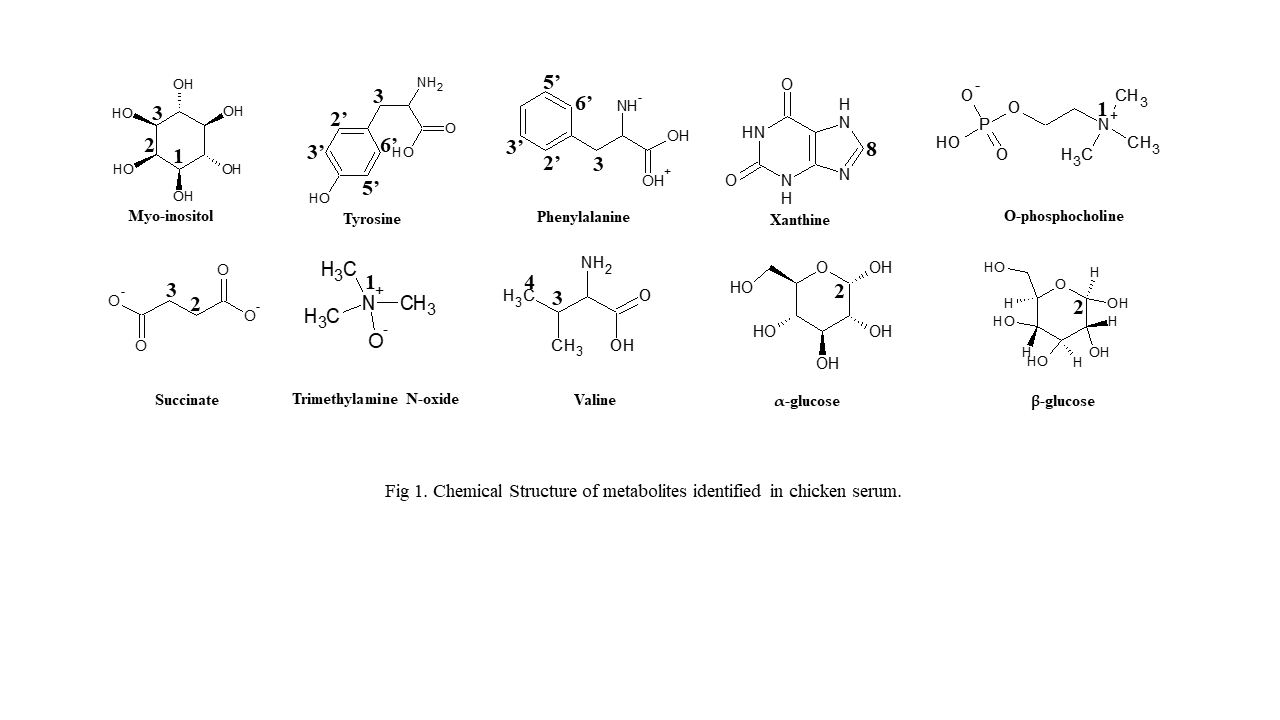


**FIGURE 1** Chemical structures of identified characteristic metabolites in chicken serum


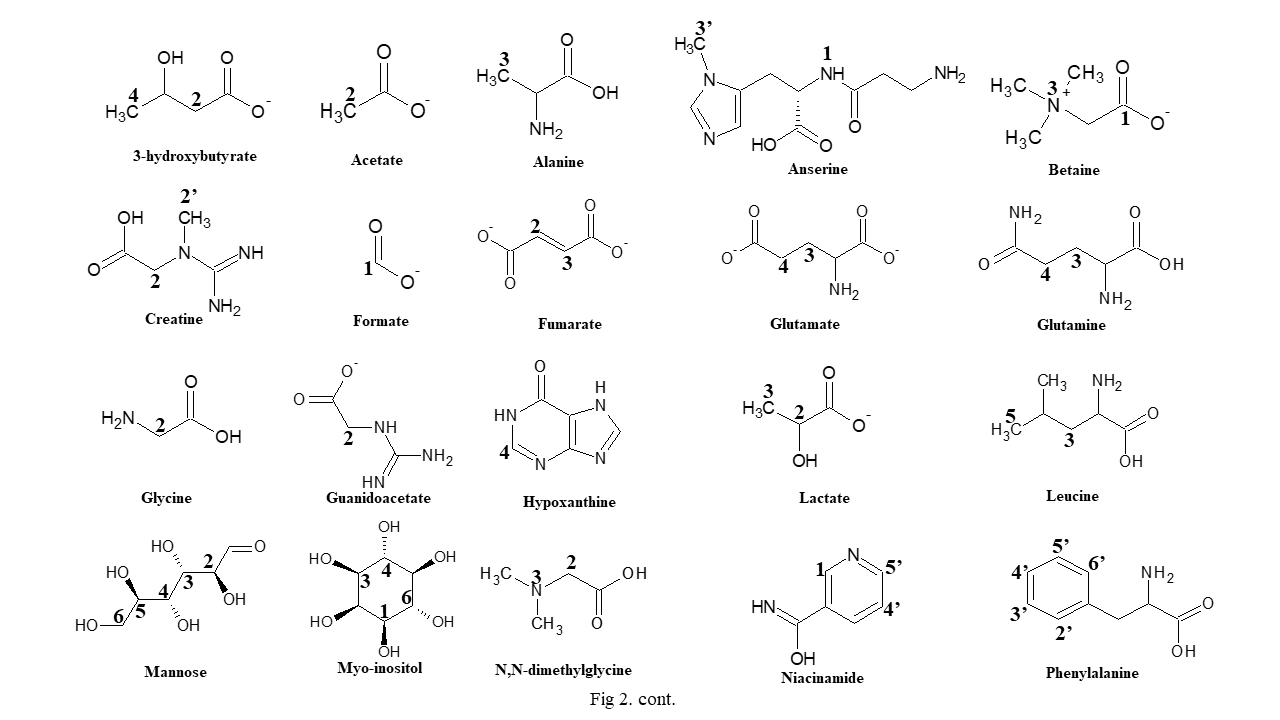


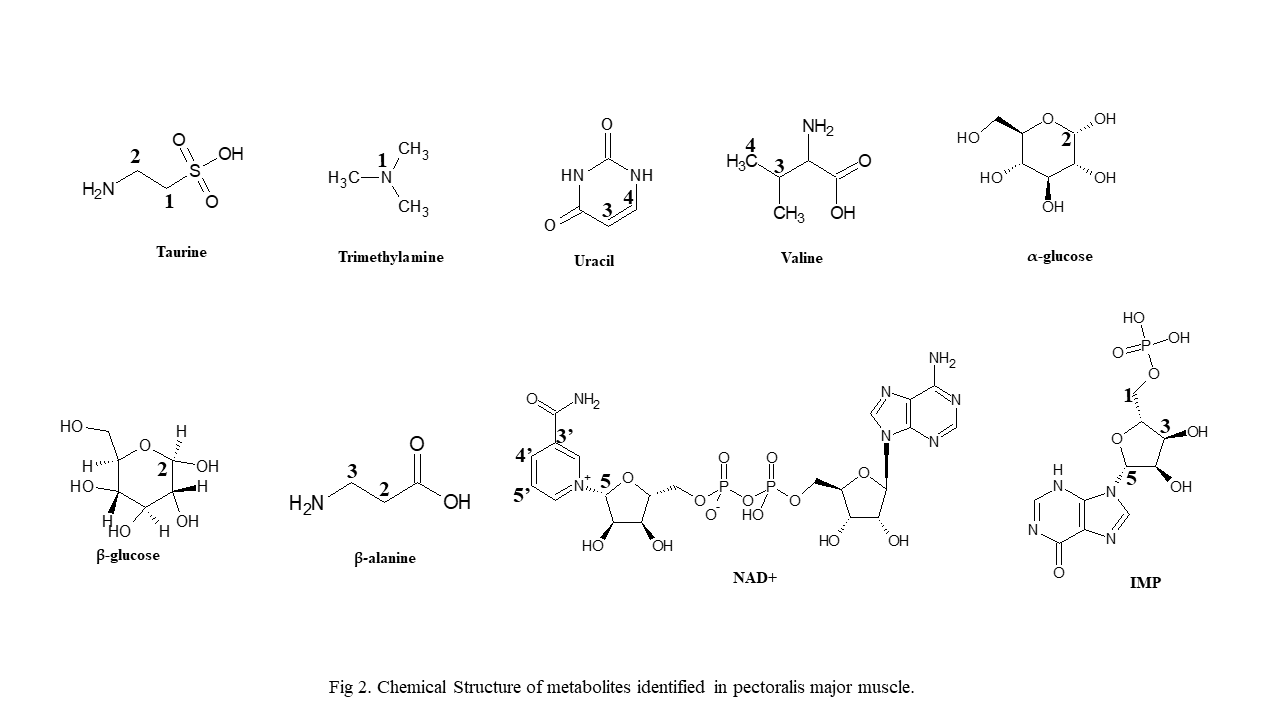


**FIGURE 2** Chemical structures of identified characteristic metabolites in pectoralis major muscle


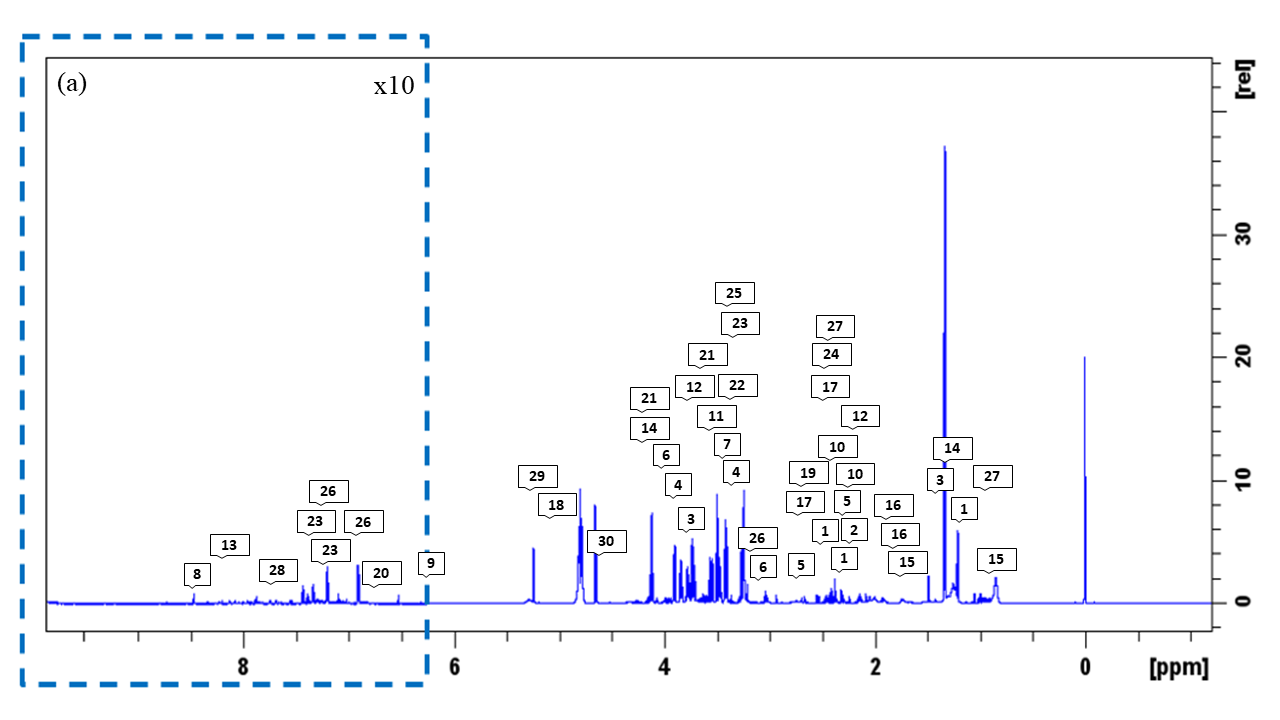


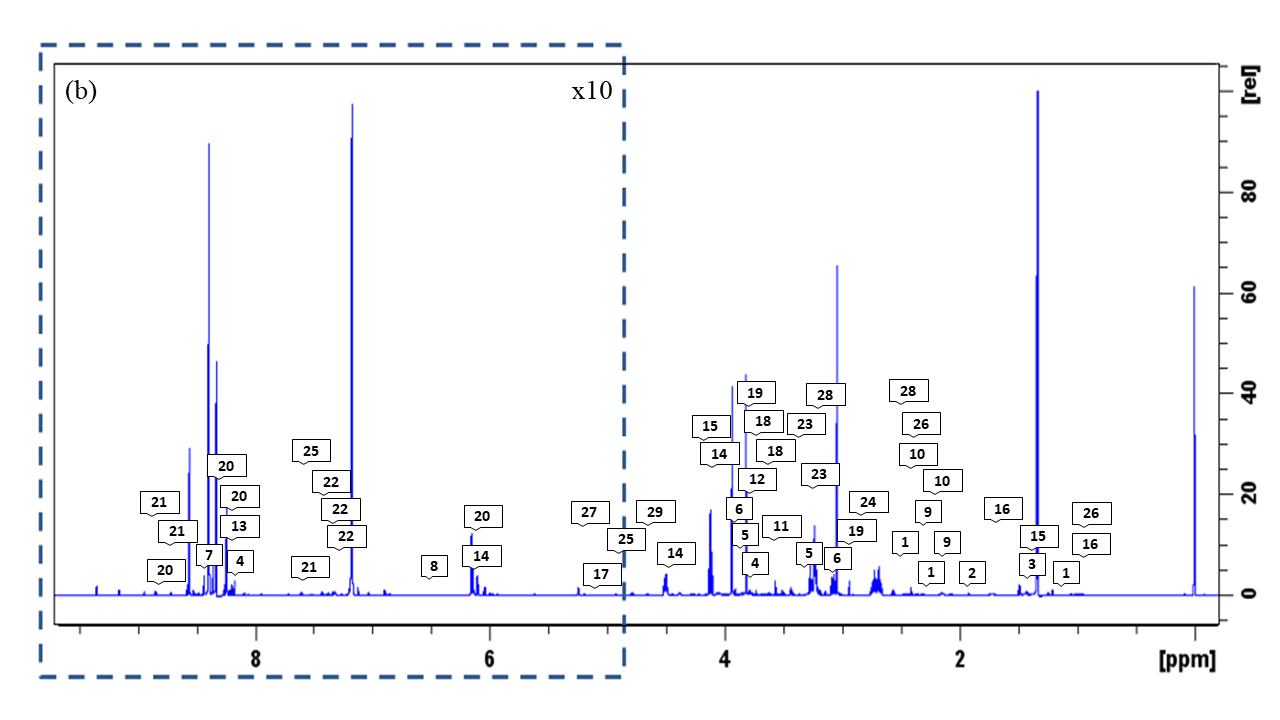


**FIGURE S3** Representative 700 MHz ^1^H NMR spectra for serum (a) and pectoralis major (b) of different chicken breeds. All spectra were referenced to the resonance of TSP at 0.00 ppm


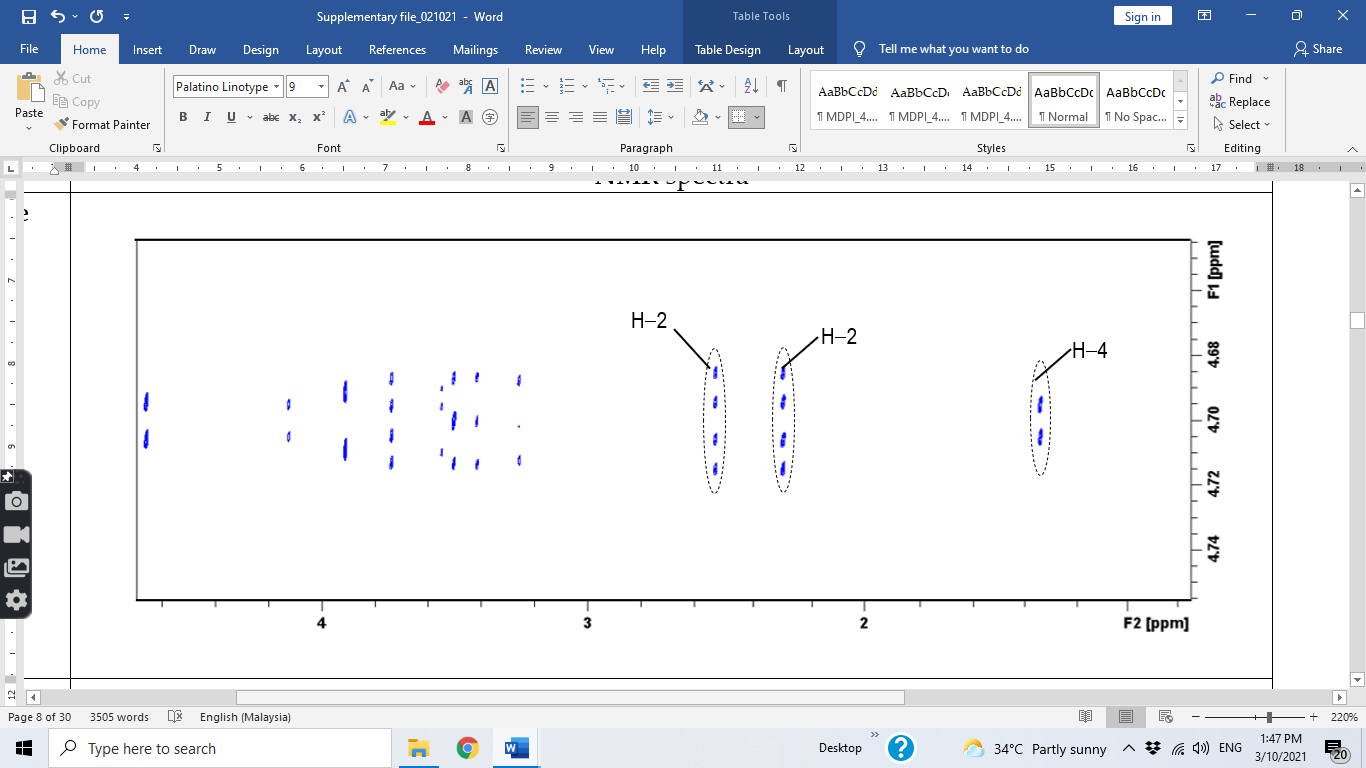

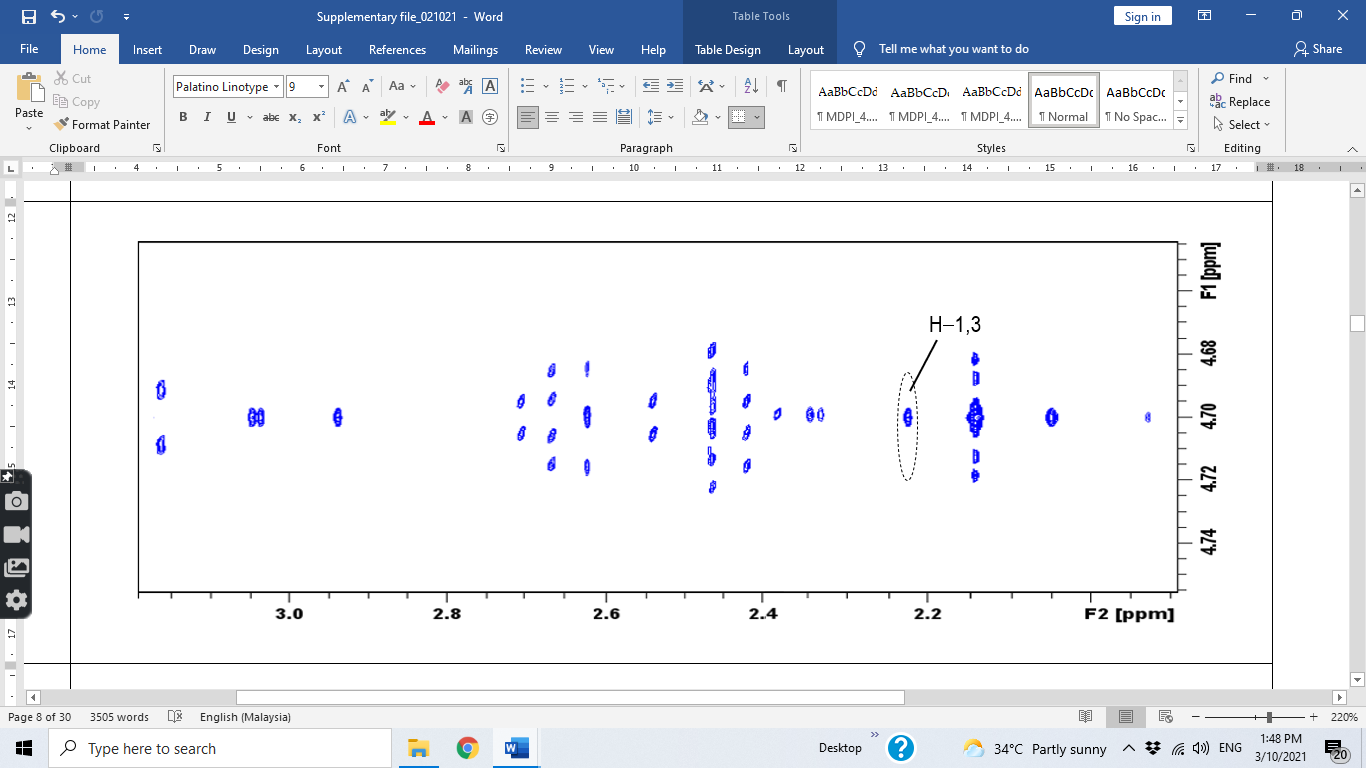


3-hydroxybutyrate Acetone


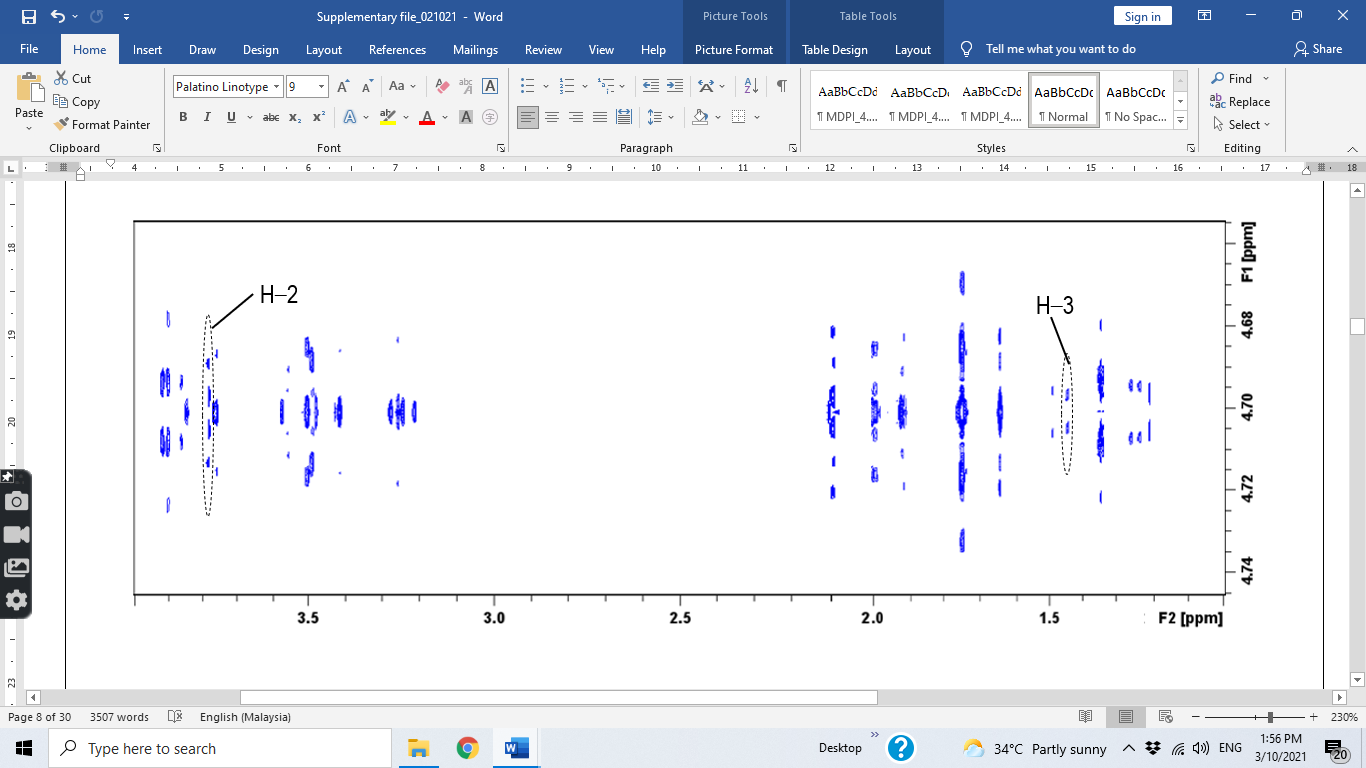

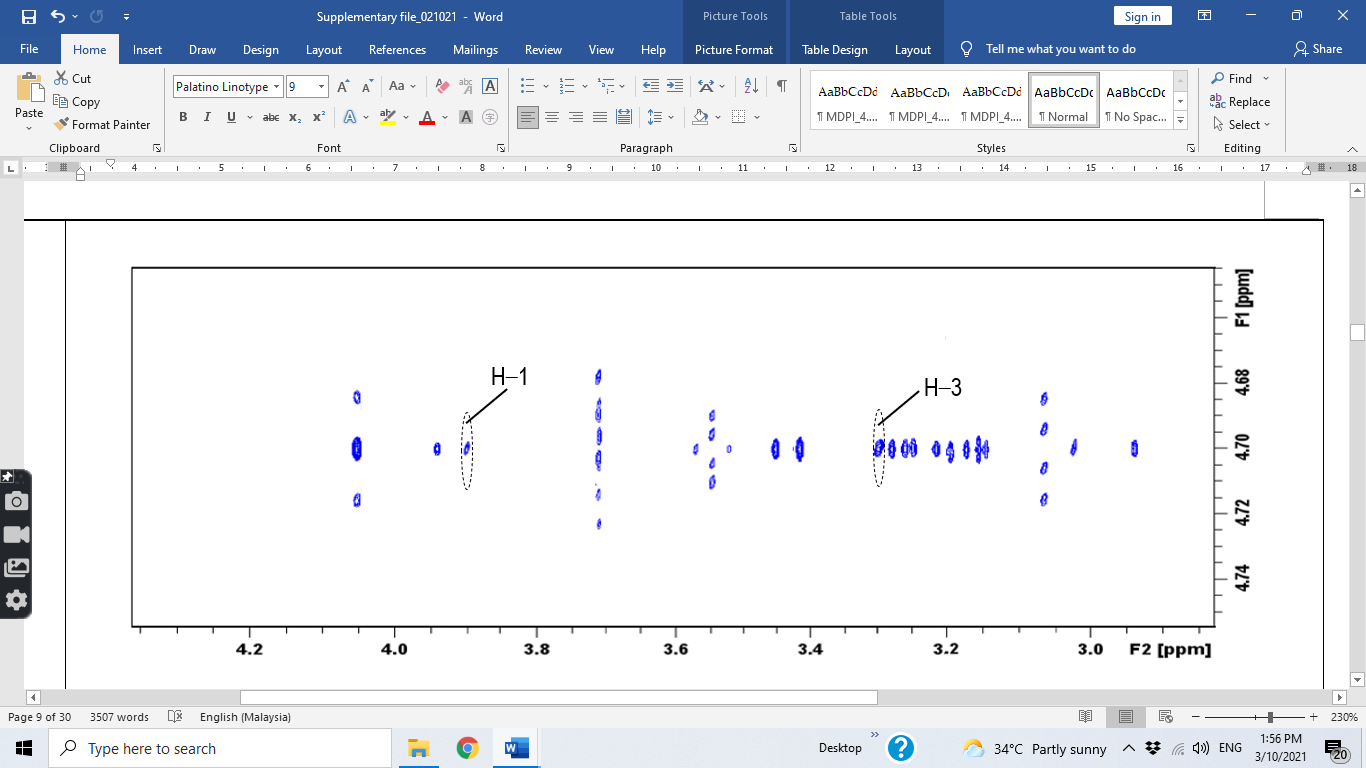


Alanine Betaine


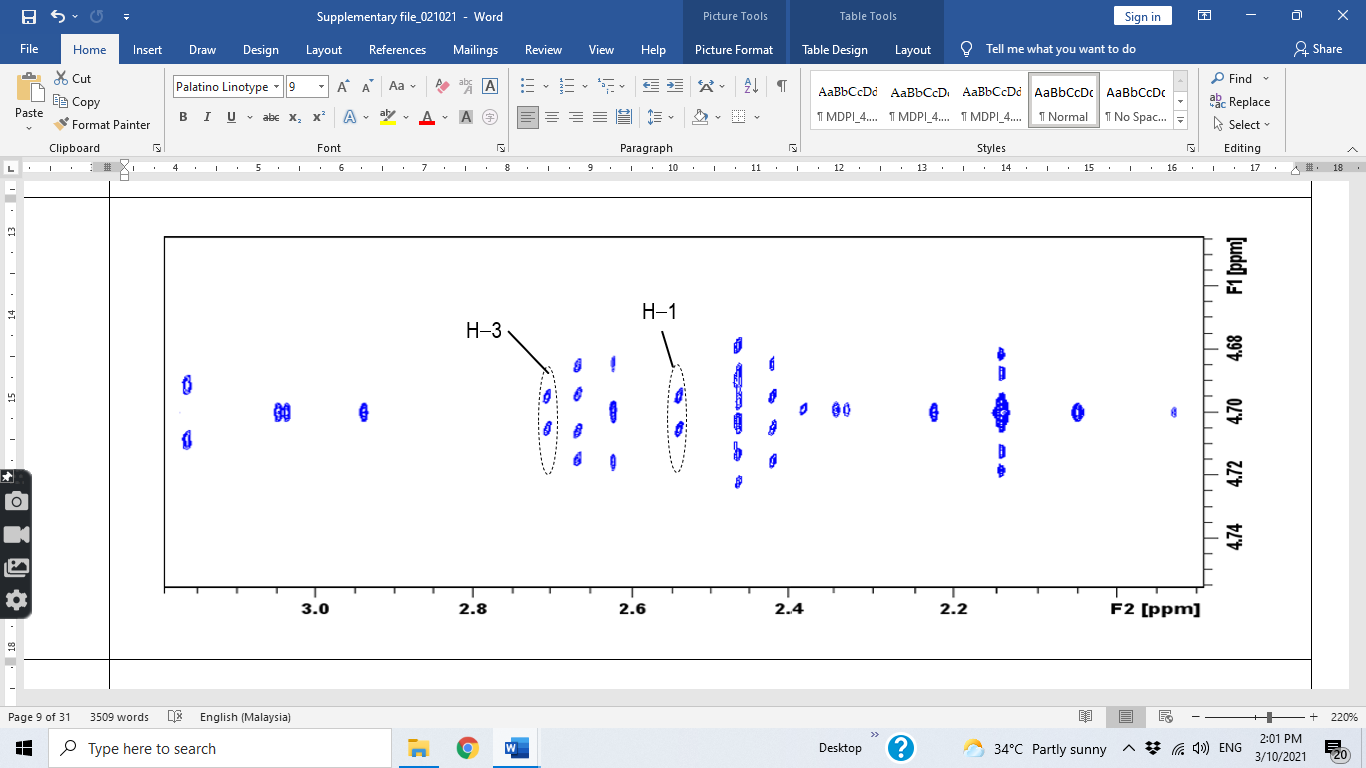

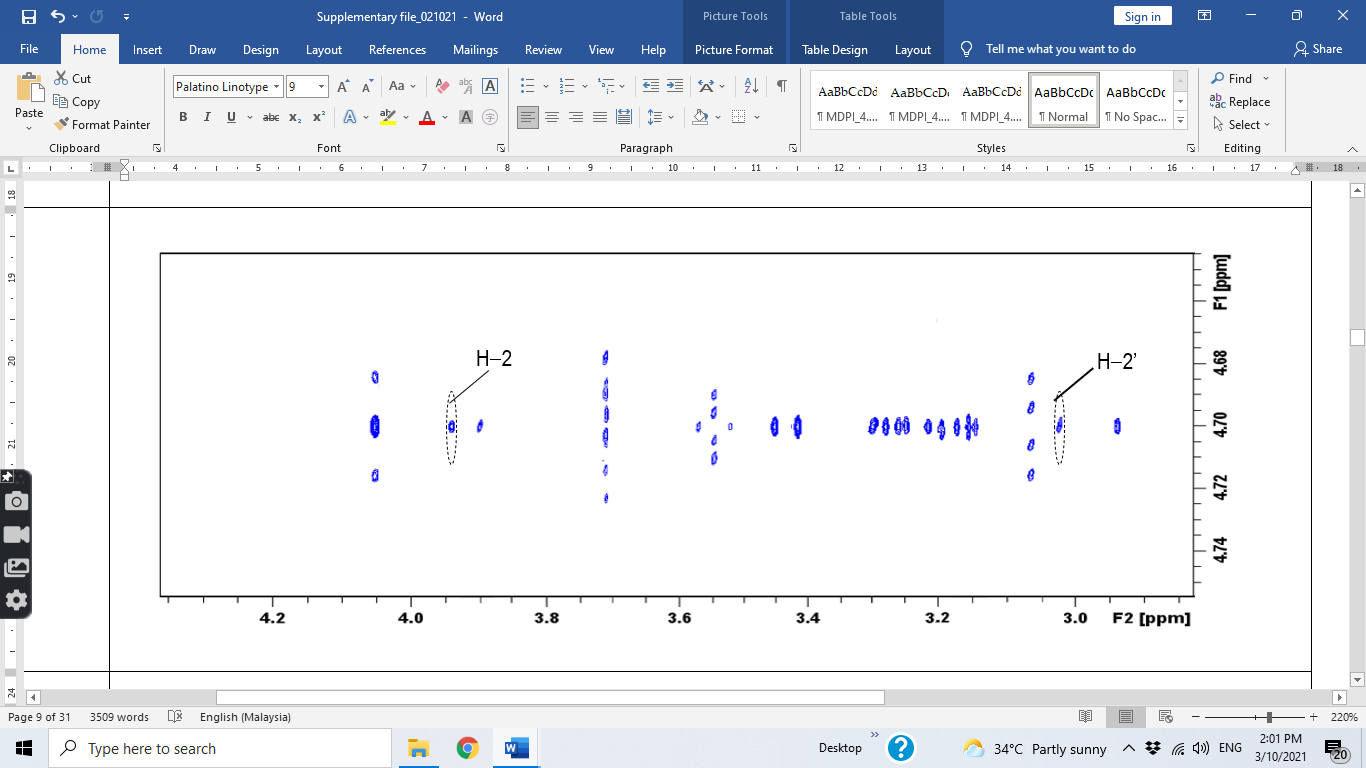


Citrate Creatine


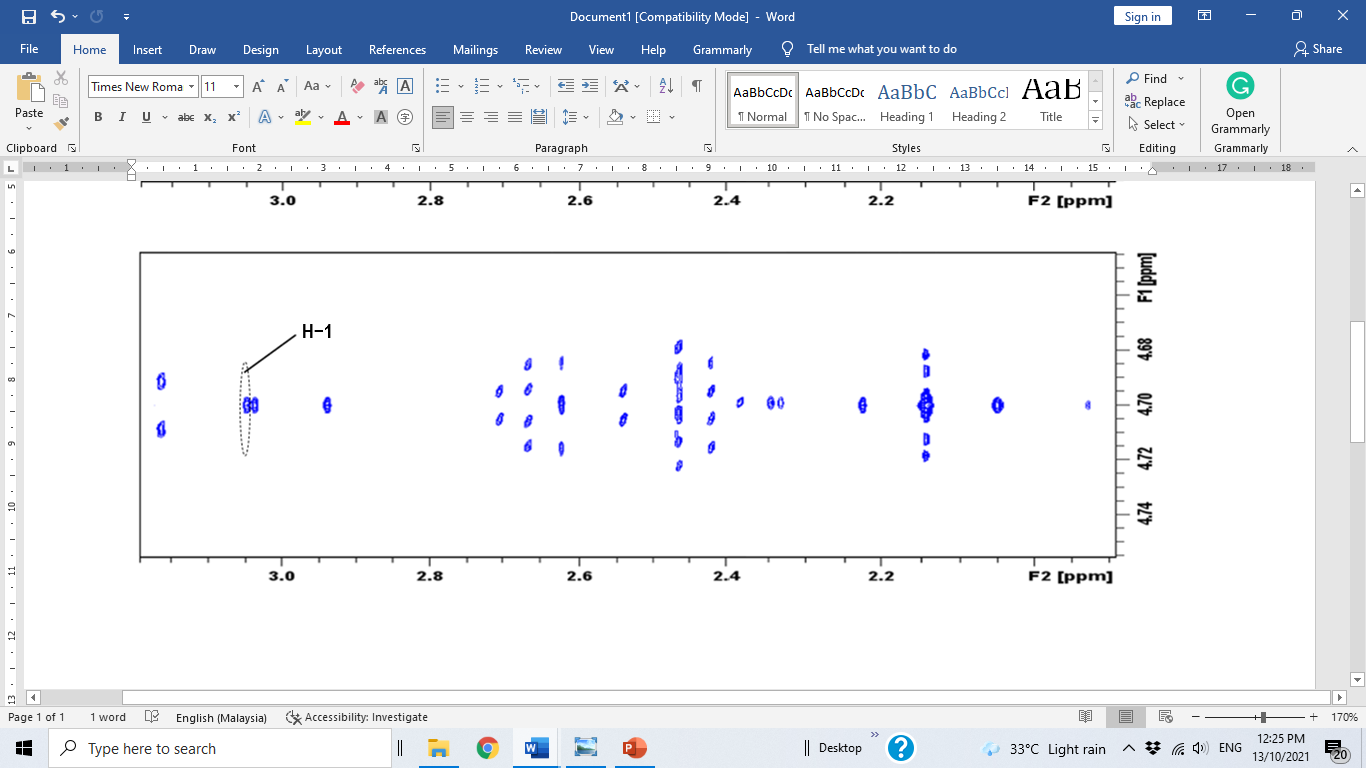

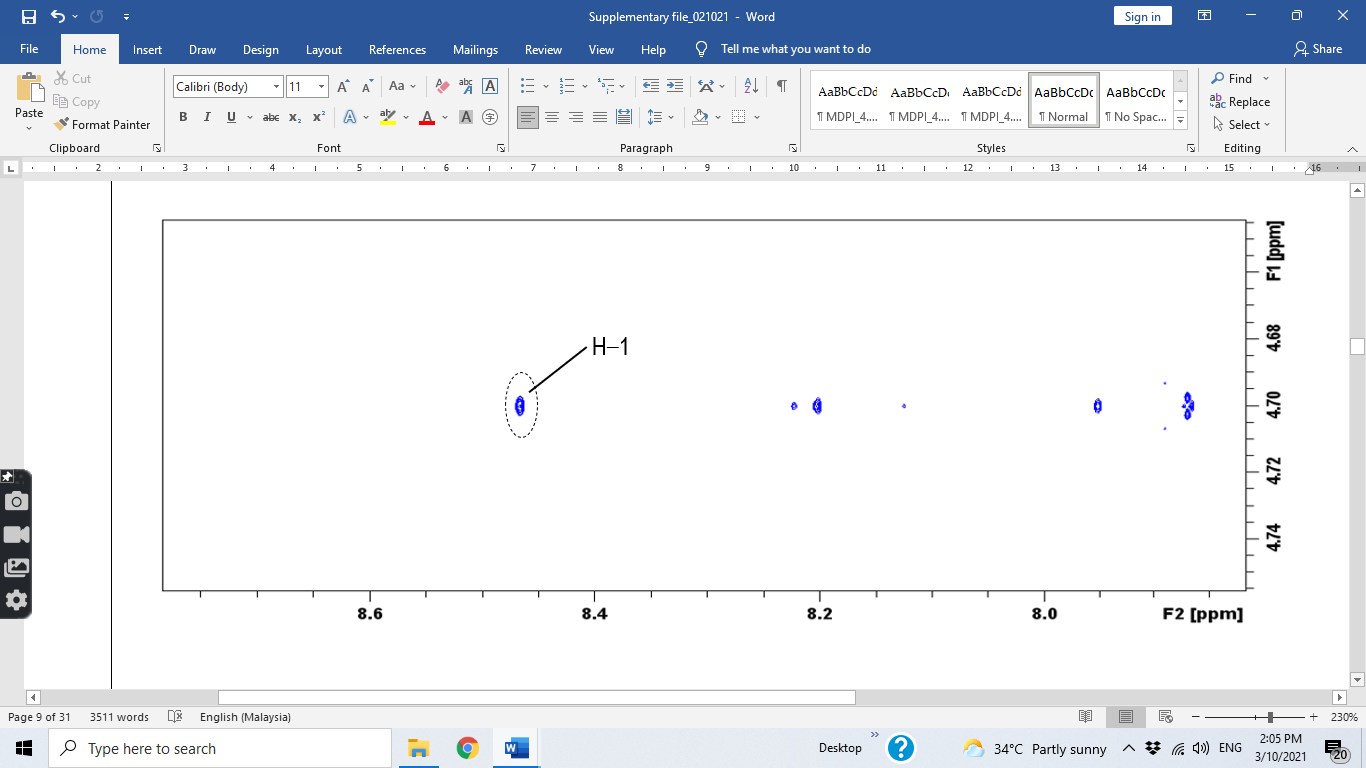


Creatinine Formate


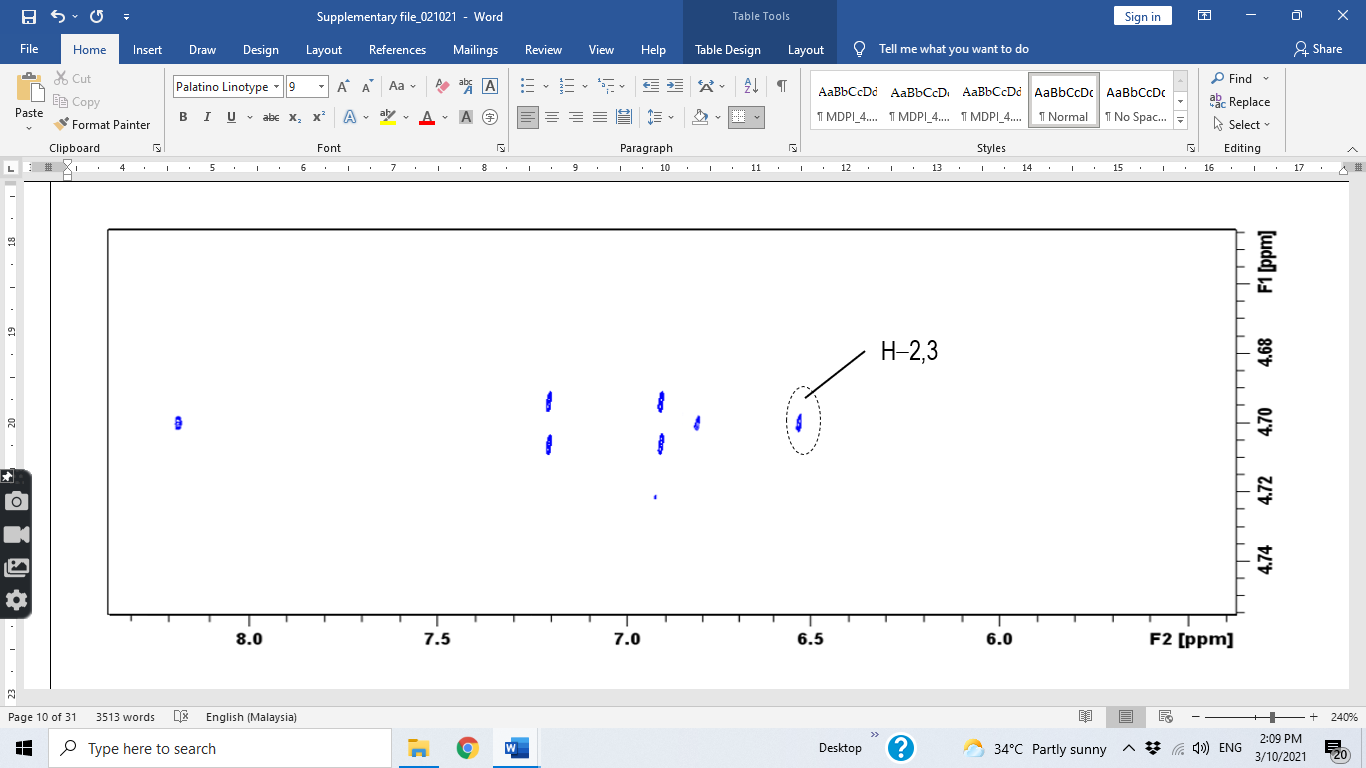

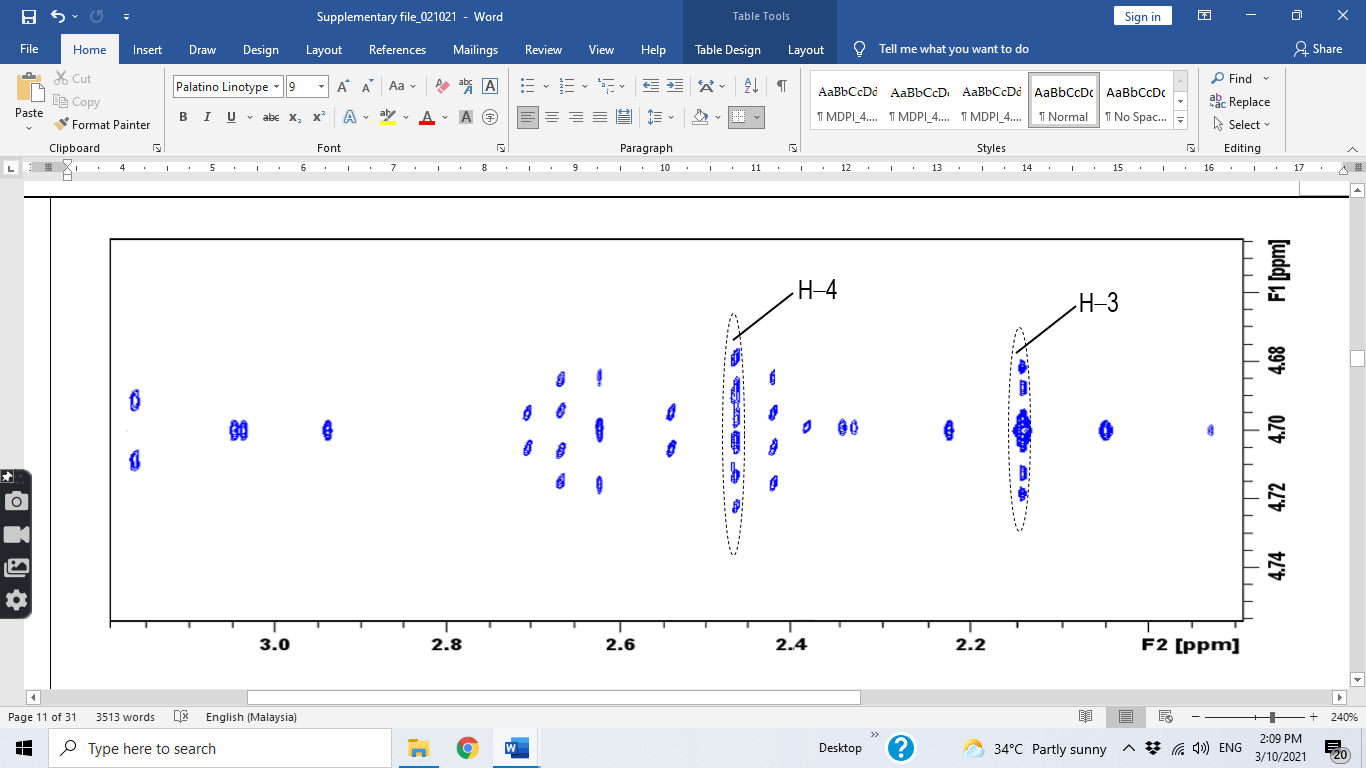


Fumarate Glutamine


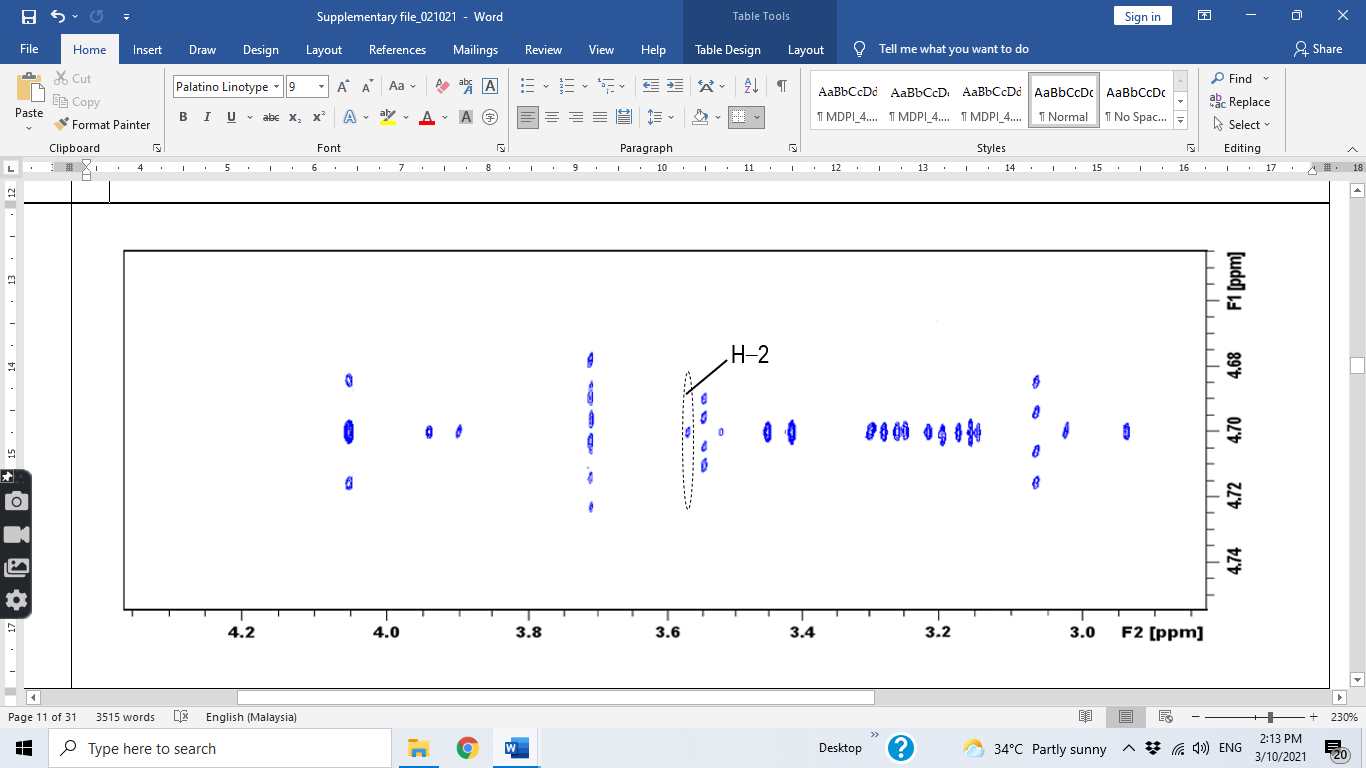

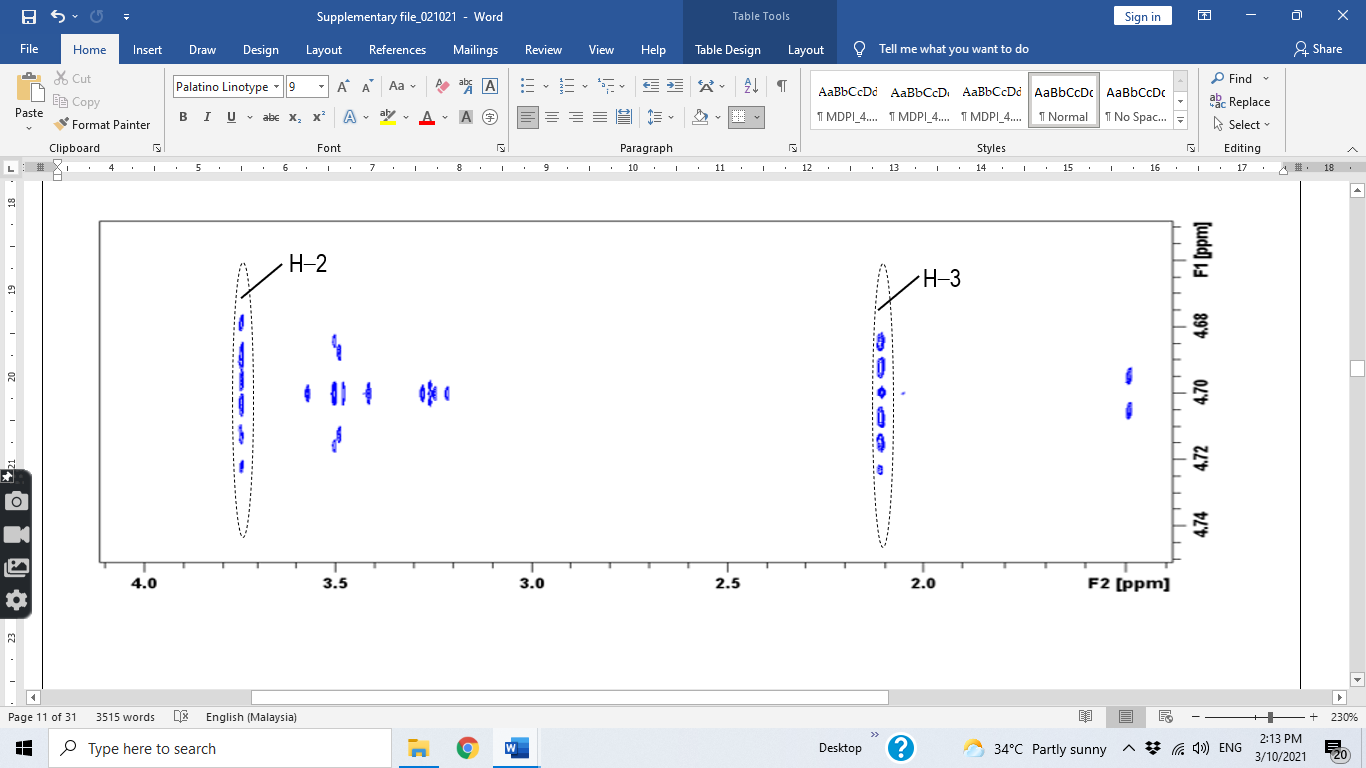


Glycine Homoserine


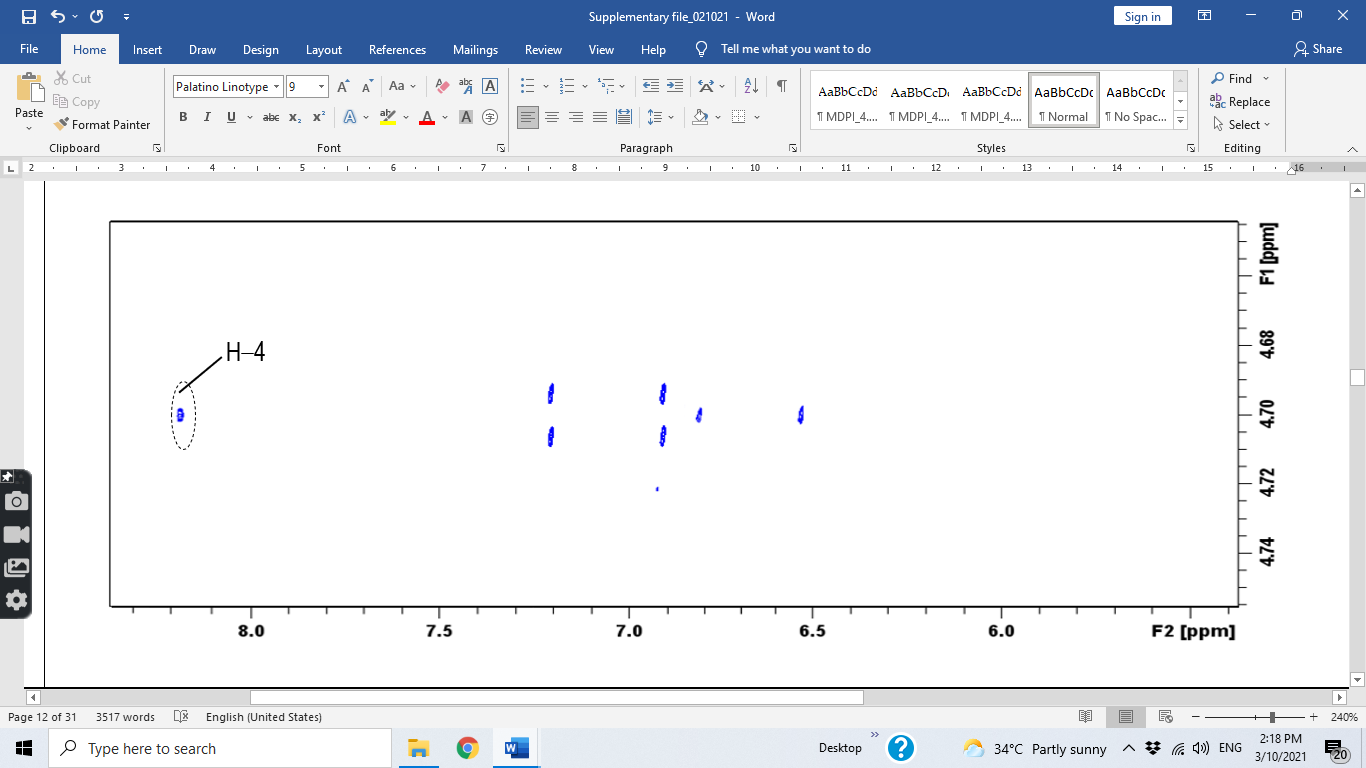

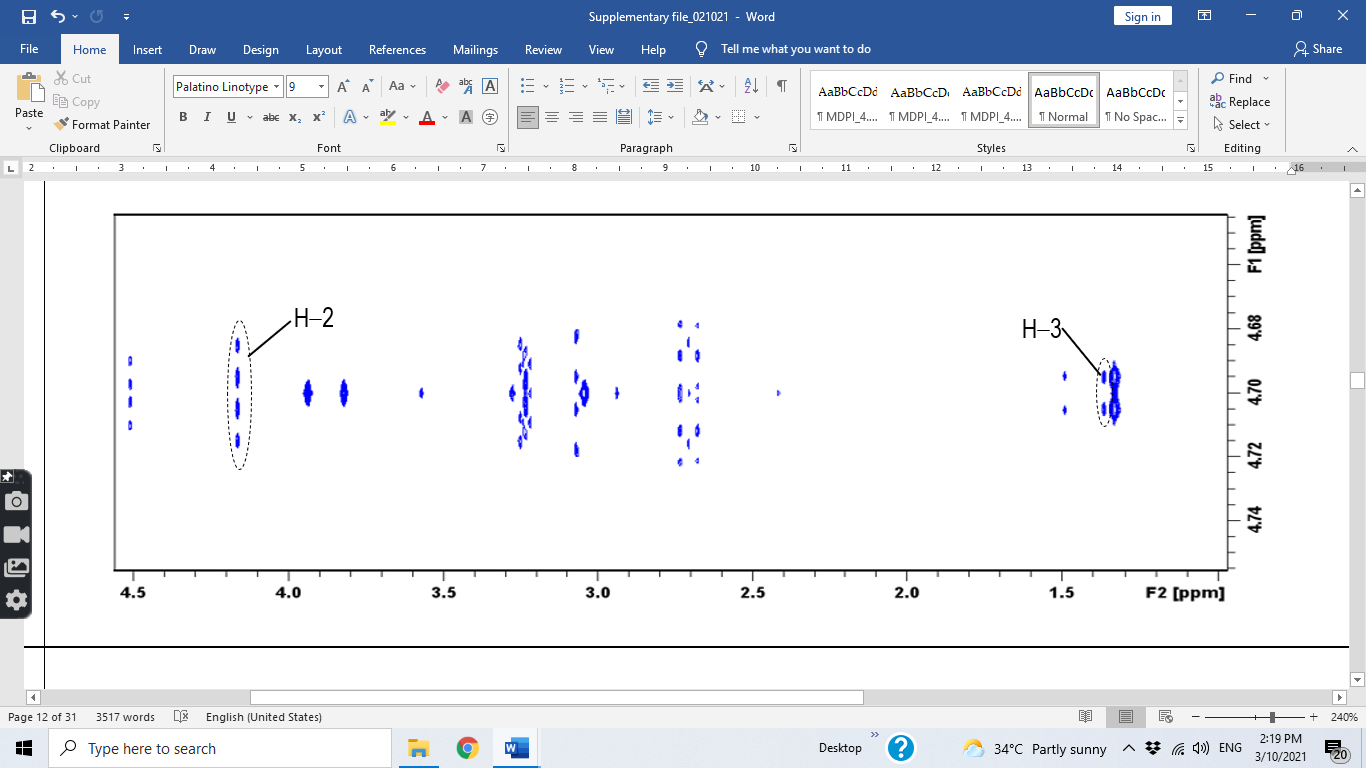


Hypoxanthine Lactate

**FIGURE** **S4 (**Continued)


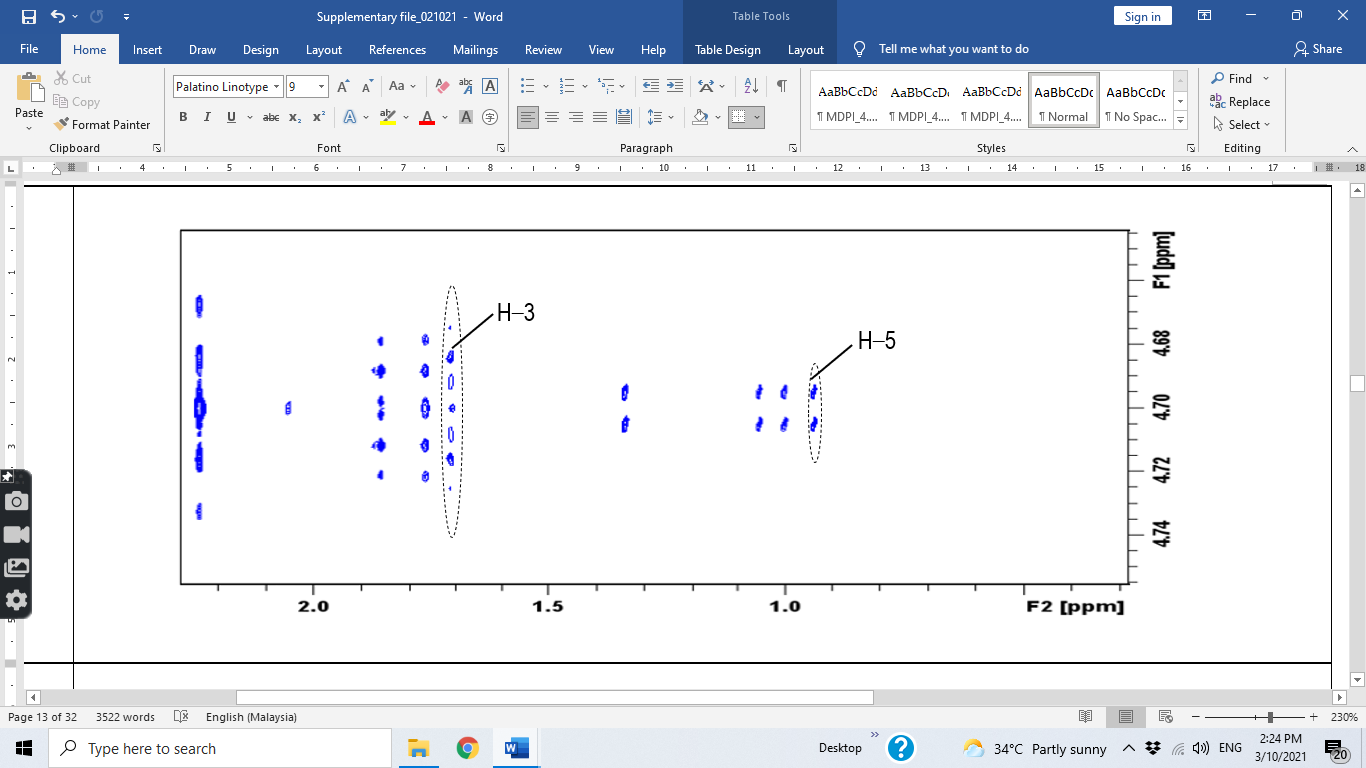

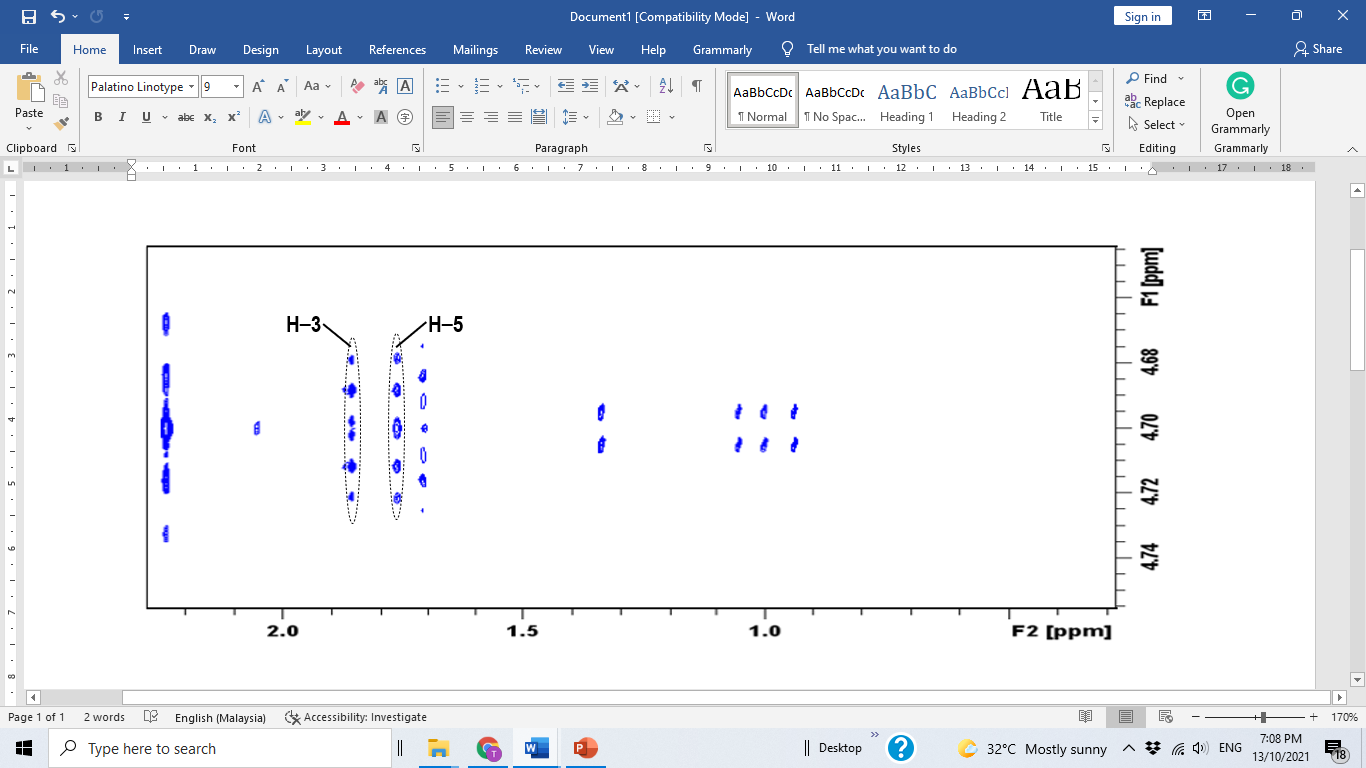


Leucine Lysine


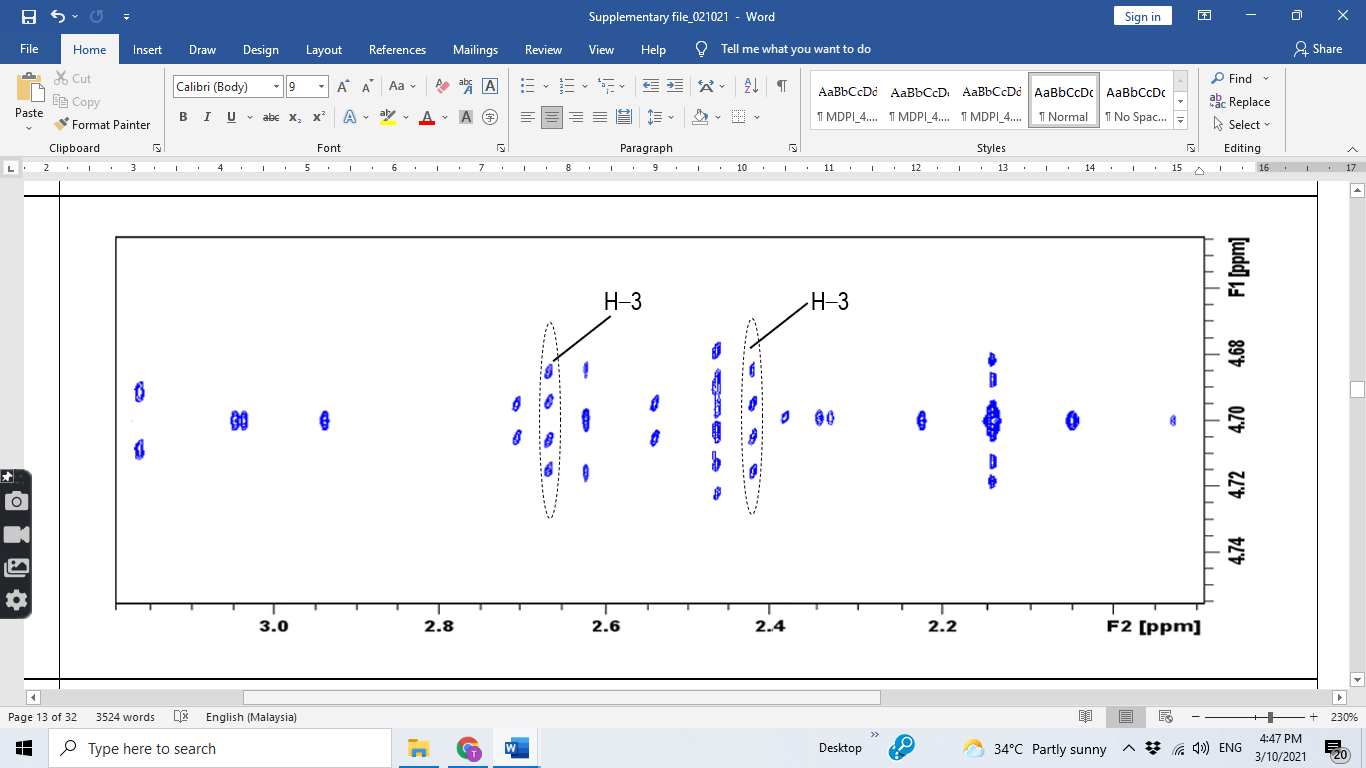

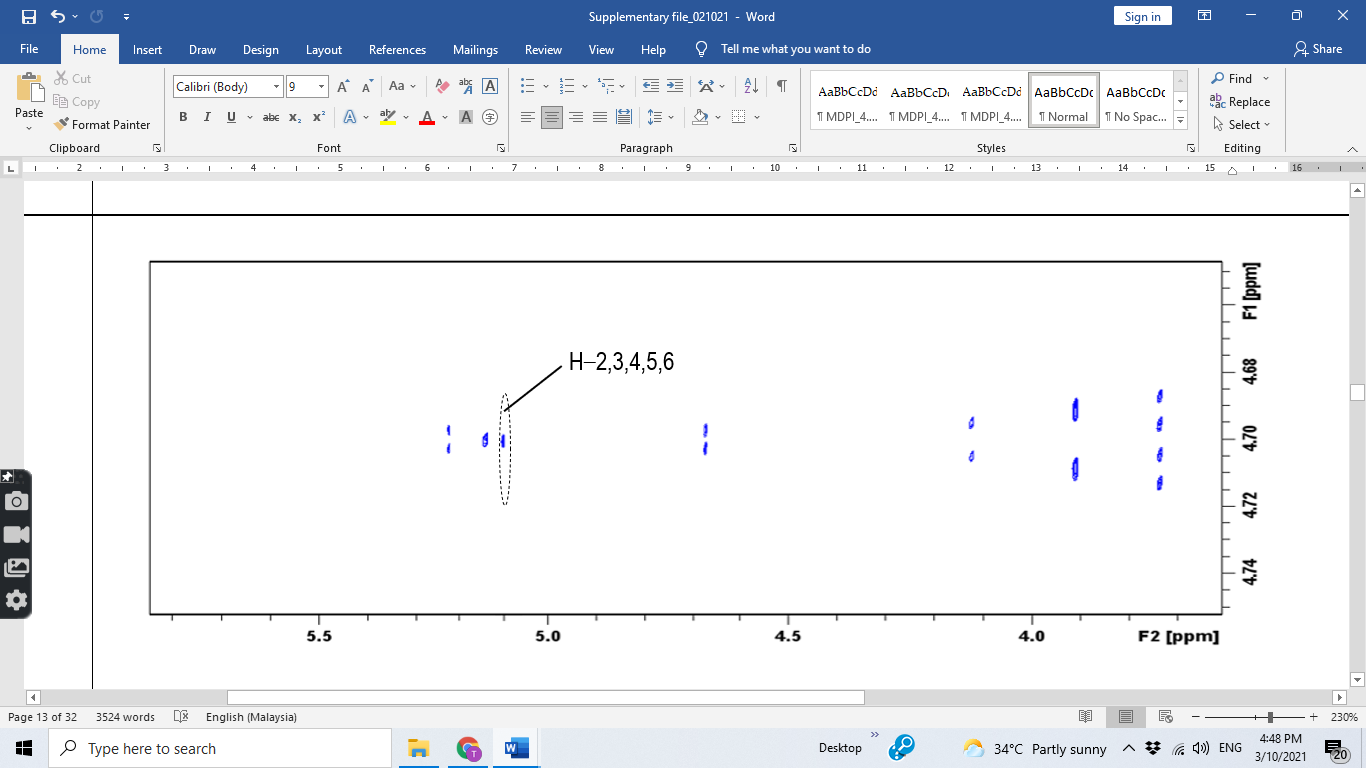


Malate Mannose


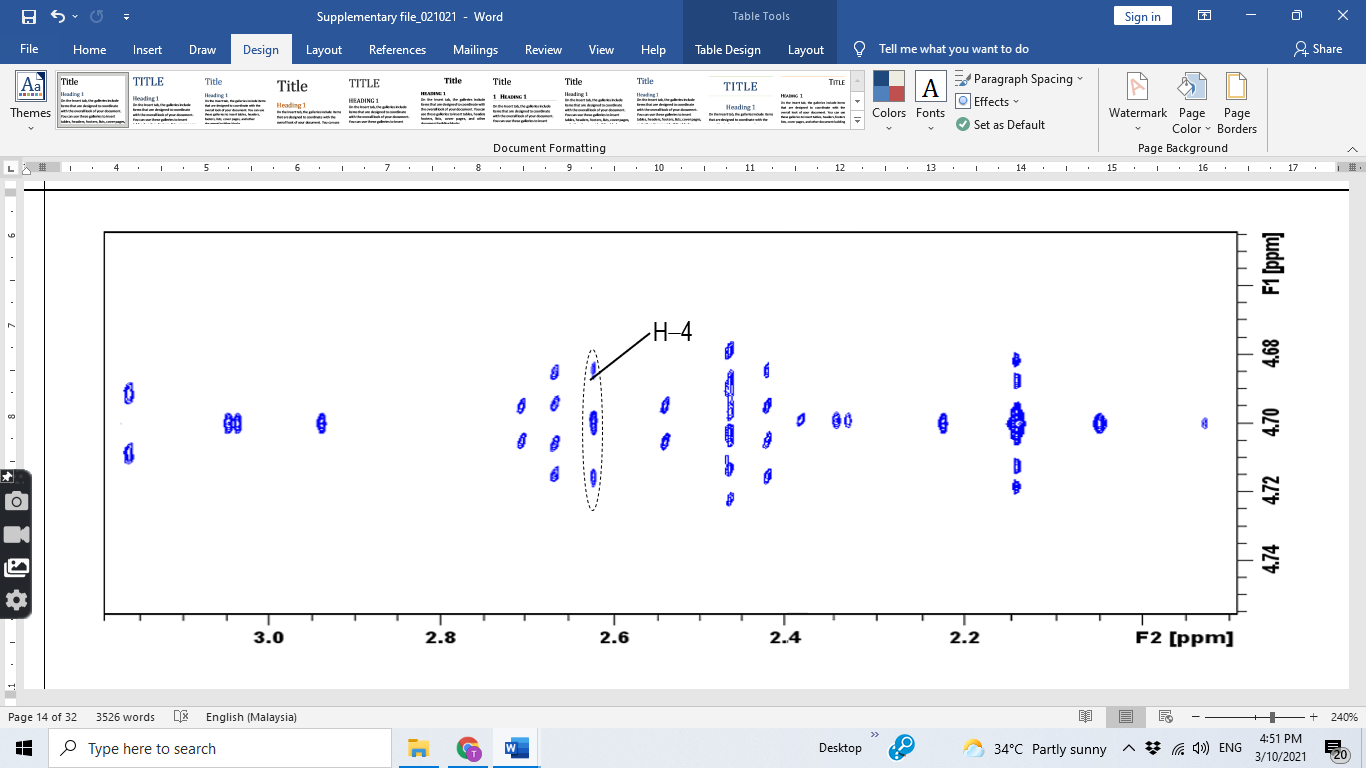

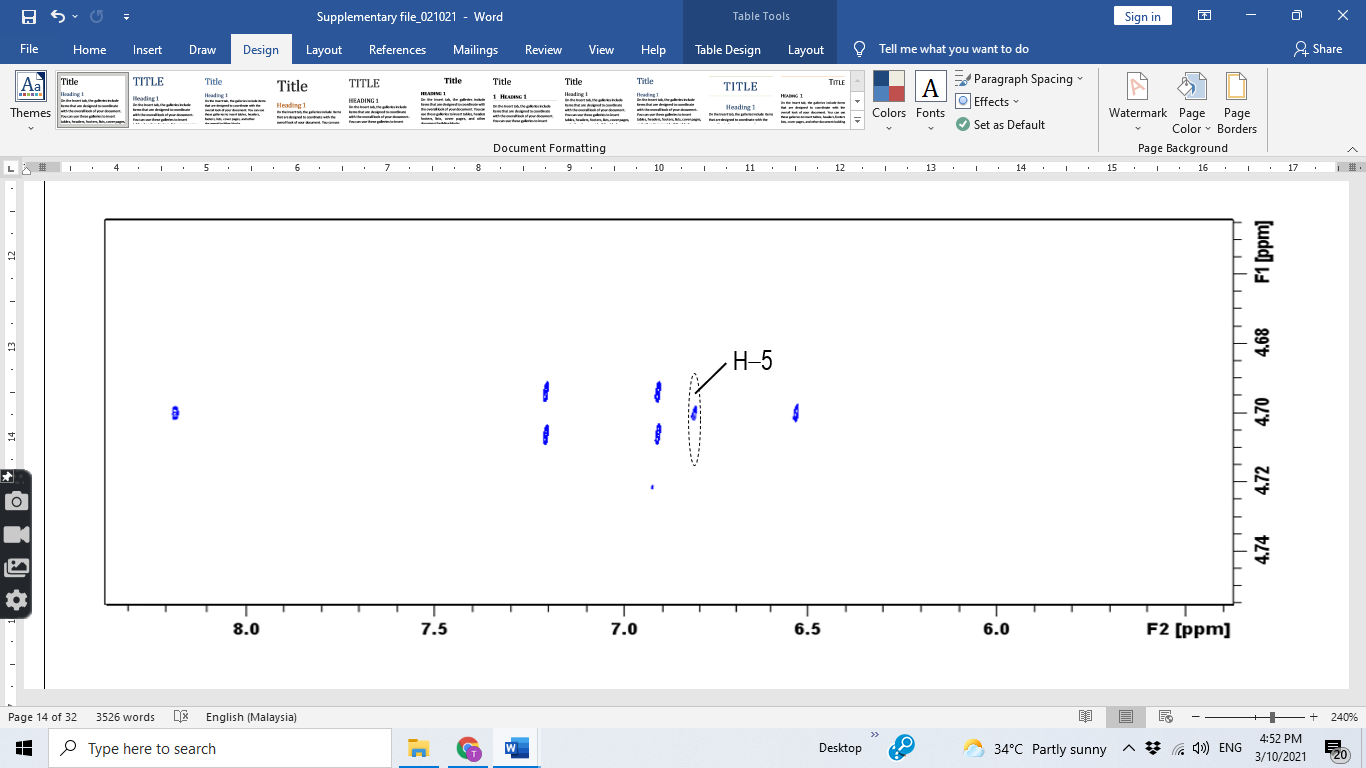


Methionine 3-methylhistidine


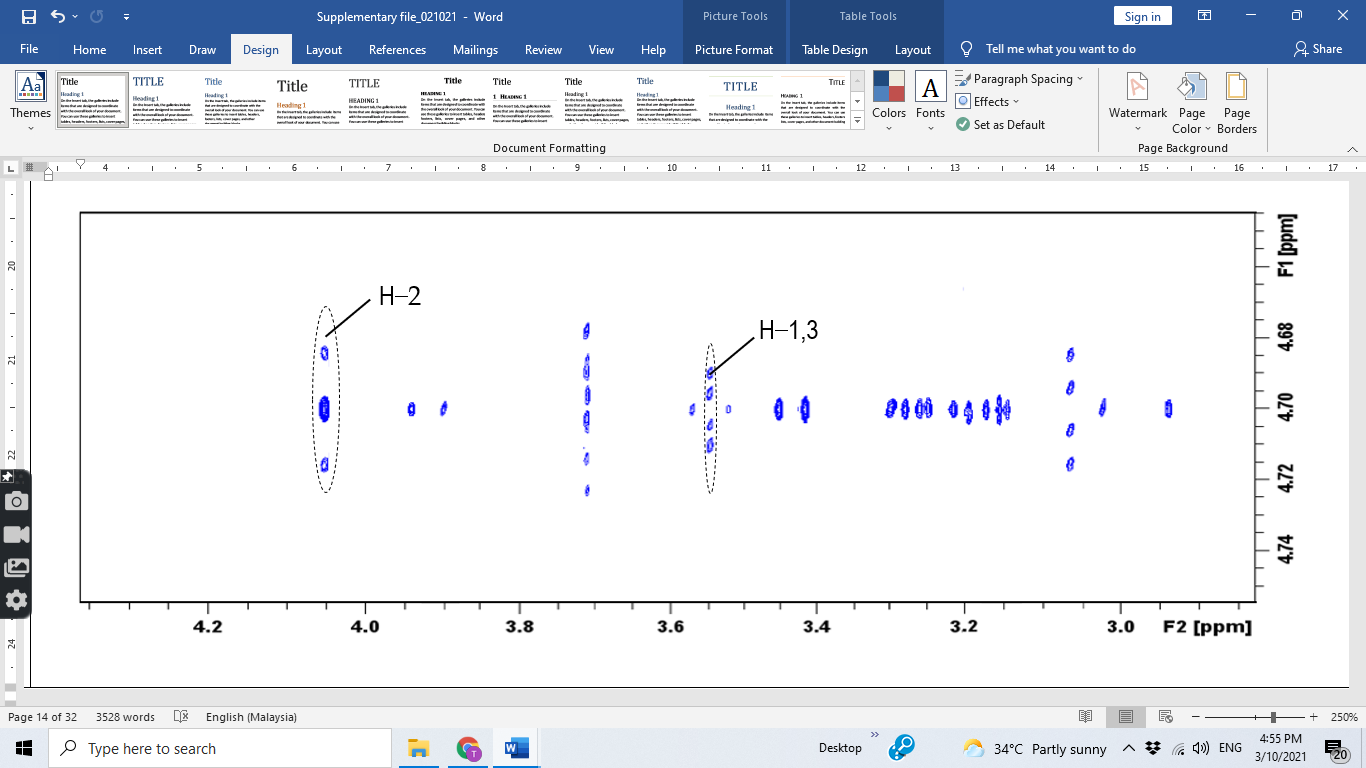

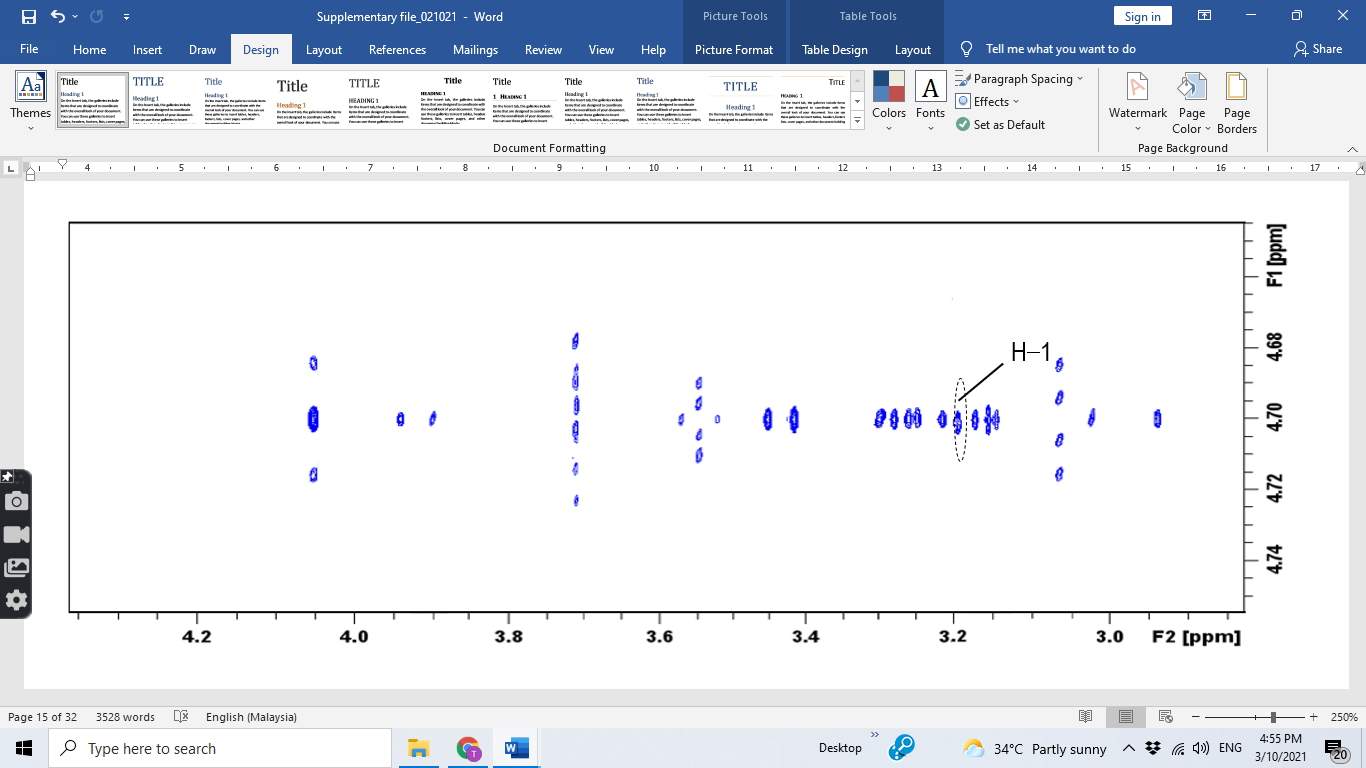


Myo-inositol O-phosphocholine


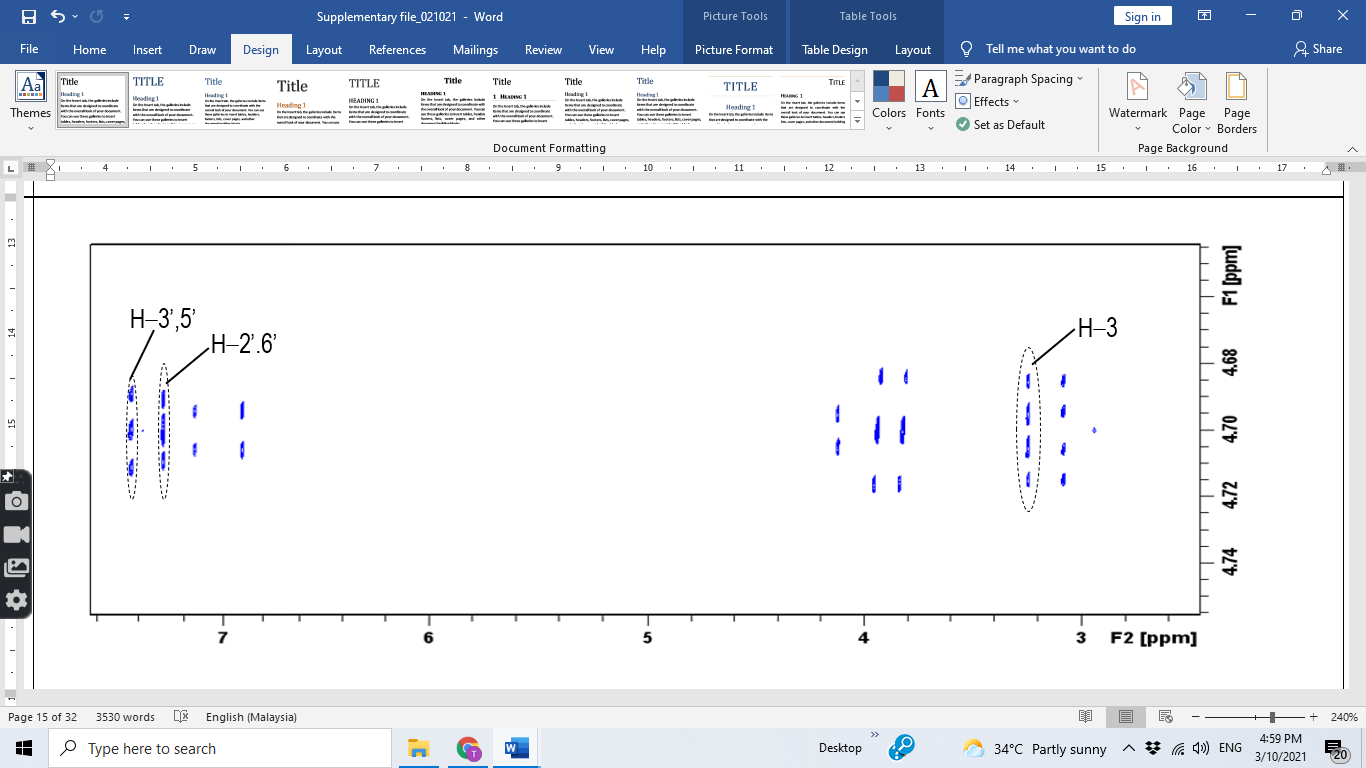

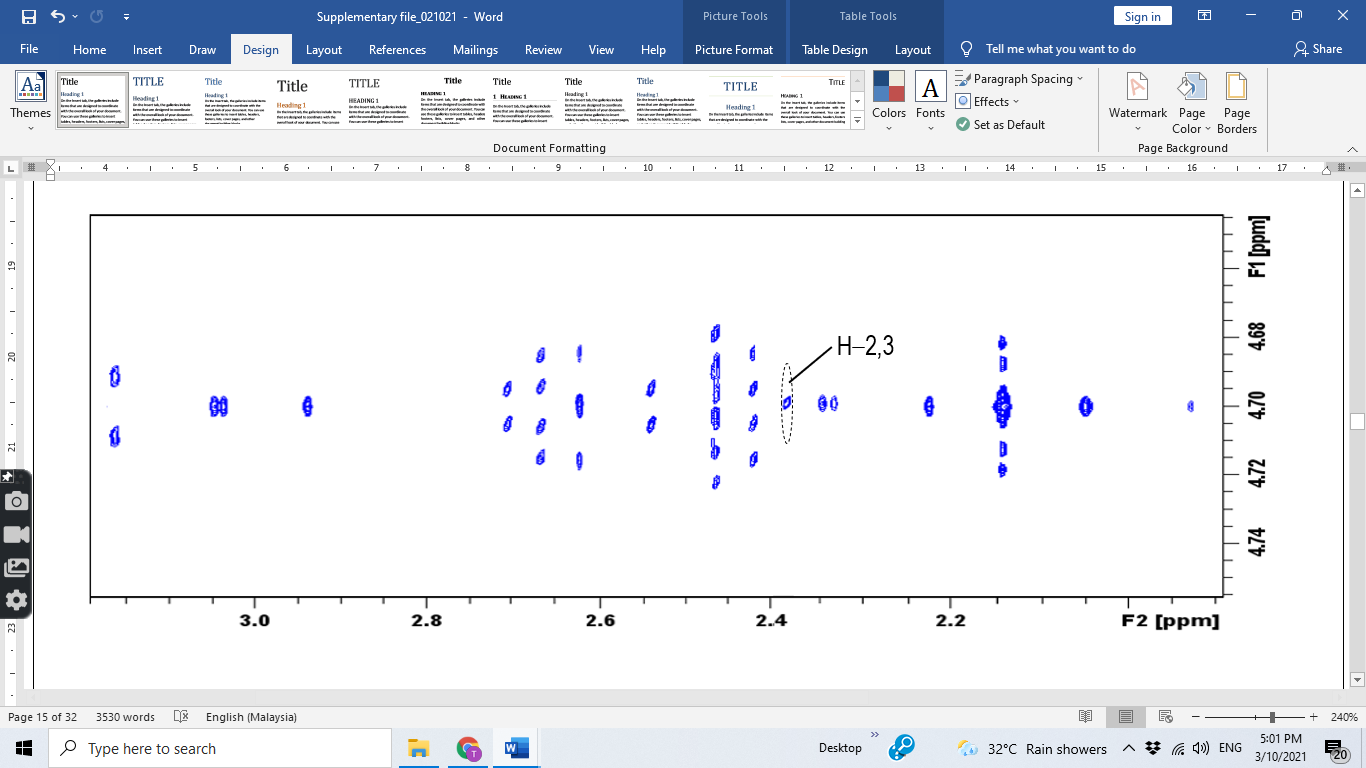


Phenylalanine Succinate


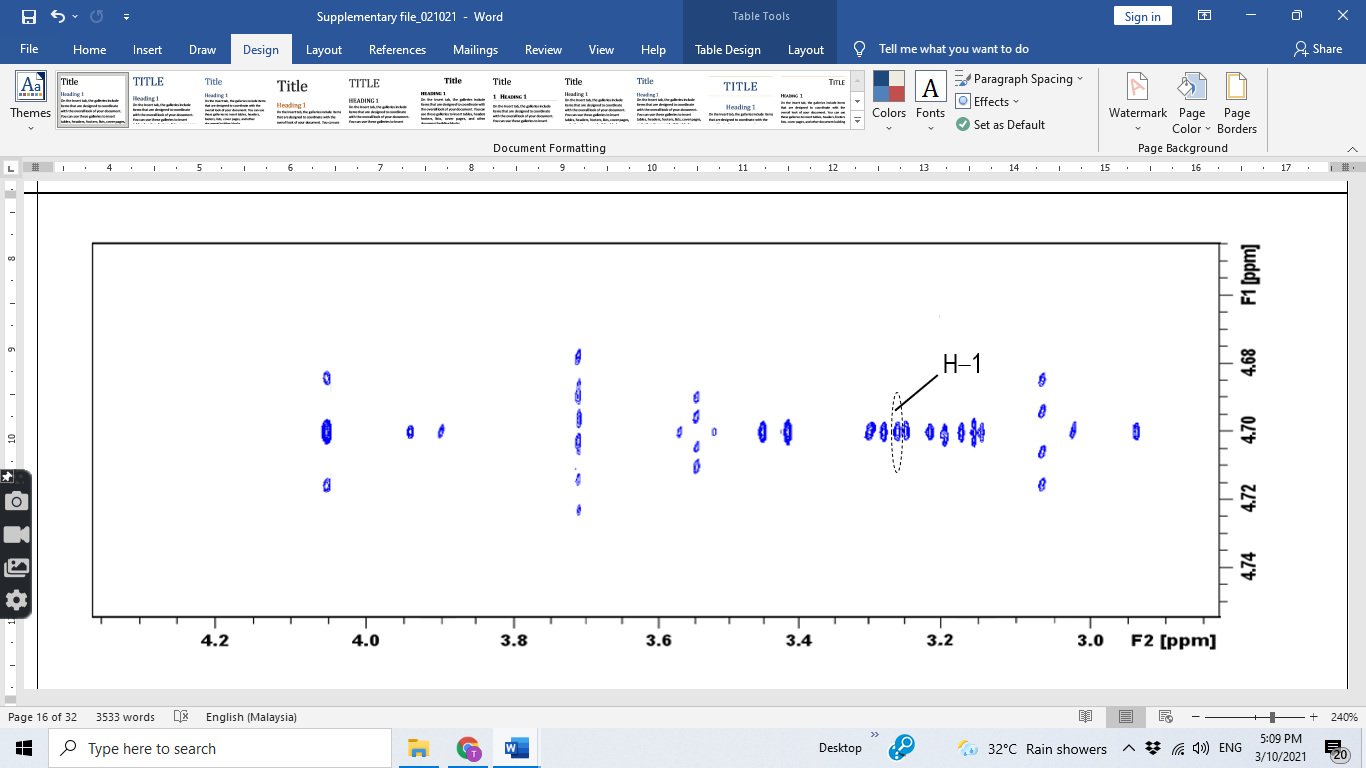

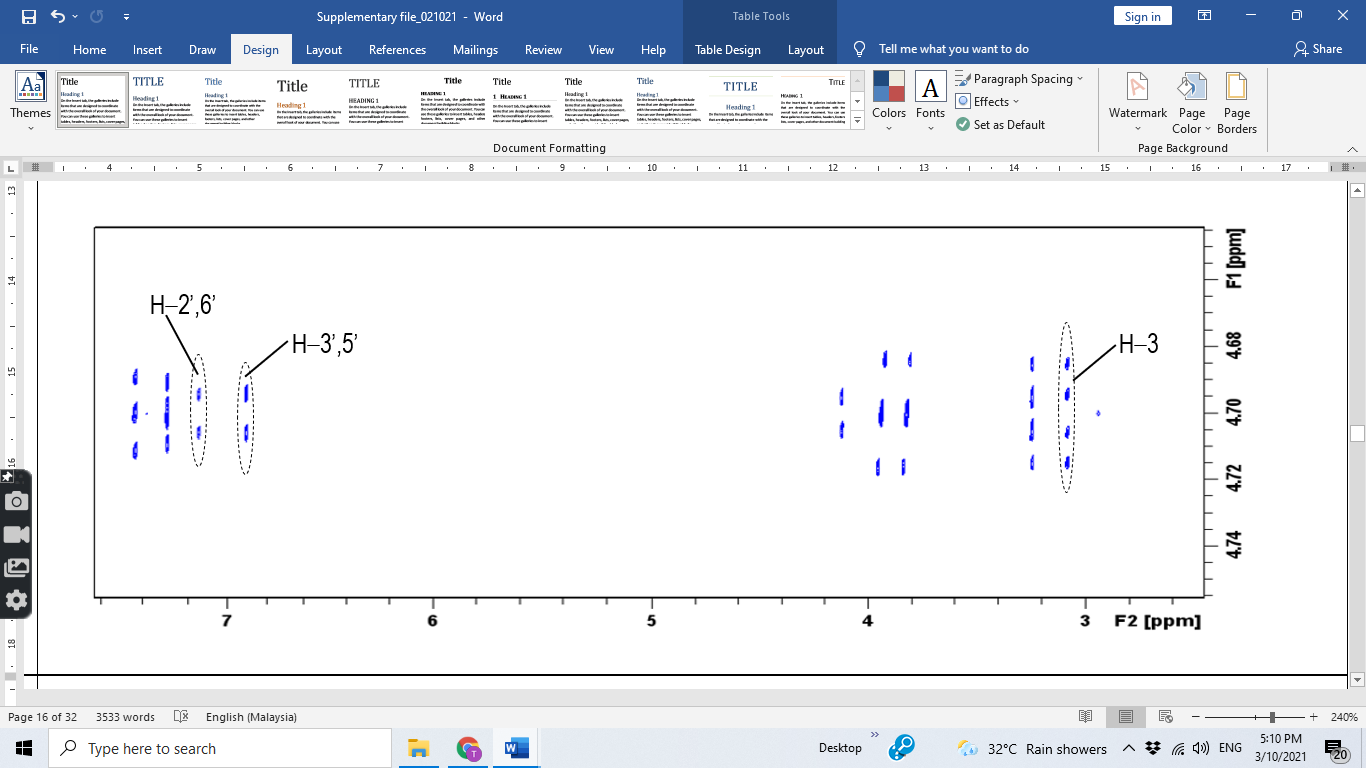


Trimethylamine N-oxide Tyrosine


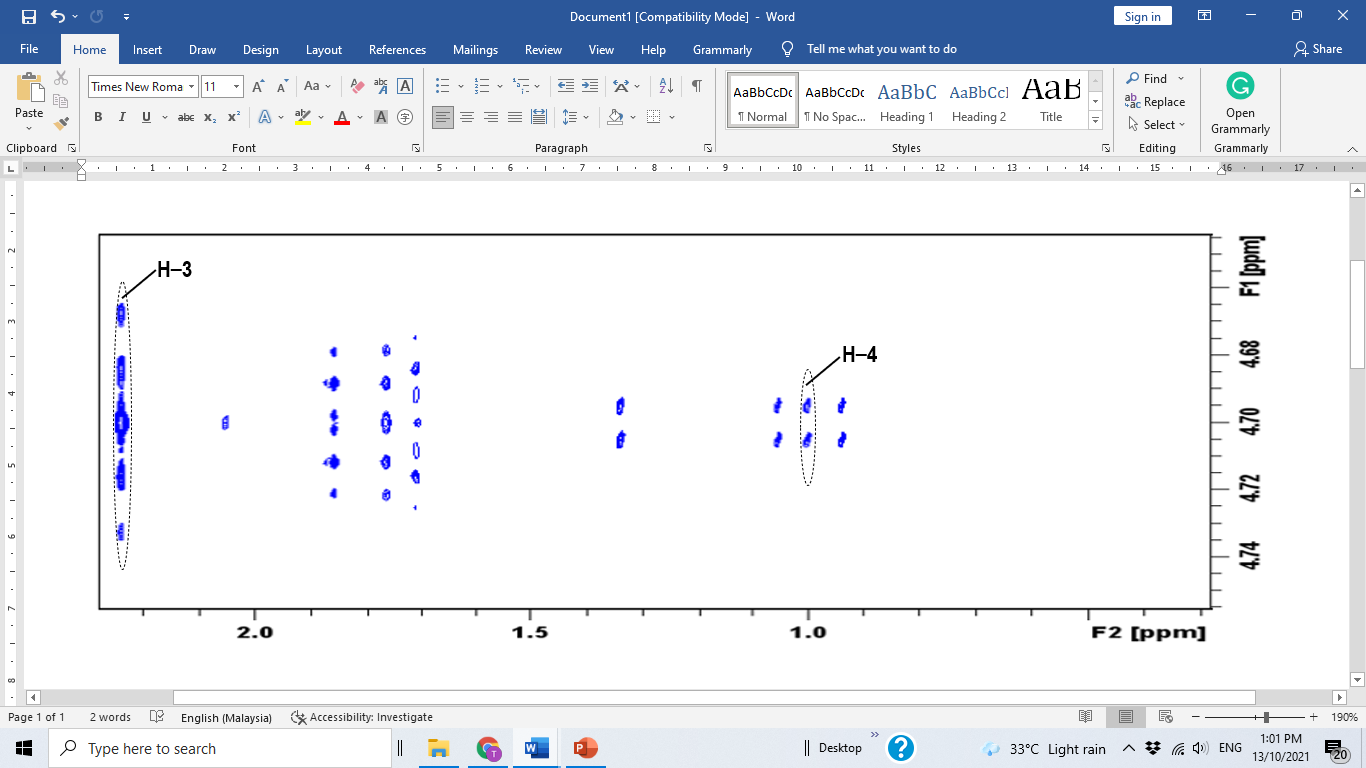

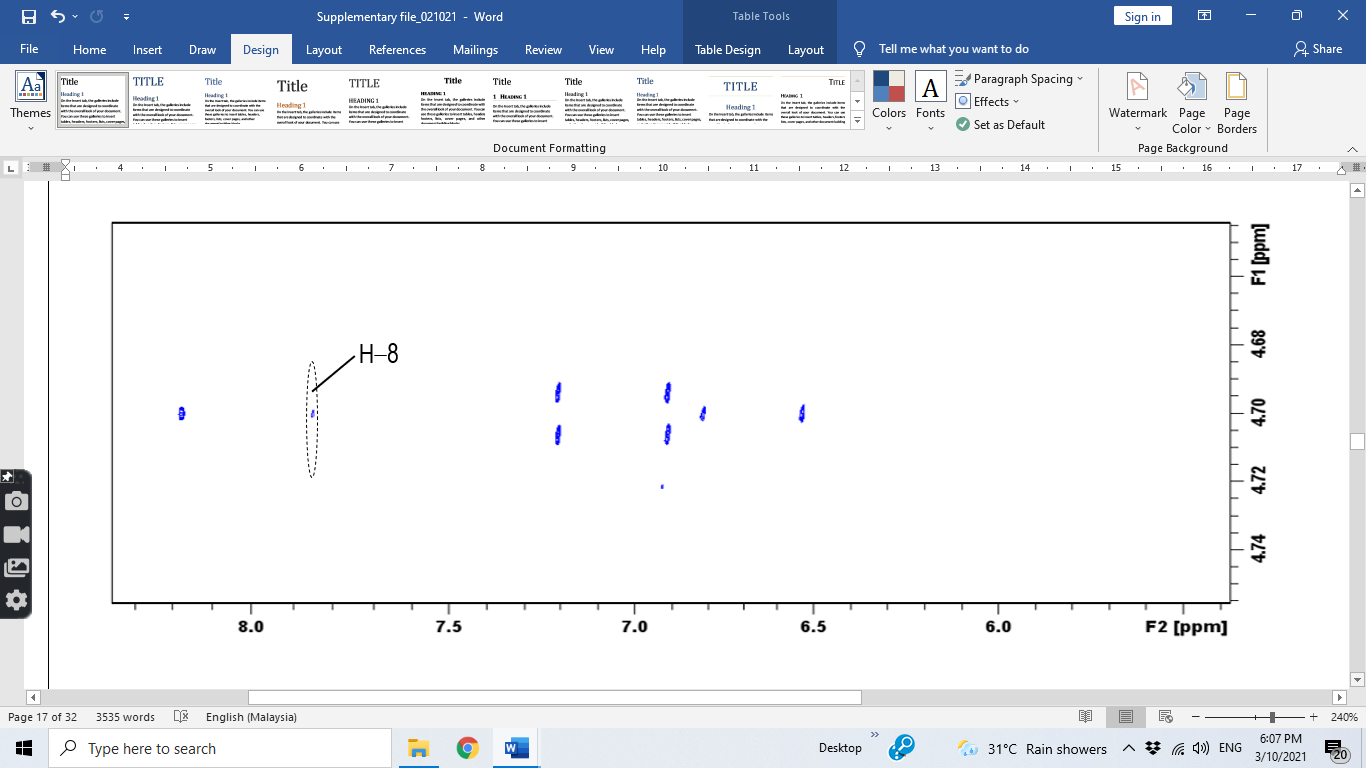


Valine Xanthine

**FIGURE** **S4 (**Continued)


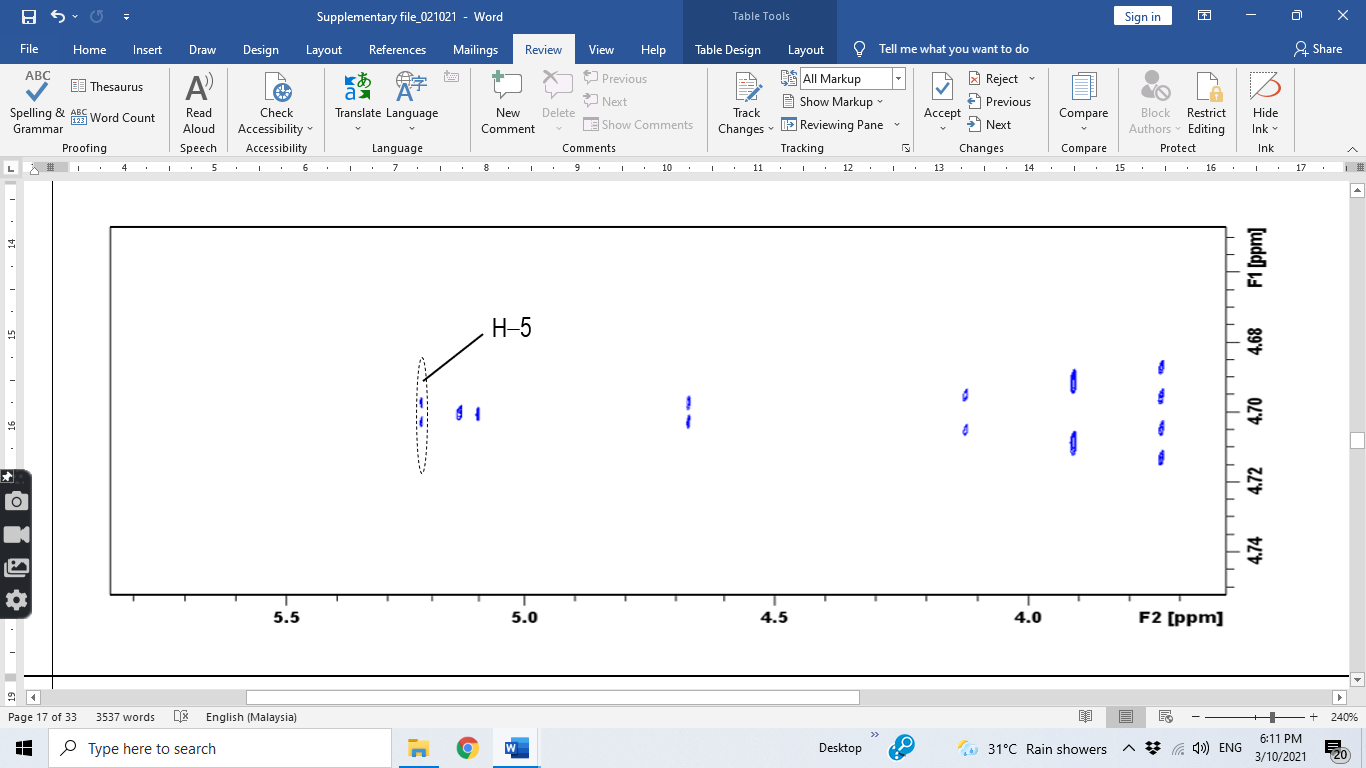

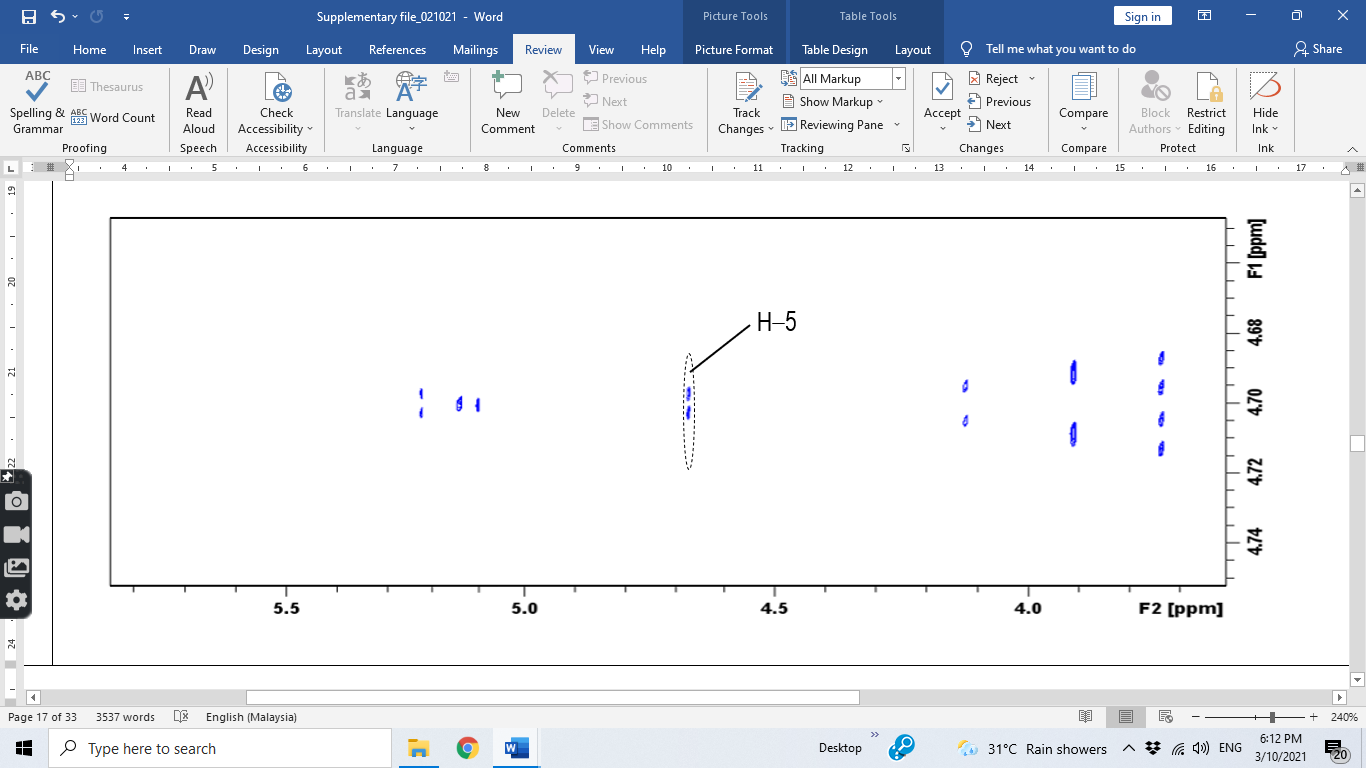


α−glucose β−glucose

**FIGURE S4** 700 MHz 2D J-resolved NMR spectra for metabolites identified in chicken serum


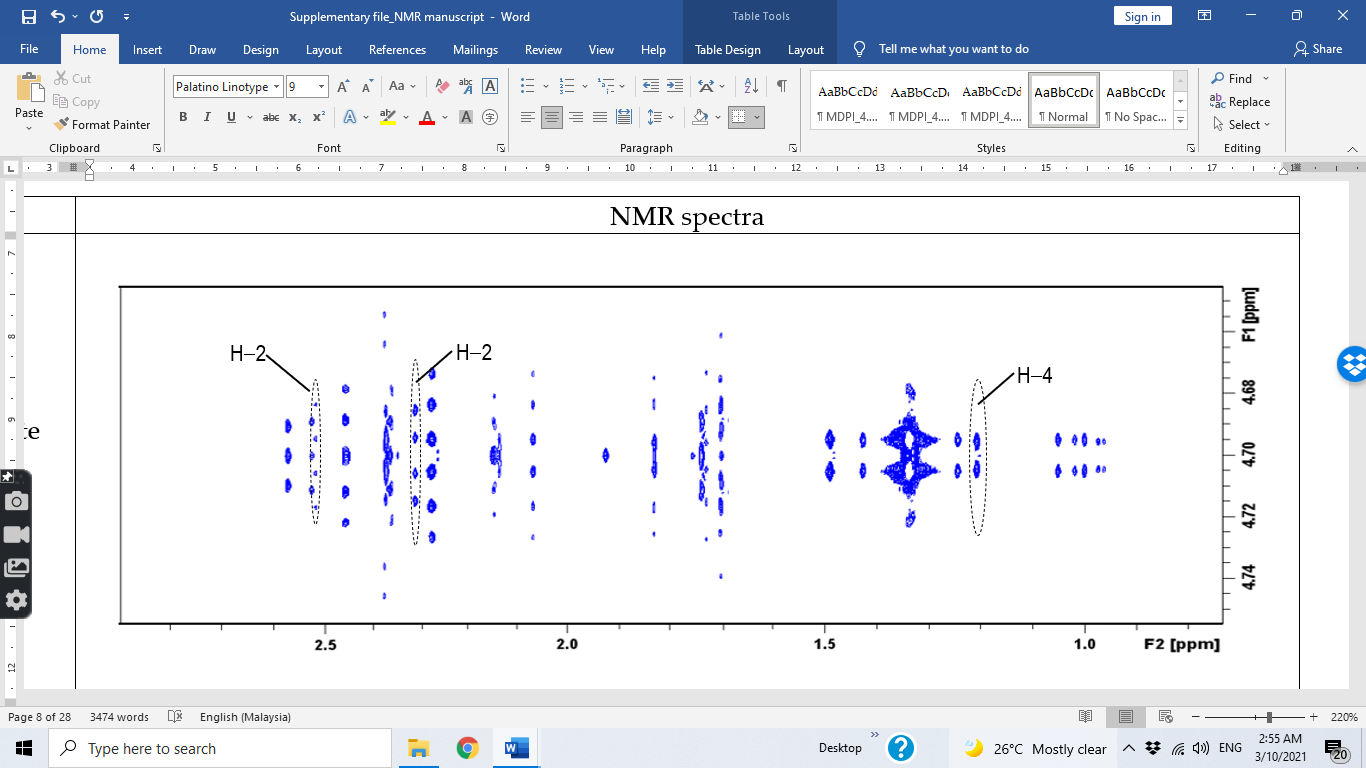

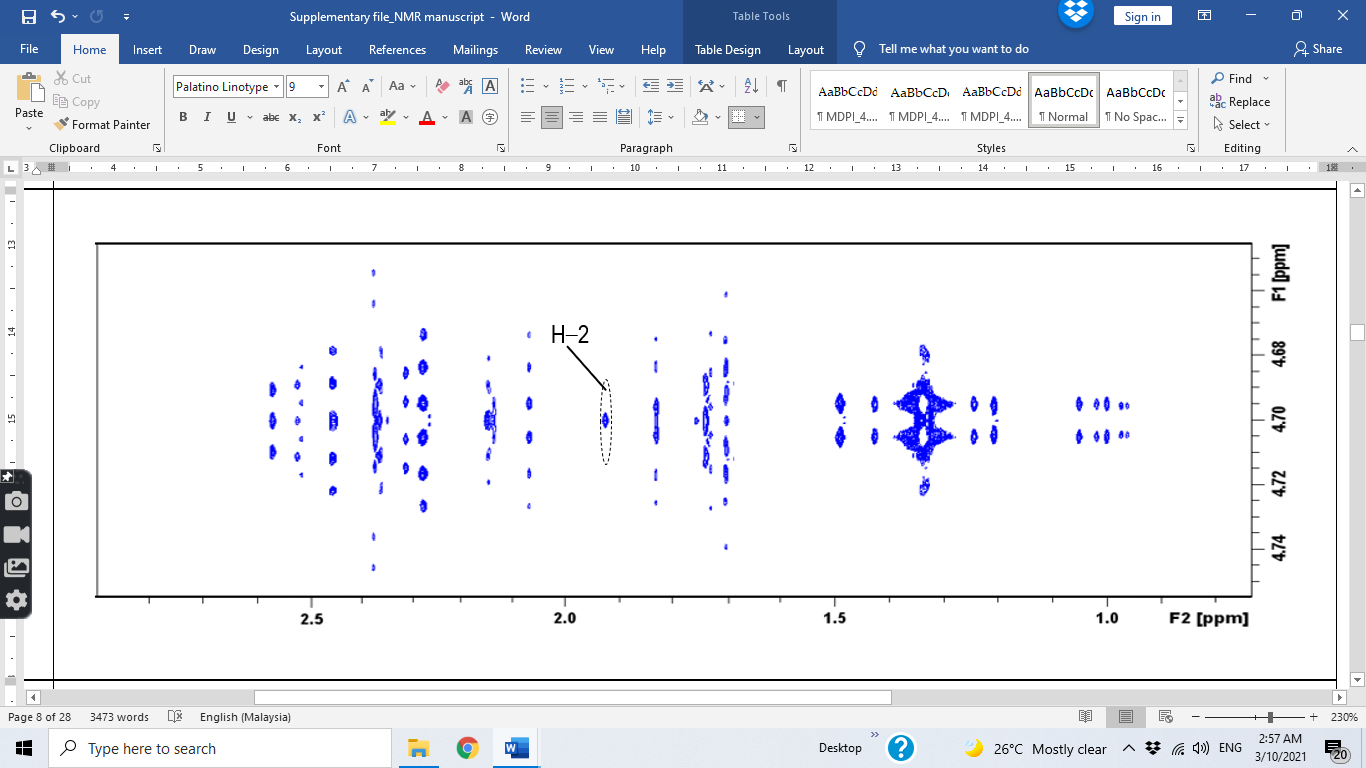


3-hydroxybutyrate Acetate


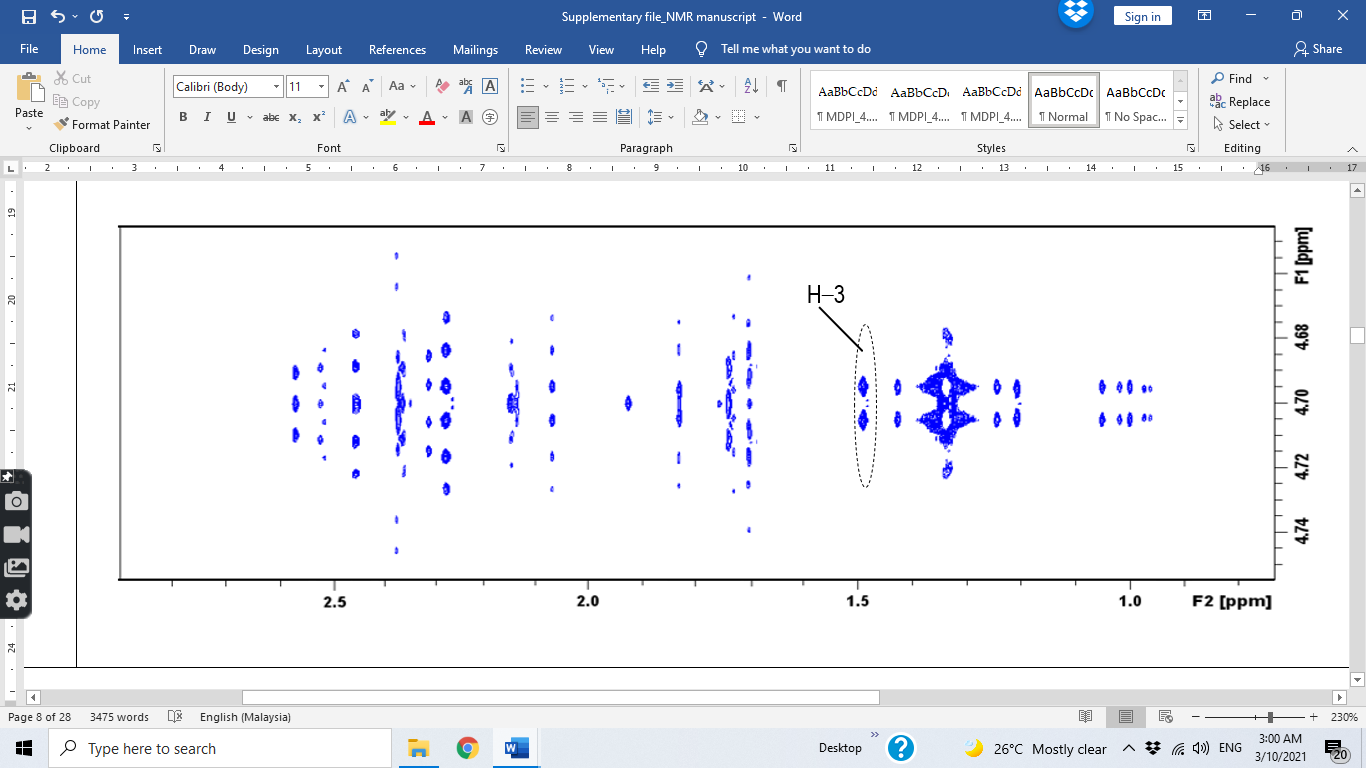

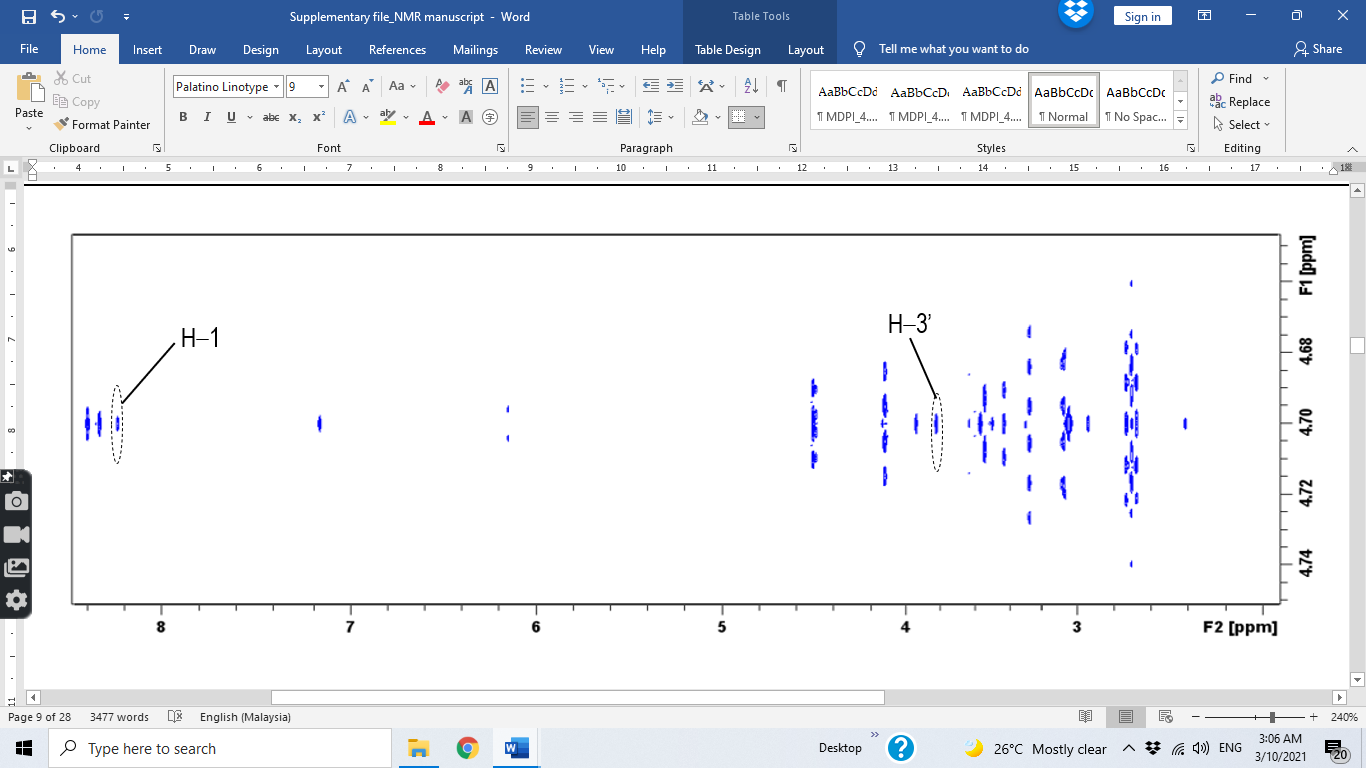


Alanine Anserine


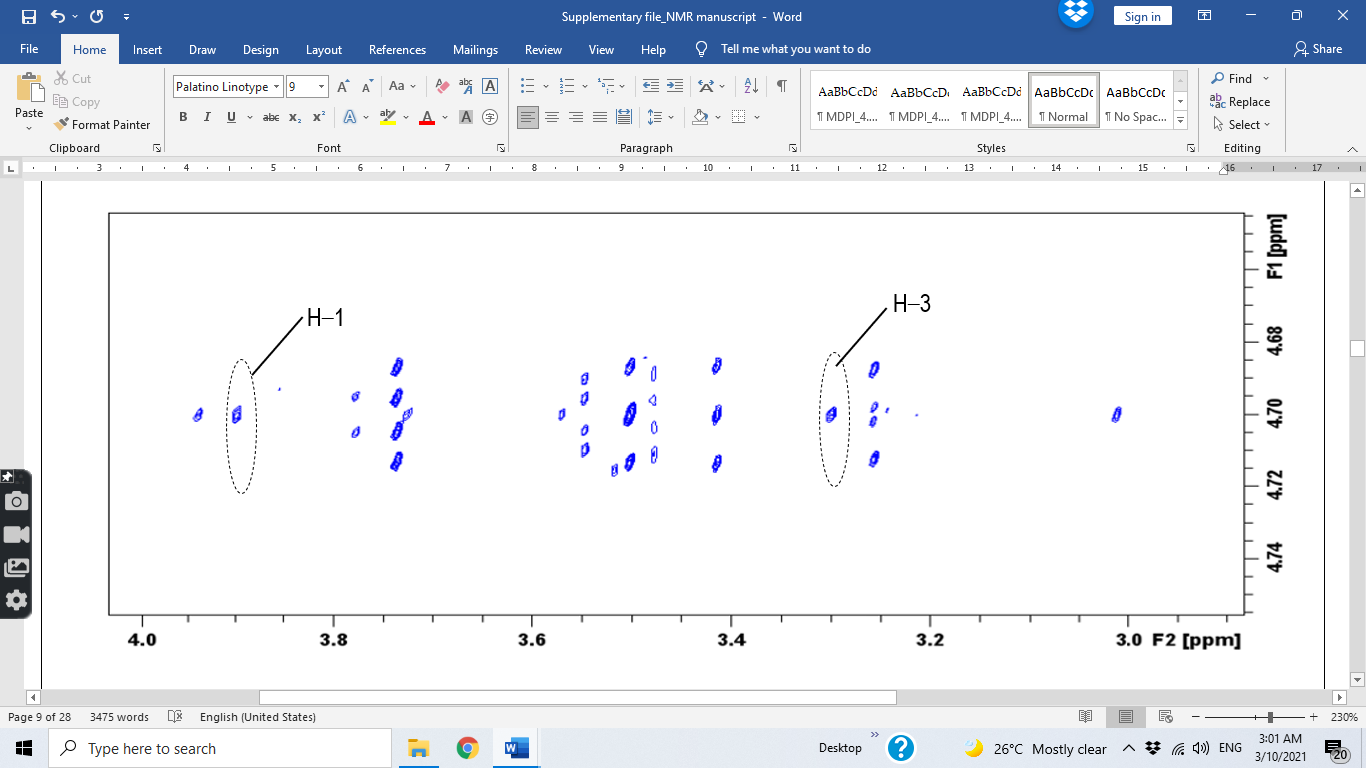

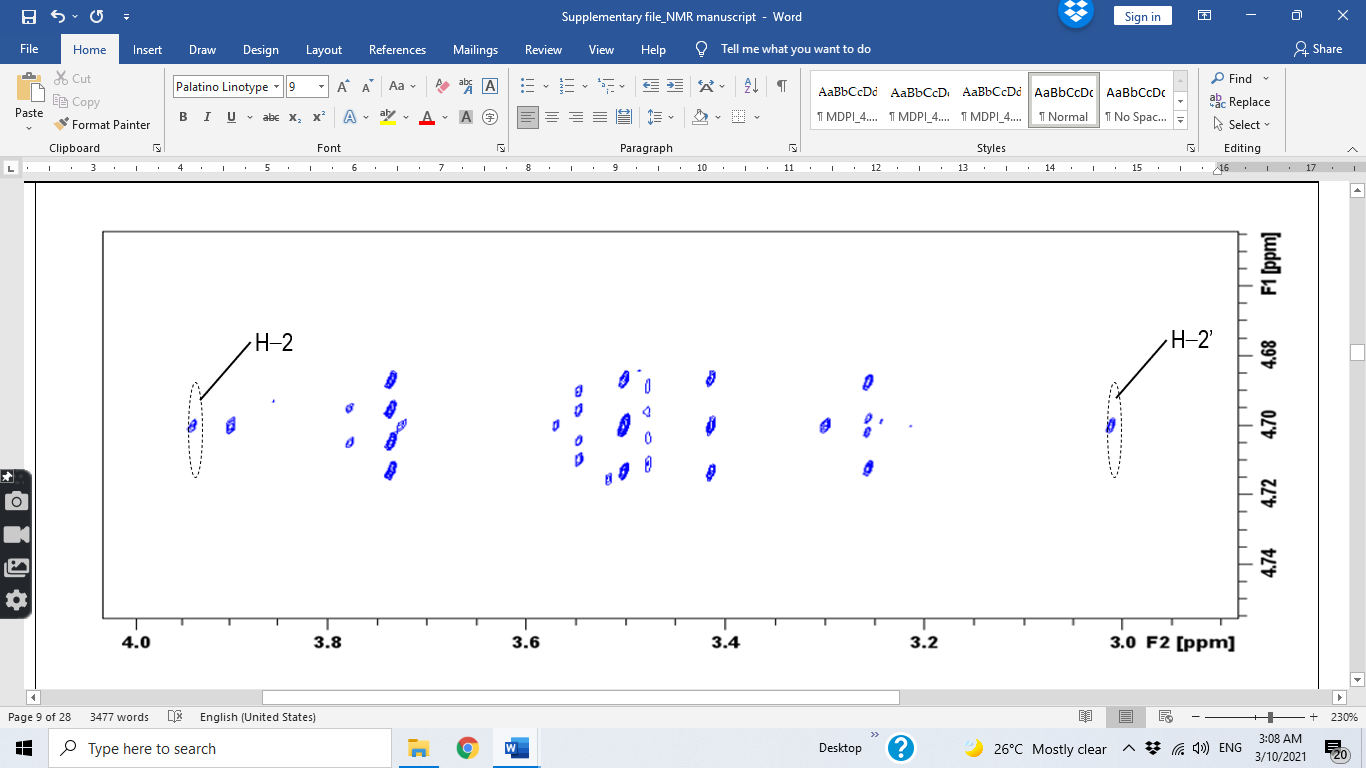


Betaine Creatine


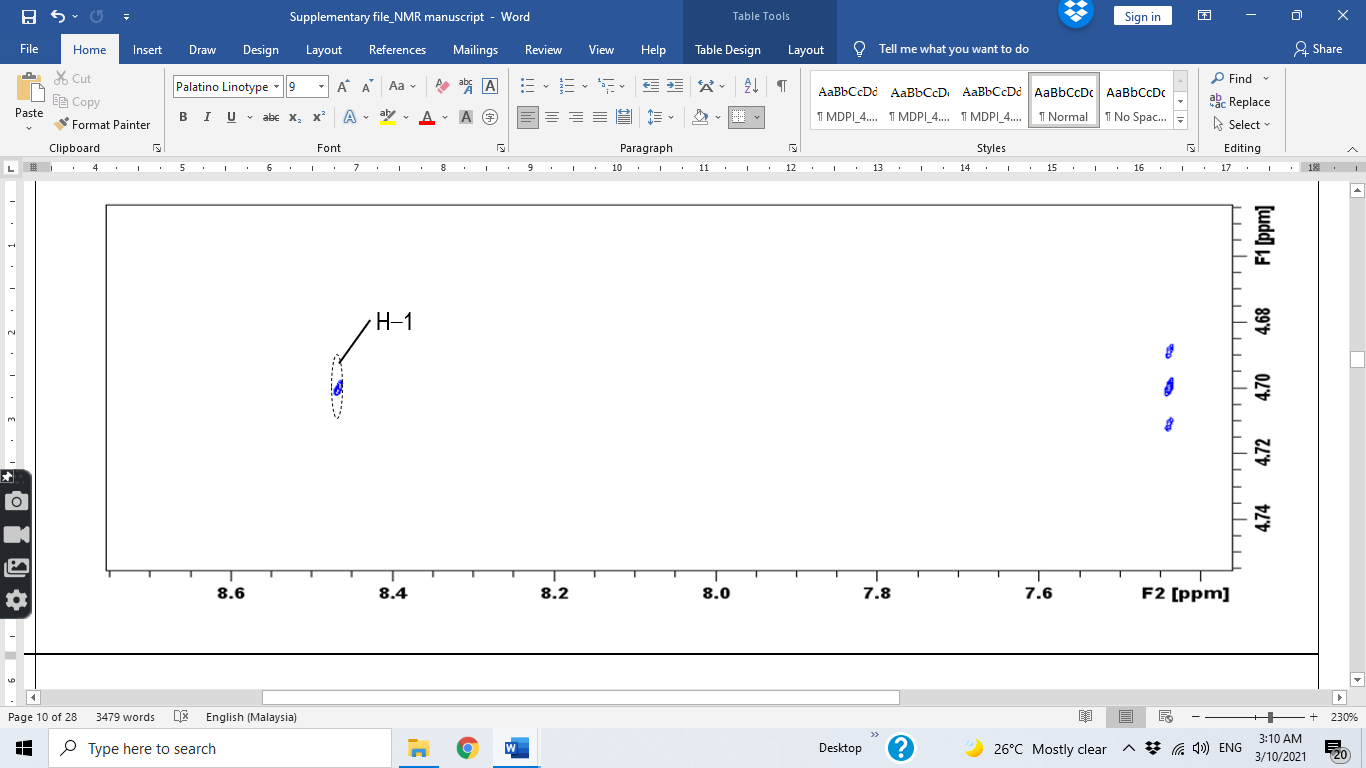

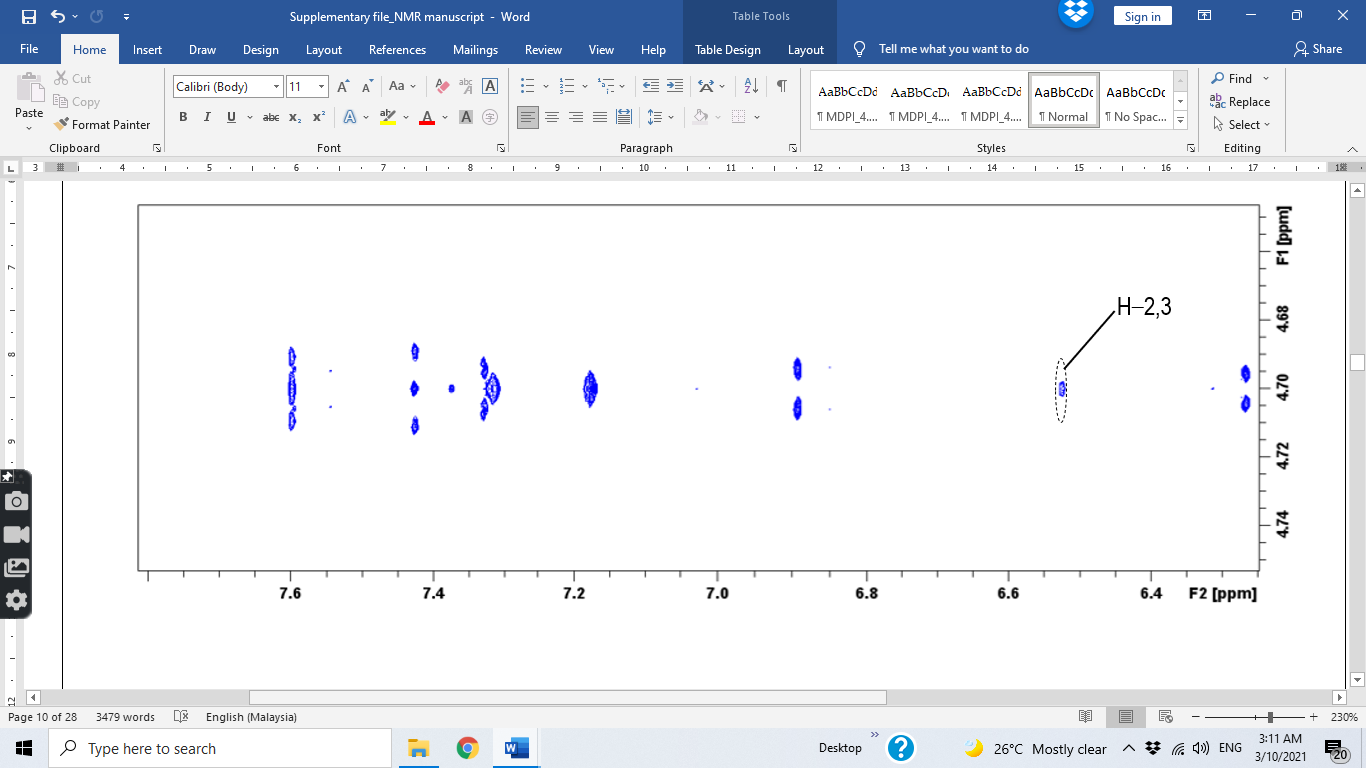


Formate Fumarate


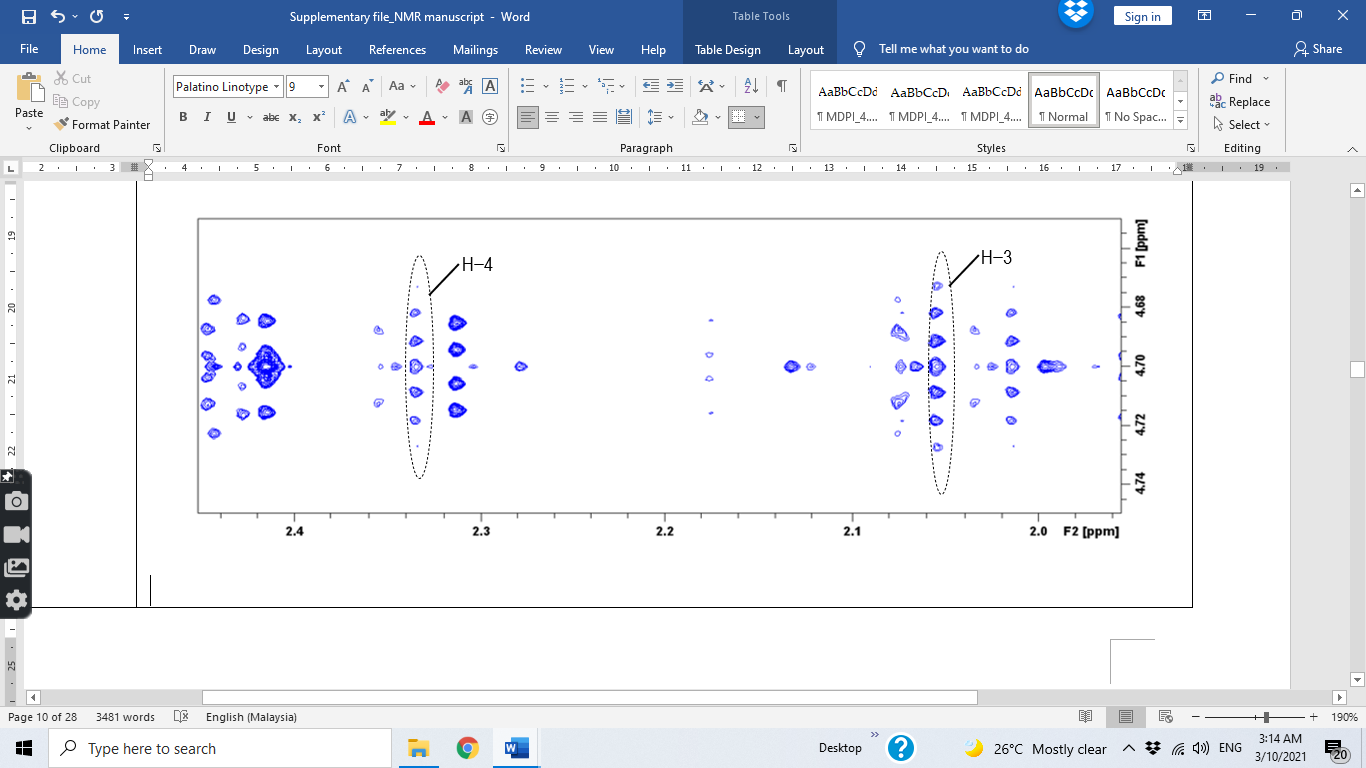

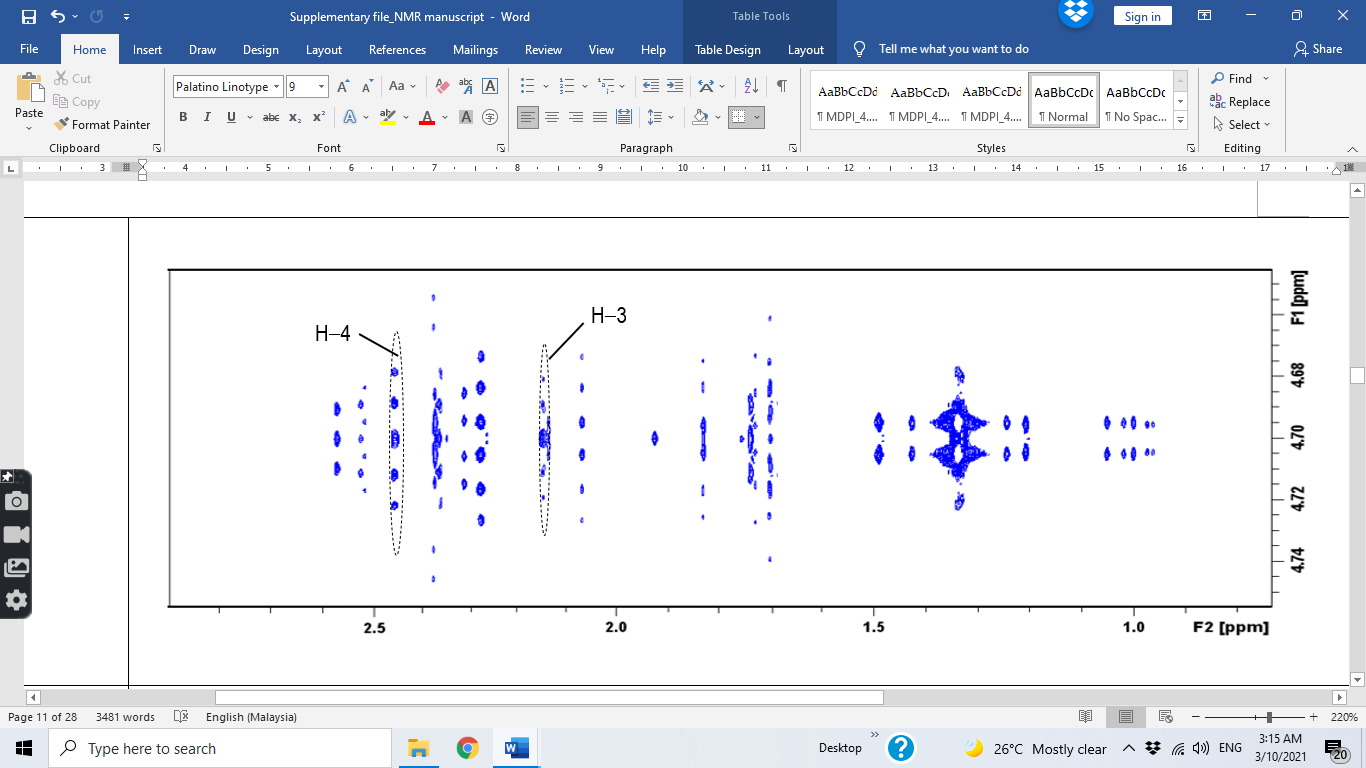


Glutamate Glutamine


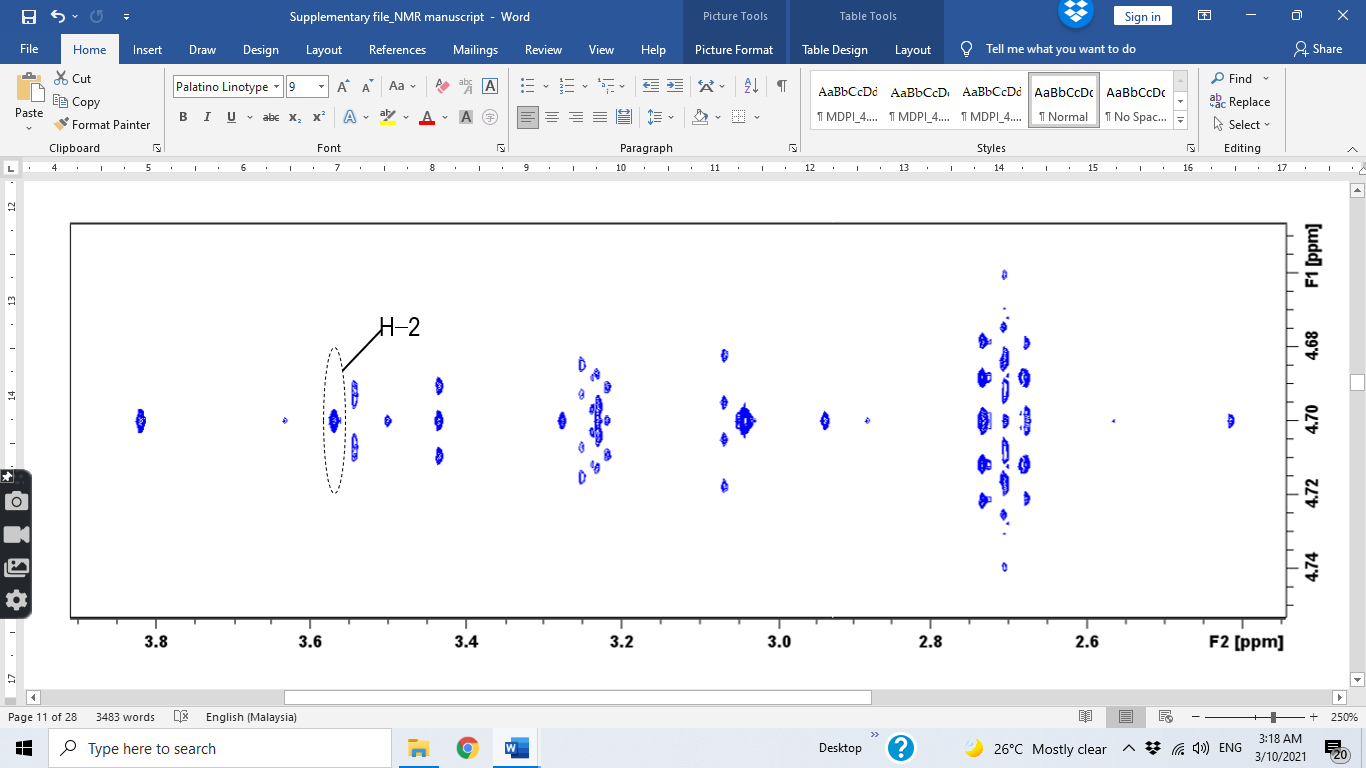

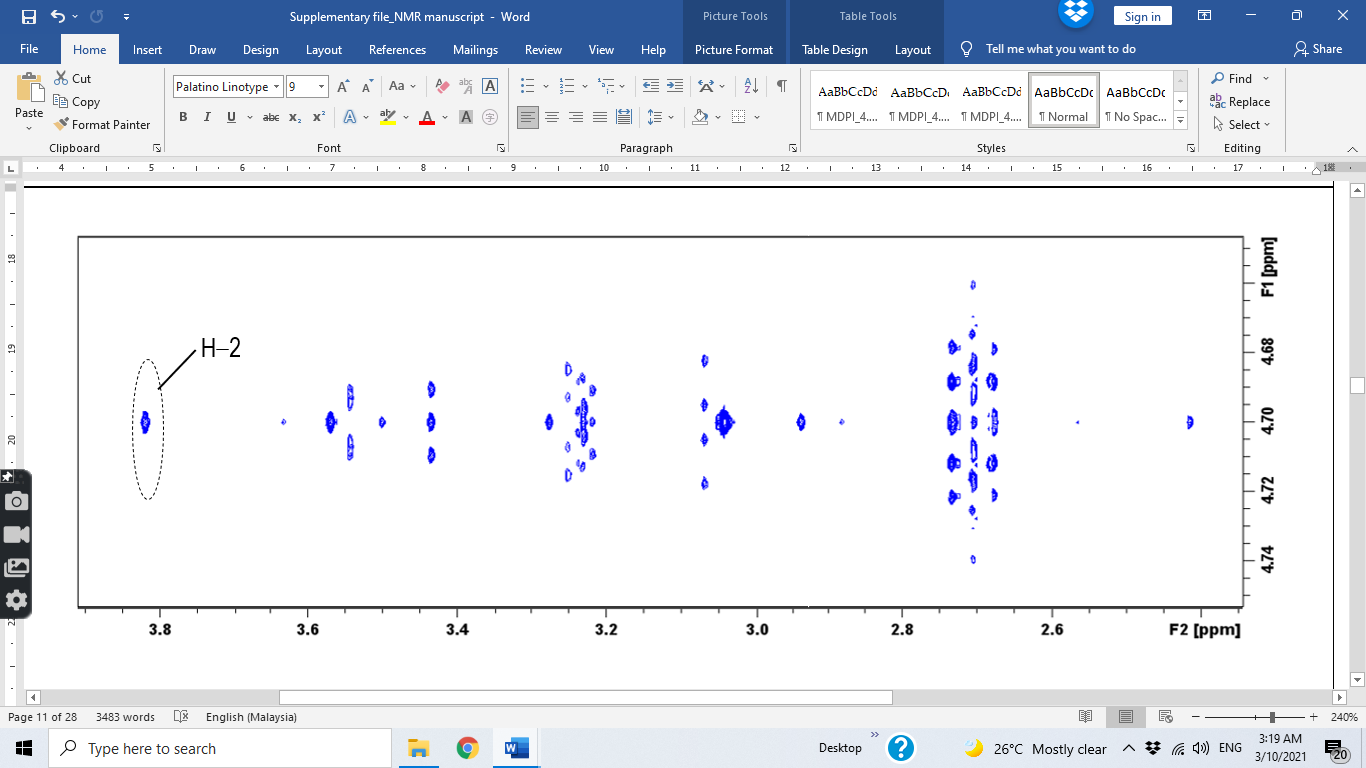


Glycine Guanidoacetate


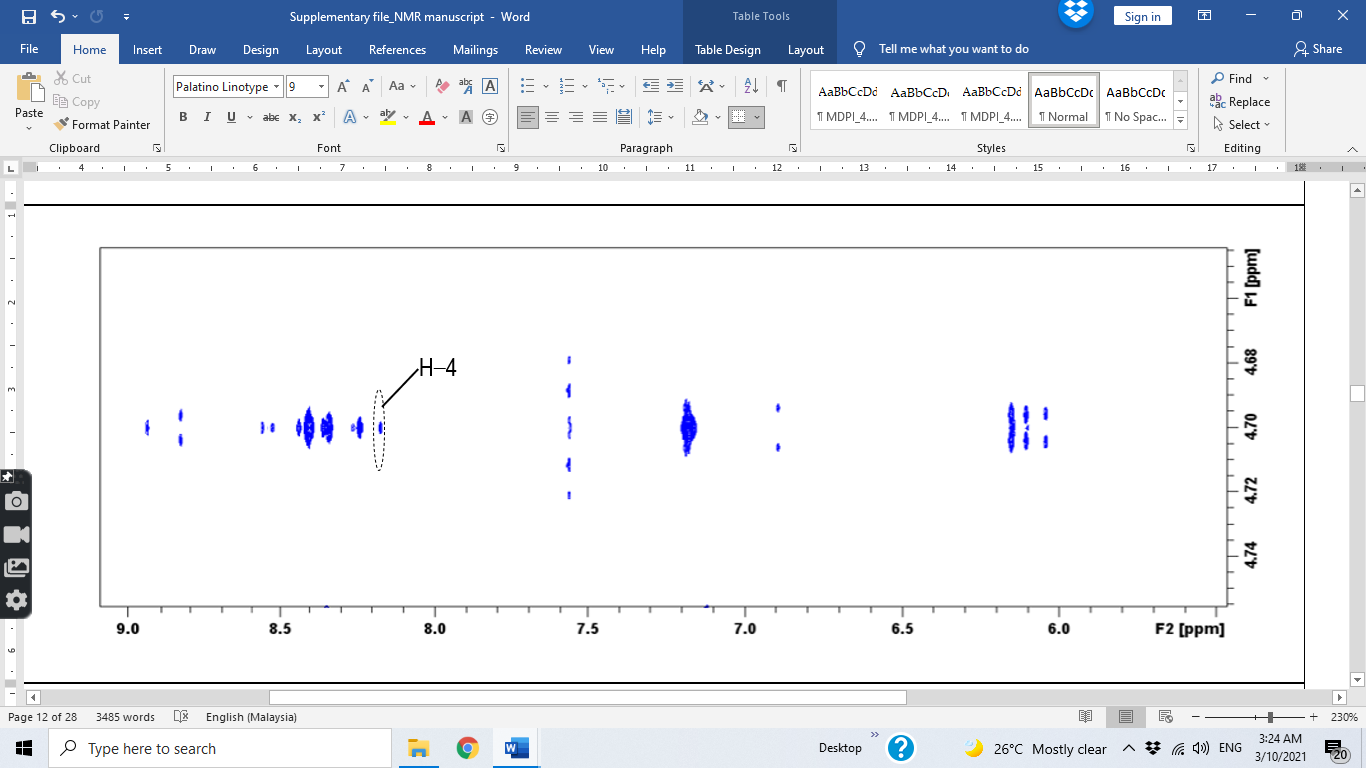

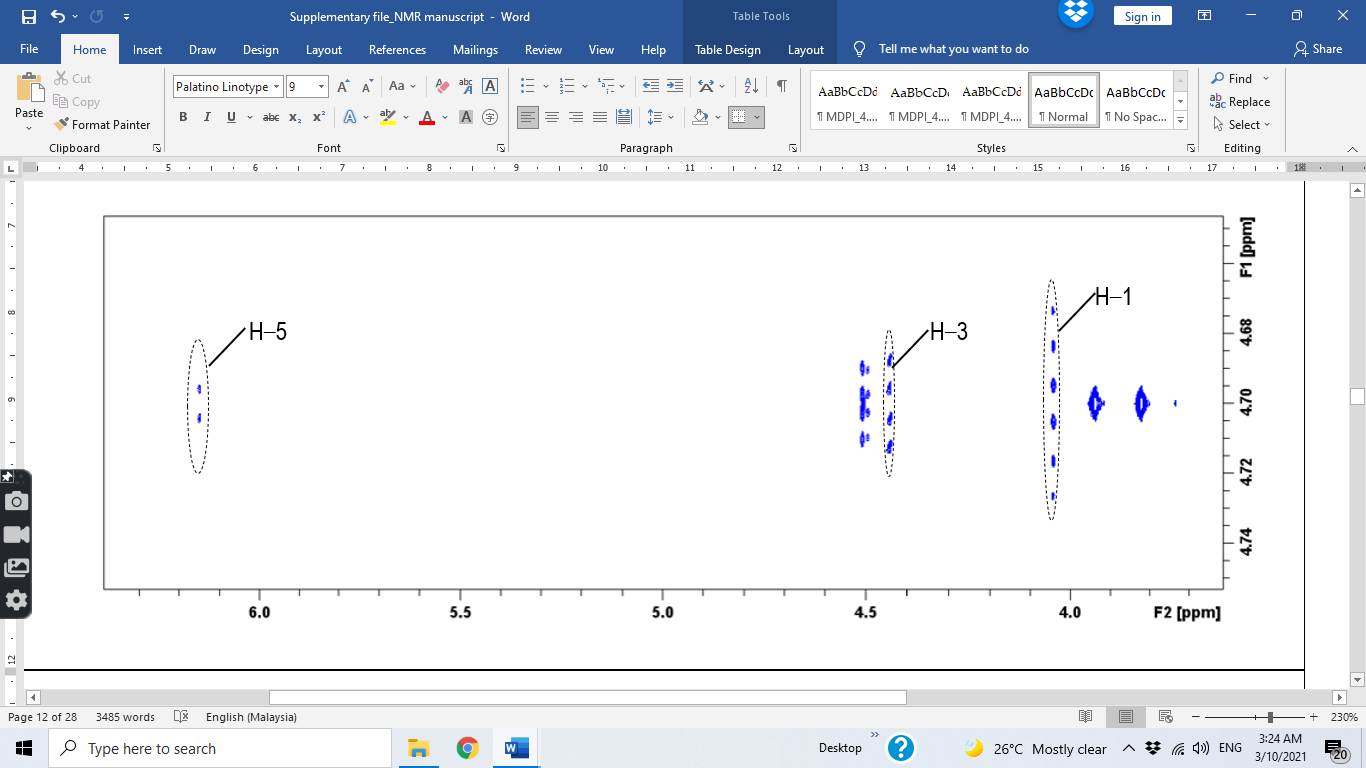


Hypoxanthine IMP

**FIGURE** **S5 (**Continued)


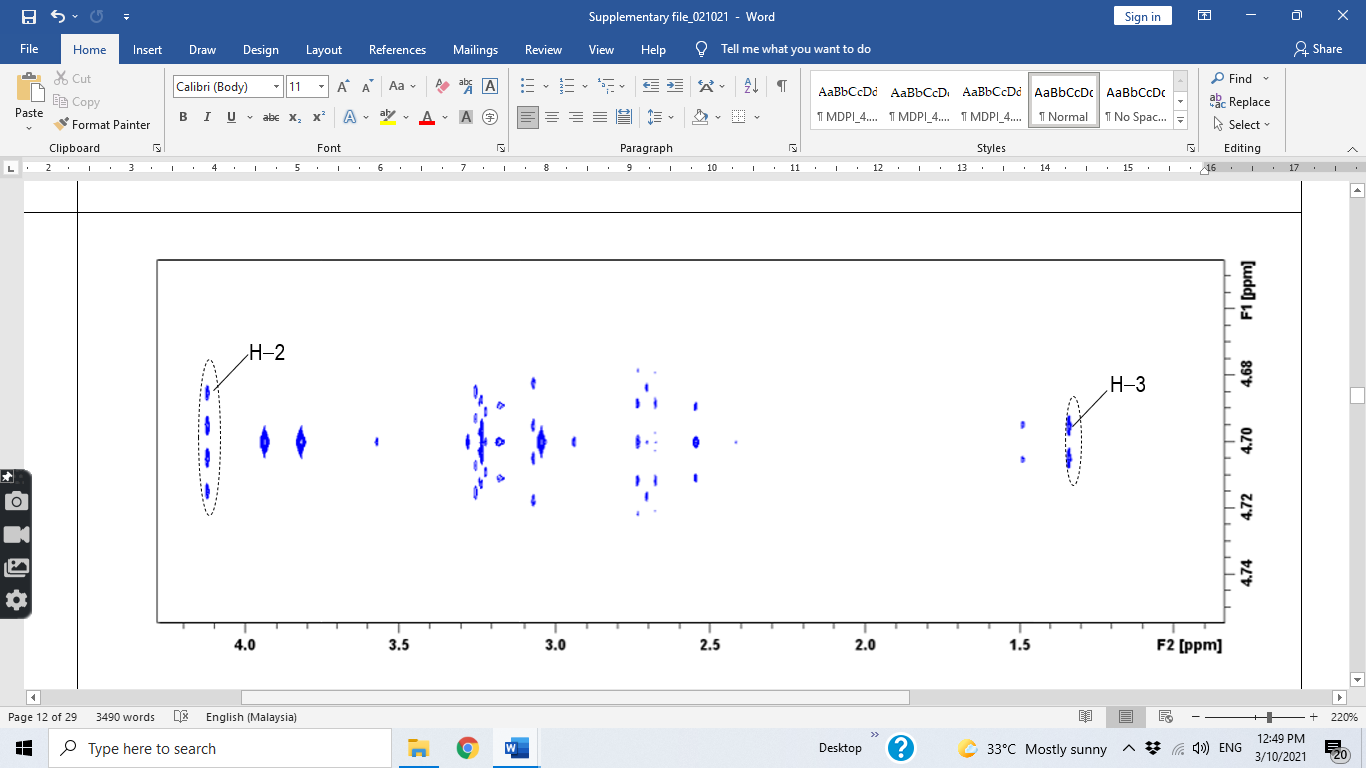

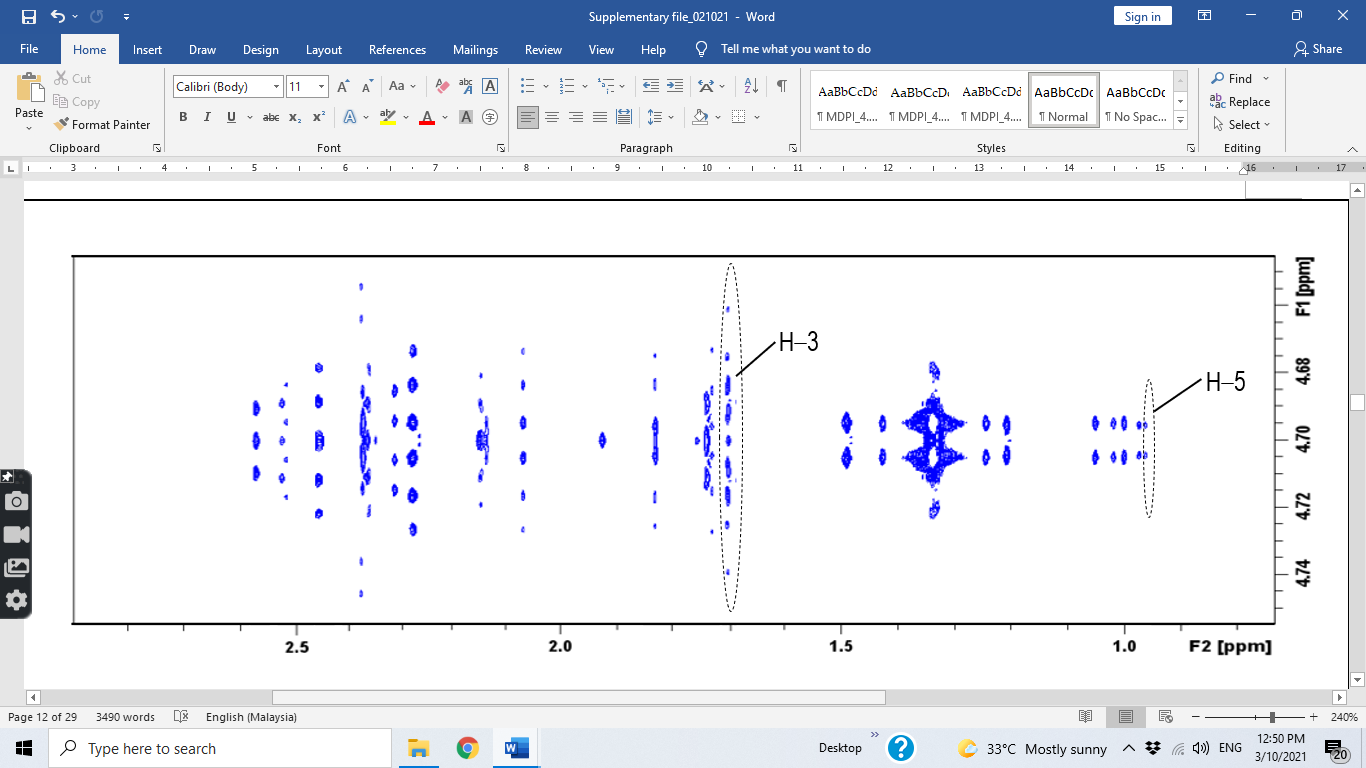


Lactate leucine


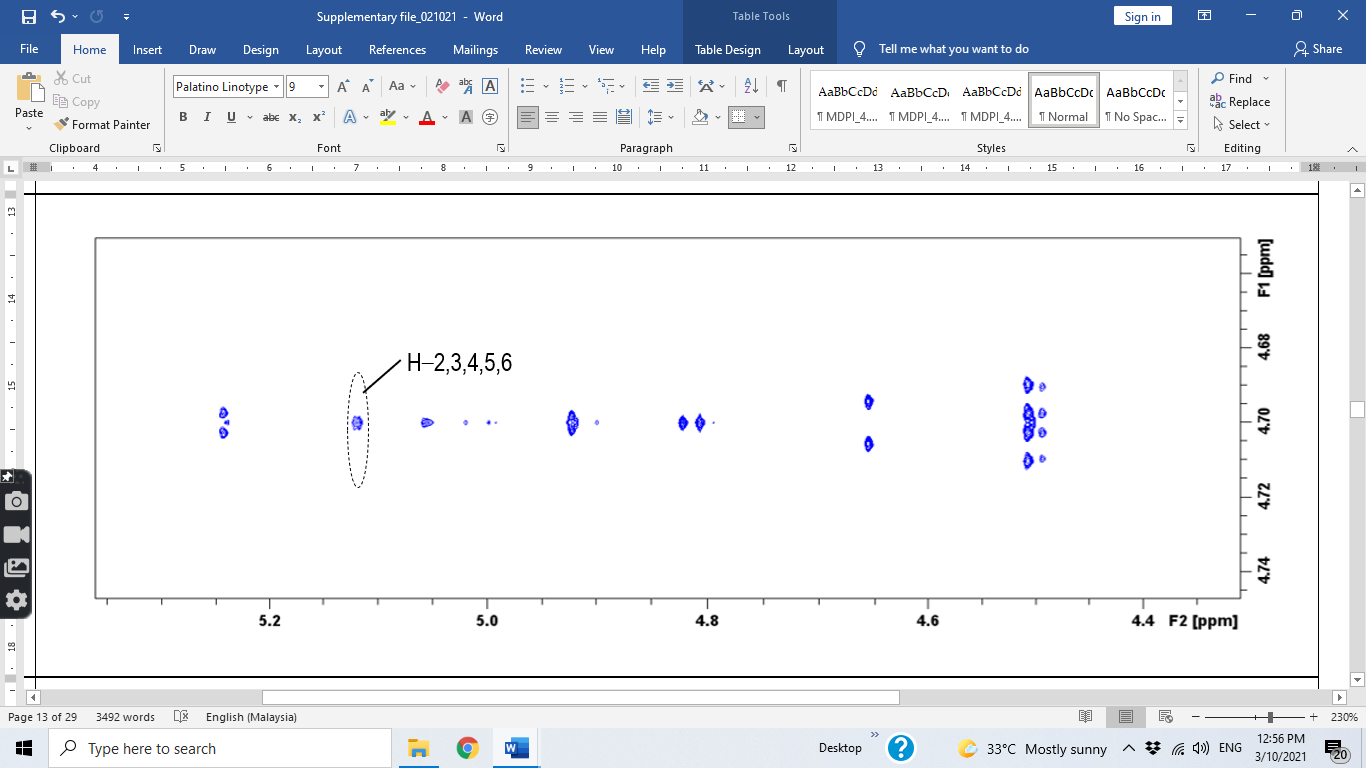

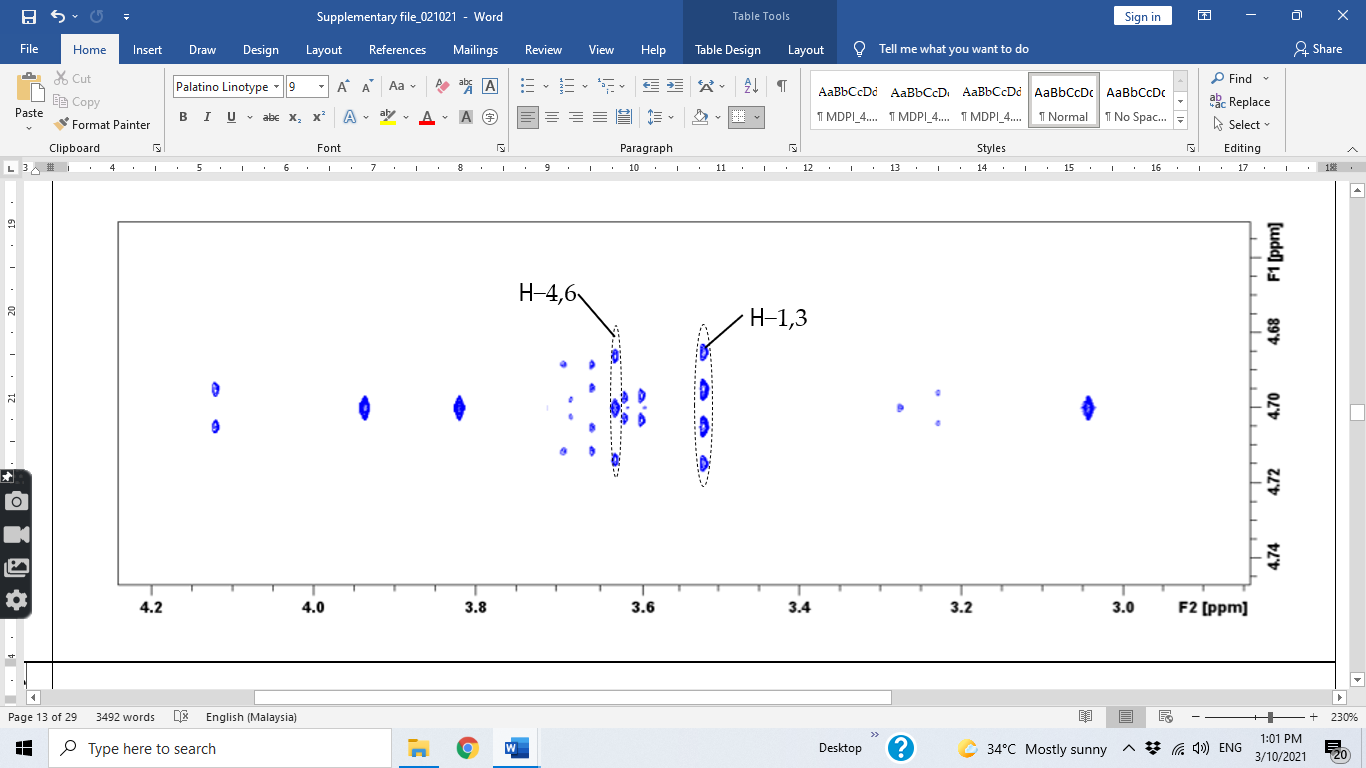


Mannose Myo-inositol


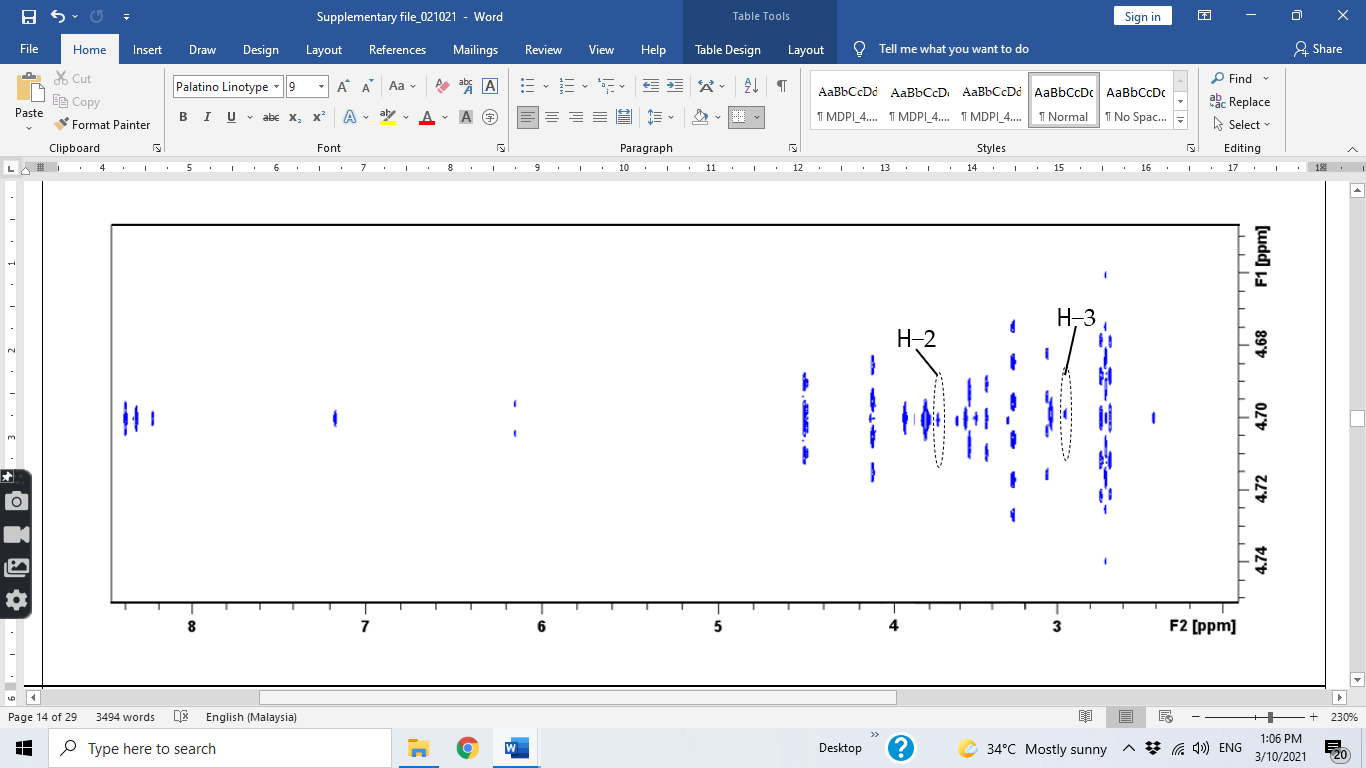

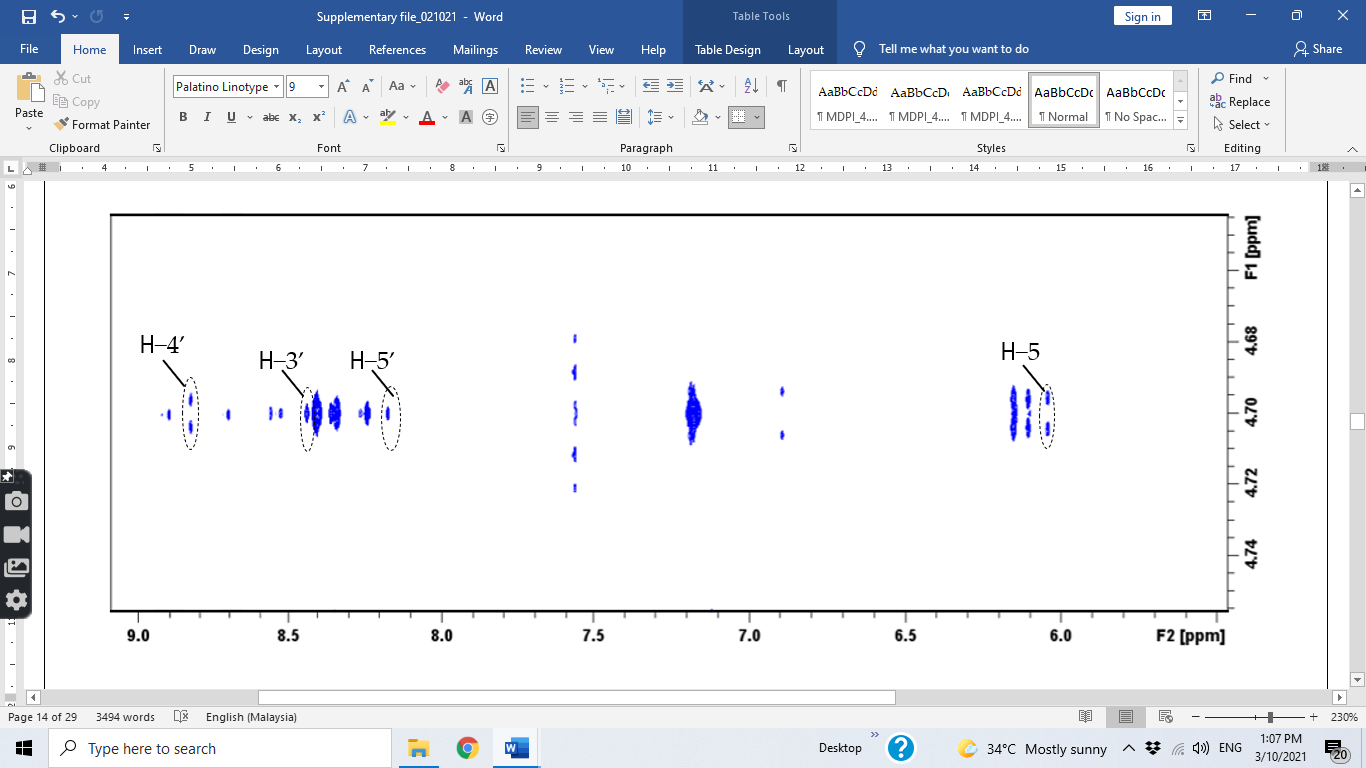


N,N-dimethylglycine NAD+


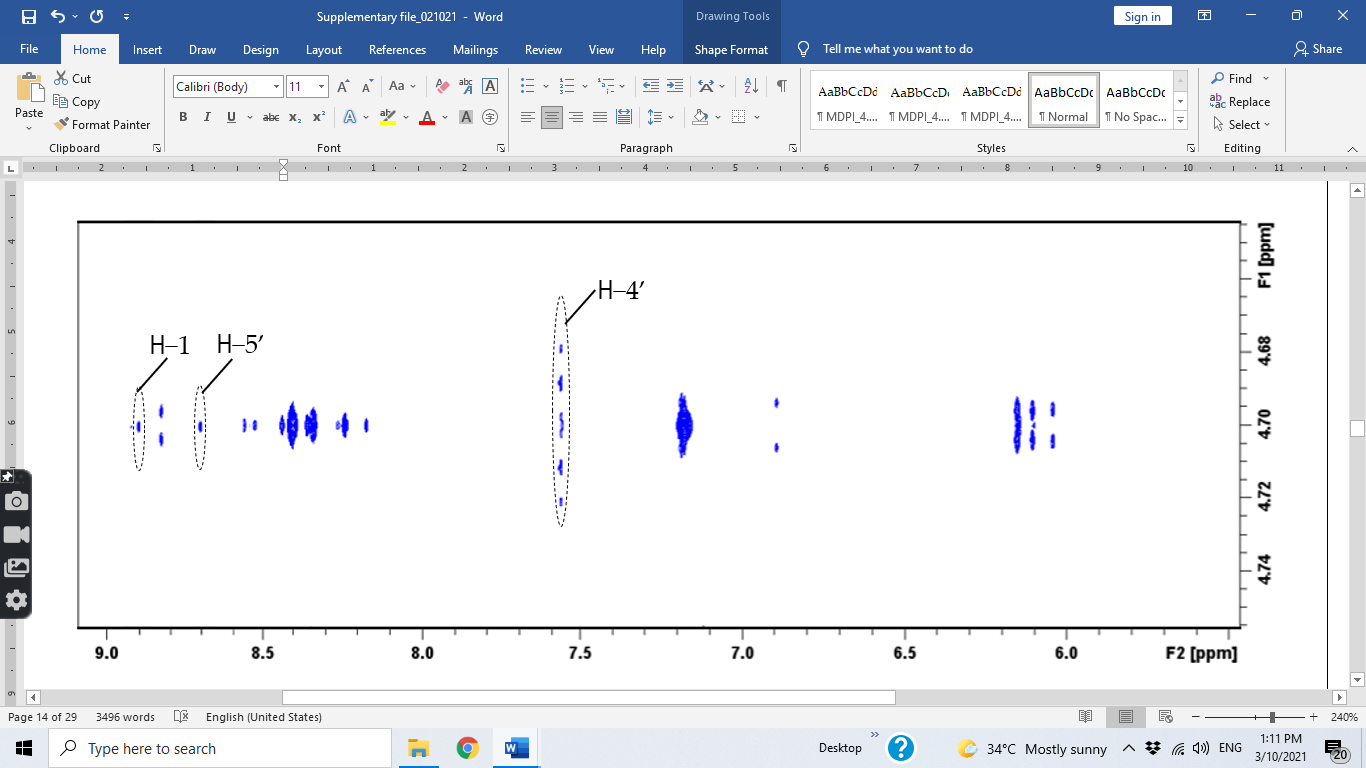

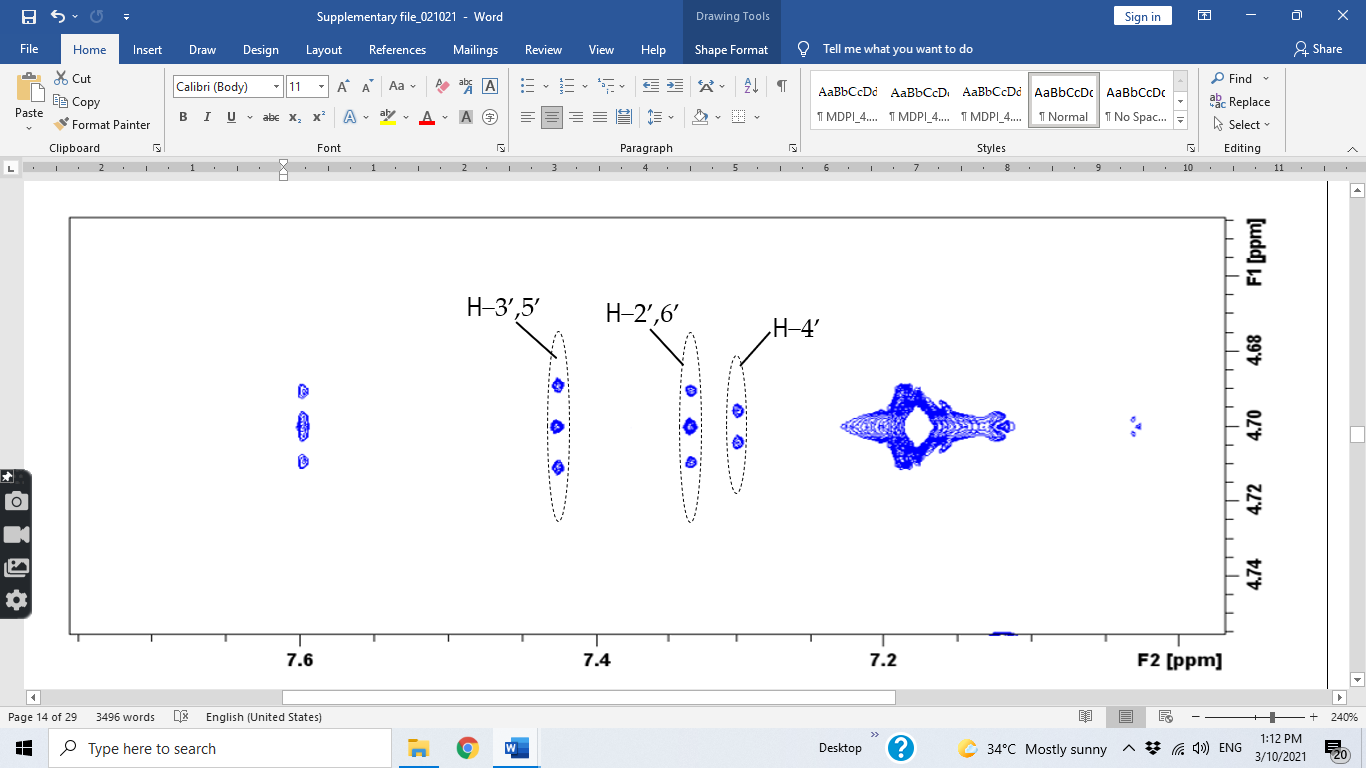


Niacinamide Phenylalanine


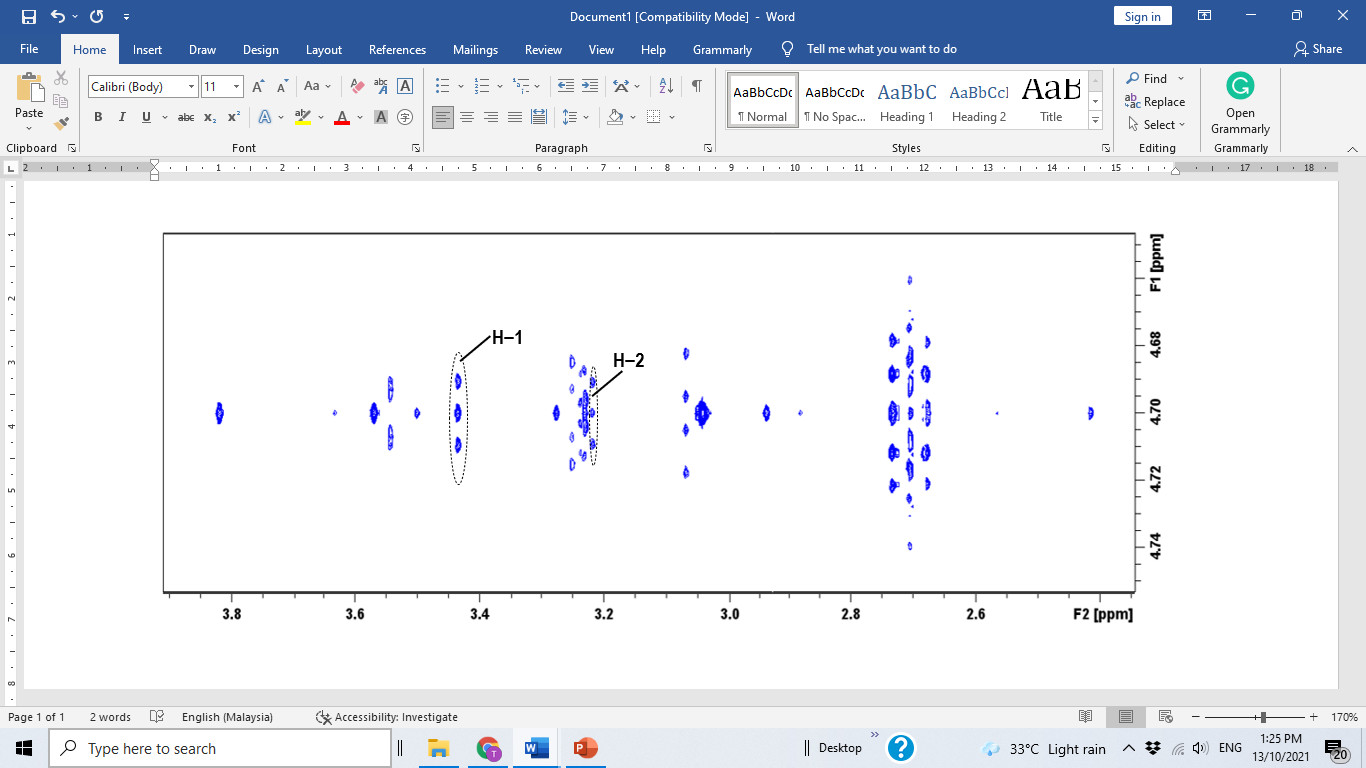

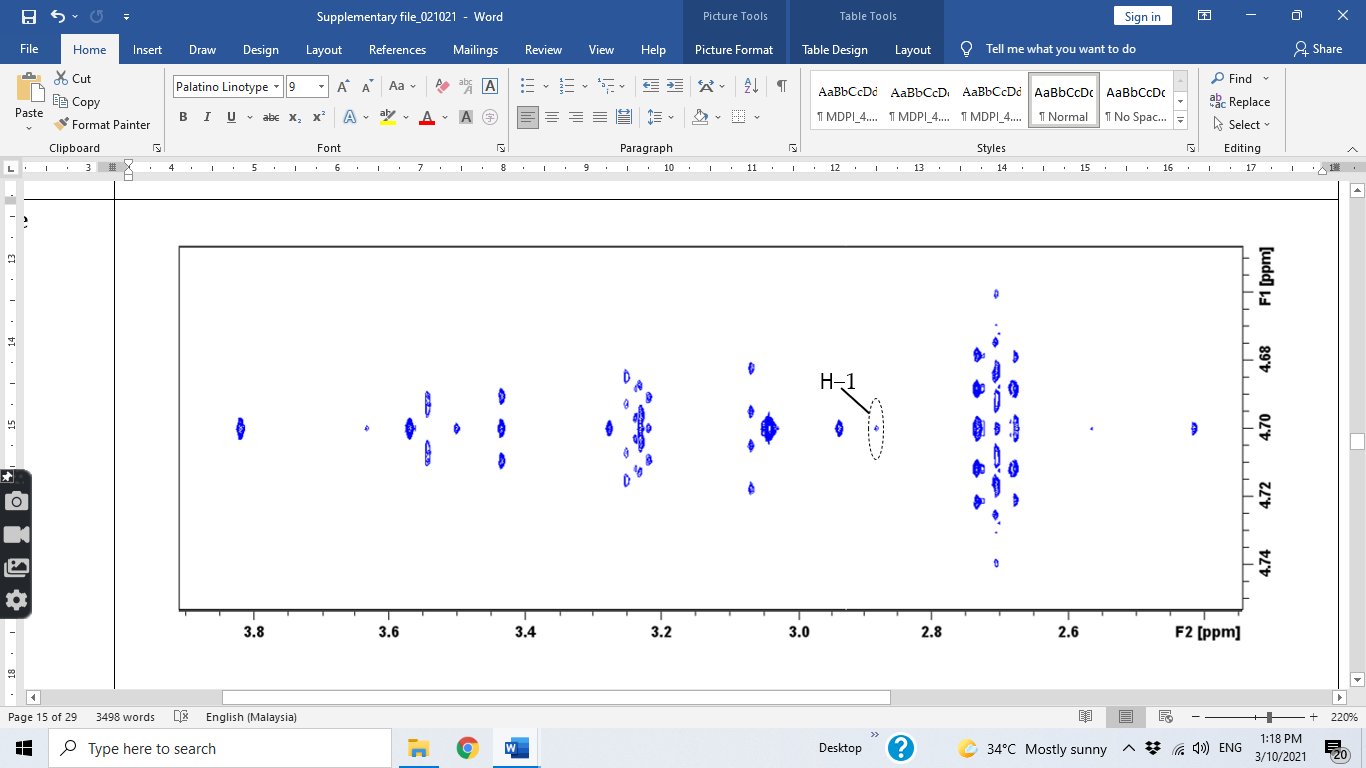


Taurine Trimethylamine


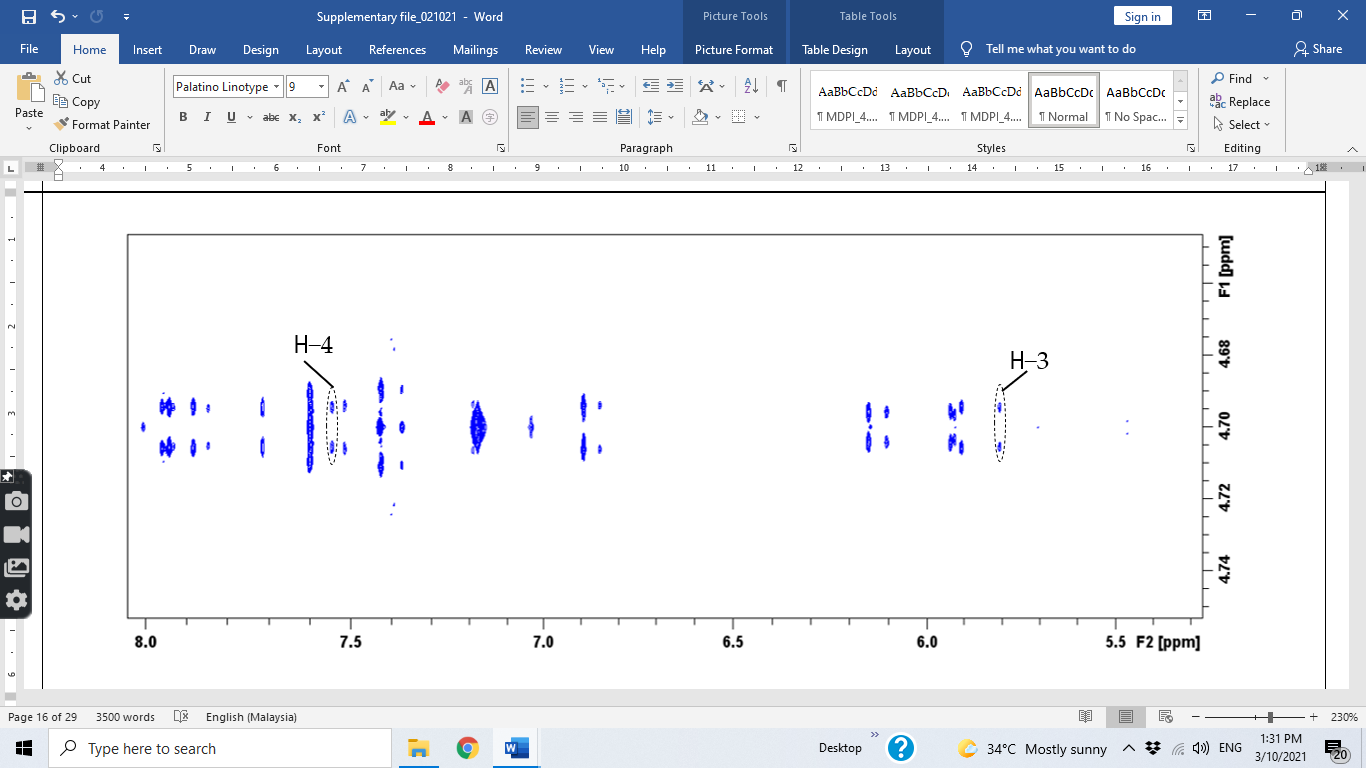

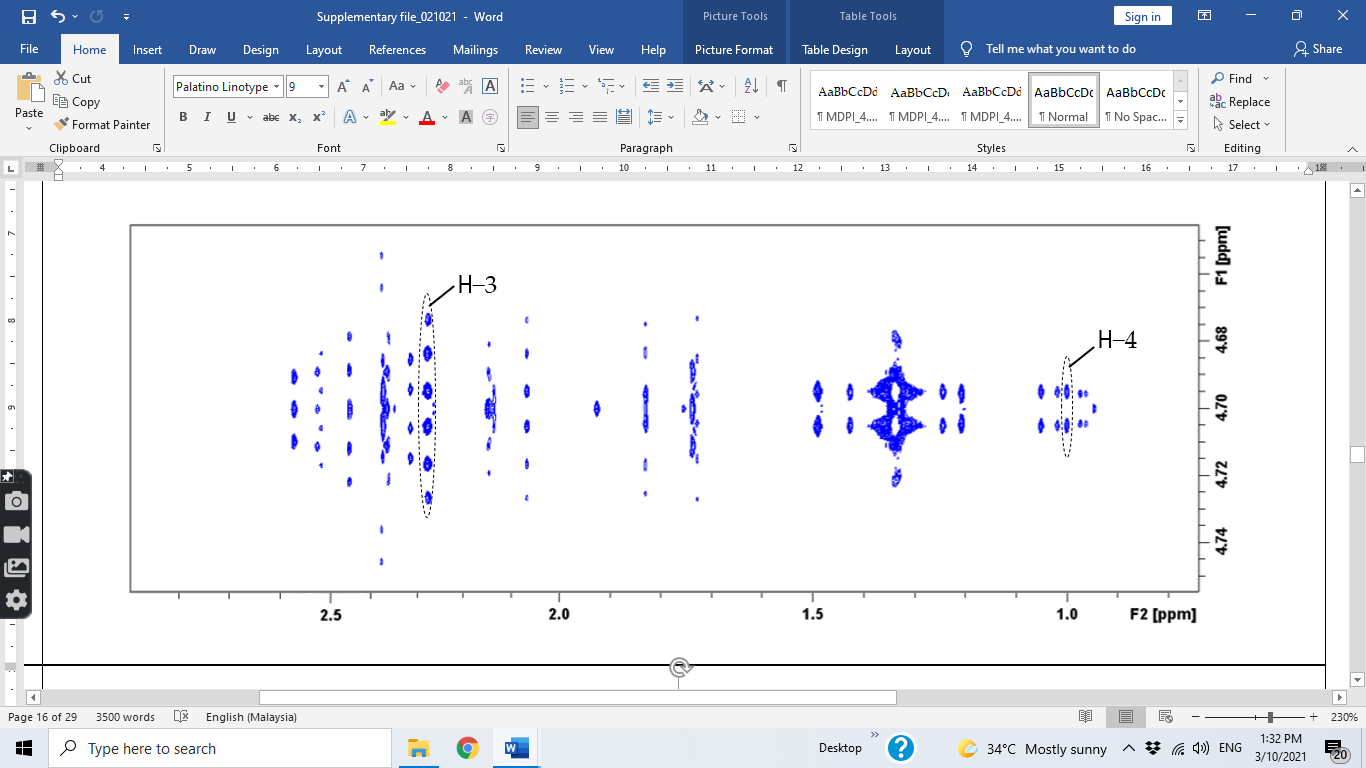


Uracil Valine


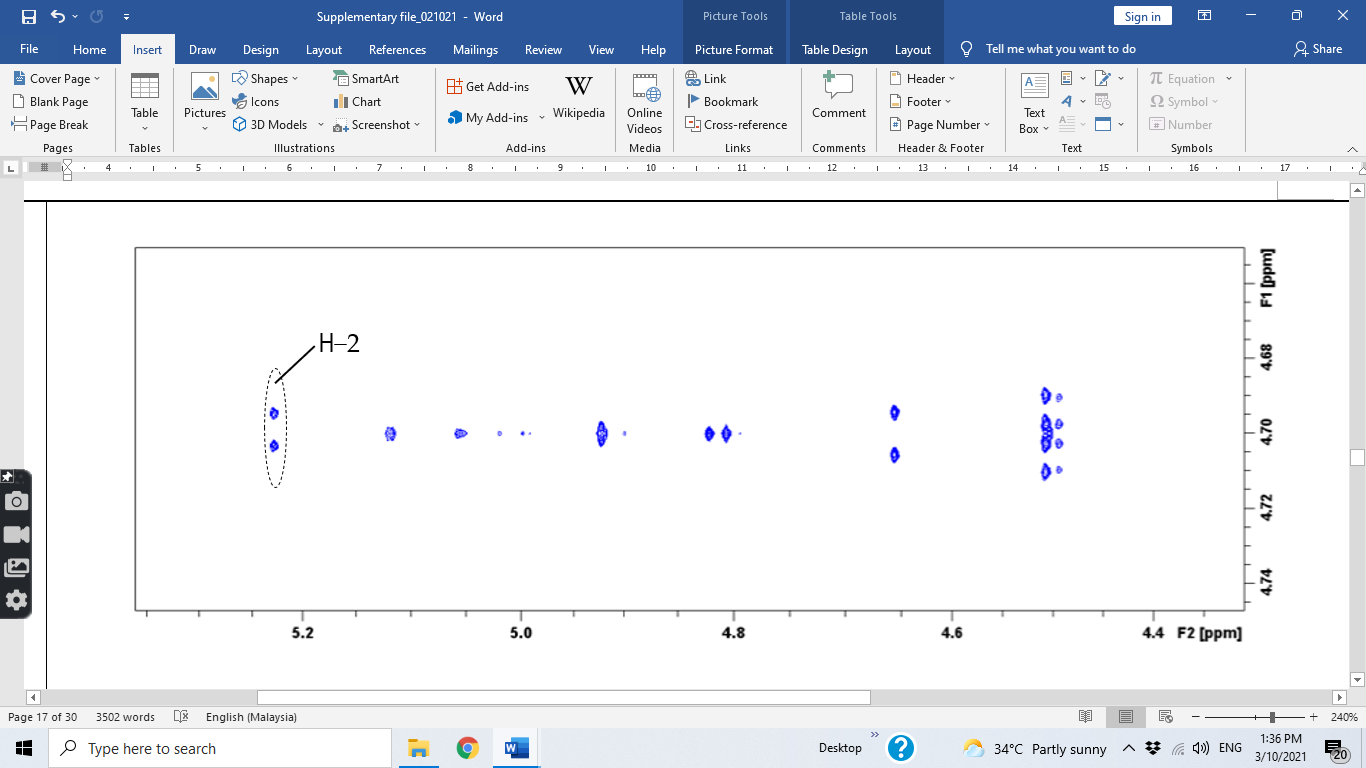

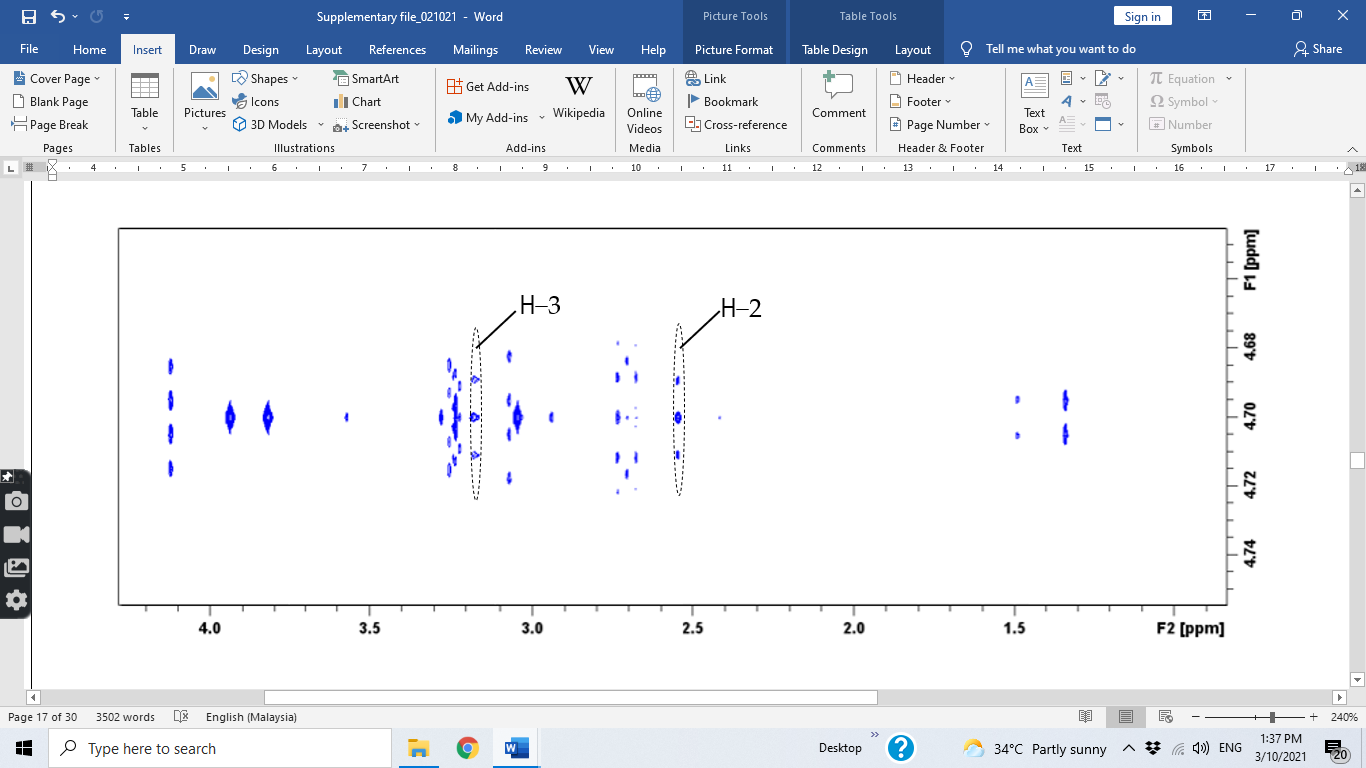


α−glucose β−alanine

**FIGURE** **S5 (**Continued)


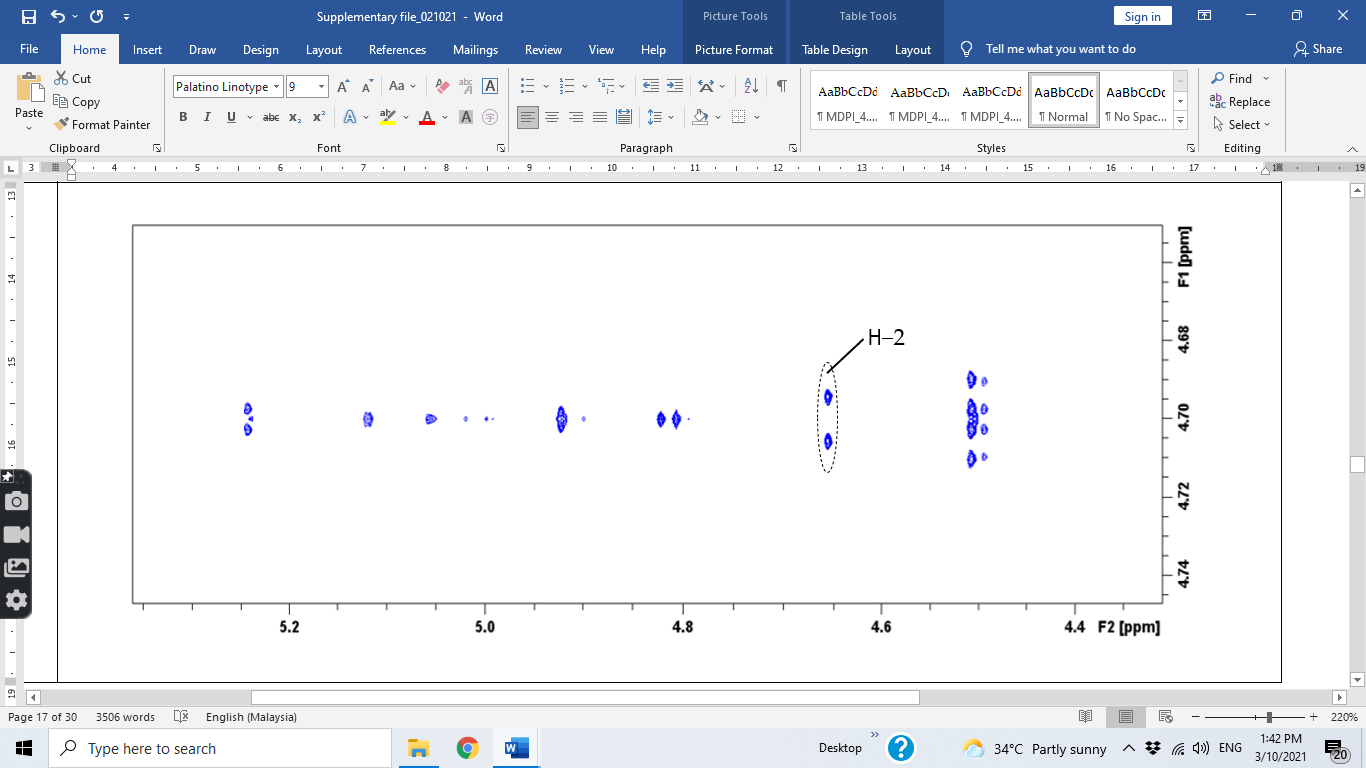


β−glucose

**FIGURE S5** 700 MHz 2D J-resolved NMR spectra for metabolites identified in pectoralis major


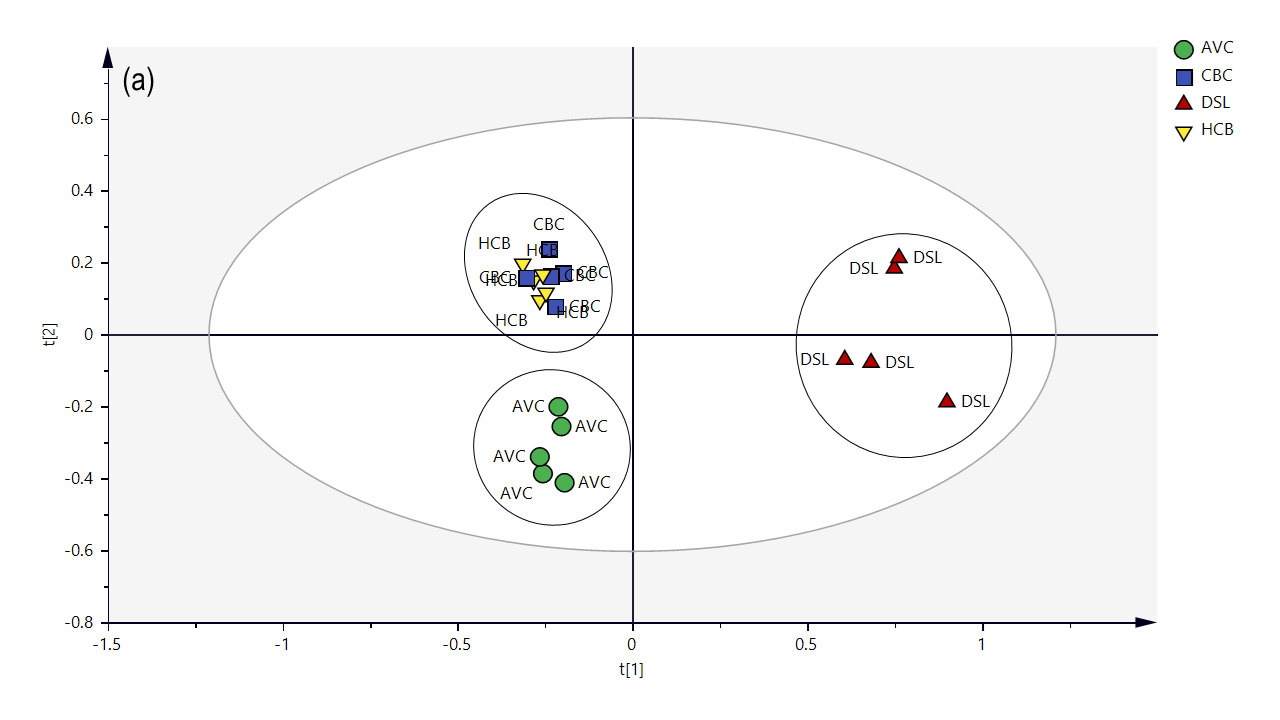


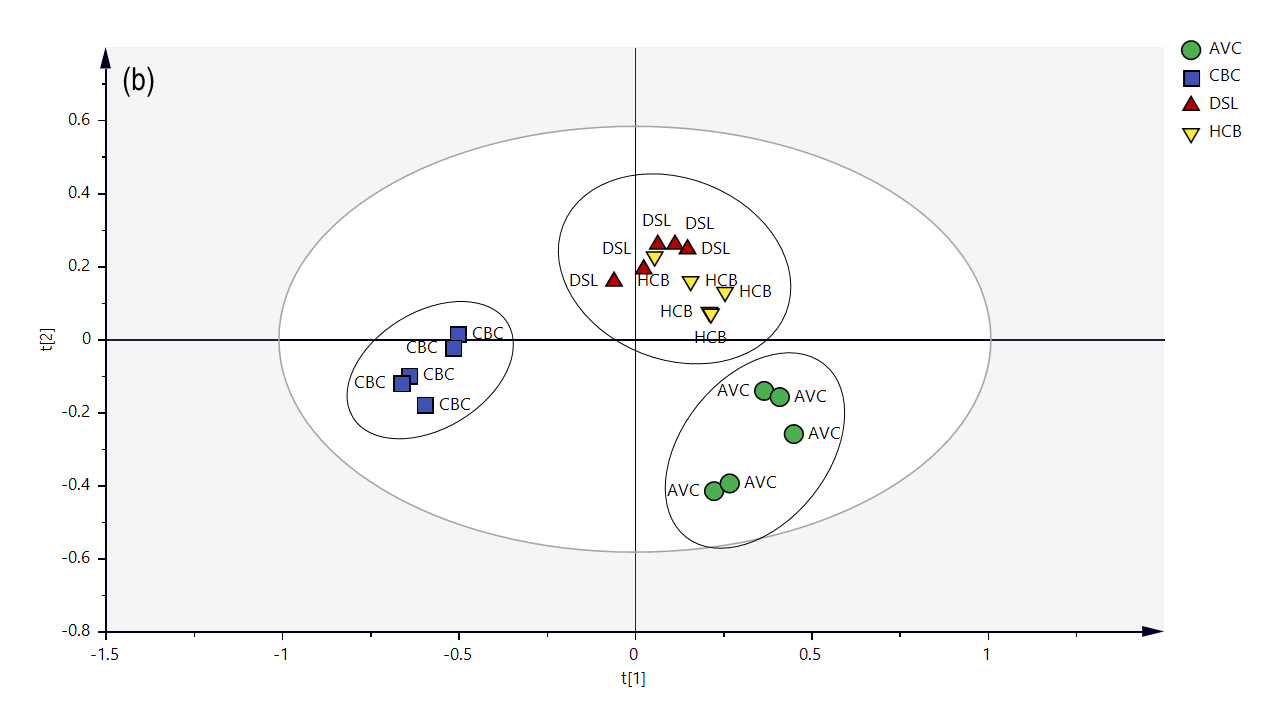


**FIGURE S6** Principal component analysis (PCA) score plots for chicken serum (a) and pectoralis major (b)

(a)

(b)

(c)

(d)

**FIGURE S7** Permutation test validation plots of OPLS- DA model in chicken serum for AVC (a), CBC (b), HCB (c) and DSL (d)

(a)

(b)

(c)

(d)

**FIGURE S8** Permutation test validation plots of OPLS-DA model in pectoralis major for AVC (a), CBC (b), HCB (c) and DSL (d)

**TABLE S1** R^2^Y and Q^2^Y intercept values of OPLS-DA model permutation test

| **Chicken Breeds** | **Matrices** | **R^2^Y** | **Q^2^Y** |
| --- | --- | --- | --- |
| AVC | Serum | 0.210 | -0.475 |
| CBC |  | 0.206 | -0.474 |
| HCB |  | 0.237 | -0.390 |
| DSL |  | 0.195 | -0.550 |
| AVC | Pectoralis major | 0.219 | -0.533 |
| CBC |  | 0.208 | -0.574 |
| HCB |  | 0.295 | -0.411 |
| DSL |  | 0.273 | -0.462 |

**TABLE S2** VIP values of characteristic metabolites in serum contributed to the group clustering in the OPLS-DA score scatter plot

| **No** | **Metabolites** | **Proton Number** | **Chemical Shift (ppm)** | **VIP**  **value** |
| --- | --- | --- | --- | --- |
| 1 | 3-hydroxybutyrate | (H-2) | 2.30 | 1.48620 |
|  | 3-hydroxybutyrate | (H-4) | 1.18 | 2.18911 |
| 2 | Acetone | (H-1,3) | 2.22 | 1.68976 |
| 3 | Alanine | (H-2) | 3.78 | 1.19604 |
| 4 | Betaine | (H-1) | 3.90 | 1.96747 |
| 5 | citrate | (H-1) | 2.54 | 1.11119 |
| 6 | Creatine | (H-2') | 3.02 | 1.22531 |
| 7 | Glutamine | (H-3) | 2.14 | 1.01583 |
|  | Glutamine | (H-4) | 2.46 | 1.07581 |
| 8 | glycine | (H-2) | 3.58 | 1.00730 |
| 9 | homoserine | (H-2) | 3.74 | 1.69546 |
| 10 | lactate | (H-2) | 4.14 | 1.25244 |
| 11 | Leucine | (H-5) | 0.94 | 1.04808 |
|  | Leucine | (H-3) | 1.70 | 1.08616 |
| 12 | lysine | (H-3) | 1.86 | 1.11267 |
| 13 | malate | (H-3) | 2.66 | 1.06441 |
|  | malate | (H-3) | 2.42 | 1.39720 |
| 14 | methionine | (H-4) | 2.62 | 1.06379 |
| 15 | Myo-inositol | (H-5) | 3.54 | 1.47711 |
| 16 | phenylalanine | (H-3) | 3.22 | 3.02553 |
| 17 | Succinate | (H-2,3) | 2.38 | 1.27586 |
| 18 | valine | (H-3) | 2.26 | 1.25996 |
| 19 | α-glucose | (H-5) | 5.22 | 1.35887 |

**TABLE S3** VIP values of characteristic metabolites in pectoralis major contributed to the group clustering in the OPLS-DA score scatter plot

| **No** | **Metabolites** | **Proton Number** | **Chemical Shift (ppm)** | **VIP**  **value** |
| --- | --- | --- | --- | --- |
| 1 | Alanine | (H-3) | 1.46 | 1.55781 |
| 2 | Anserine | (H-1) | 8.22 | 1.51606 |
| 3 | Betaine | (H-1) | 3.90 | 2.55317 |
| 4 | creatine | (H-2) | 3.94 | 2.07676 |
| 5 | Glycine | (H-2) | 3.58 | 1.50404 |
| 6 | Guanidoacetate | (H-2) | 3.82 | 4.03446 |
| 7 | hypoxanthine | (H-4) | 8.18 | 1.42303 |
| 8 | IMP | (H-3) | 4.46 | 1.82953 |
| 9 | Lactate | (H-2) | 4.14 | 2.10470 |
| 10 | Myo-inositol | (H-1,3) | 3.54 | 1.05612 |
| 11 | N,N-dimethylglycine | (H-3) | 2.94 | 2.52775 |
| 12 | NAD+ | (H-3') | 8.38 | 2.79011 |
| 13 | Phenylalanine | (H-2’, 6’) | 7.34 | 2.25474 |
| 14 | taurine | (H-2) | 3.22 | 3.36437 |
| 15 | β-alanine | (H-3) | 3.18 | 2.29450 |

**TABLE S4** Semi-quantitation on annotated metabolites for serum of different chicken breeds (mM)

| **Metabolite** | **Metabolite Classification** | **Authentic**  **village chicken** | | | | | **Coloured Broiler**  **(Hubbard)** | | | | | **Broiler**  **(Cobb)** | | | | | **Spent Layers**  **(Dekalb)** | | | | |
| --- | --- | --- | --- | --- | --- | --- | --- | --- | --- | --- | --- | --- | --- | --- | --- | --- | --- | --- | --- | --- | --- |
|  |  | **1** | **2** | **3** | **4** | **5** | **1** | **2** | **3** | **4** | **5** | **1** | **2** | **3** | **4** | **5** | **1** | **2** | **3** | **4** | **5** |
| 3−hydroxybutyrate | Organic acid | 3.4350 | 3.3234 | 4.7158 | 3.9099 | 2.5576 | 1.3124 | 1.0643 | 1.4080 | 1.2681 | 1.2239 | 2.2130 | 2.9839 | 2.3529 | 2.1191 | 2.3231 | 0.6252 | 0.4587 | 0.5588 | 0.6902 | 0.7851 |
| Acetone | Other | 0.0902 | 0.1549 | 0.1791 | 0.1750 | 0.1798 | 0.1281 | 0.1111 | 0.1576 | 0.1400 | 0.1078 | 0.1127 | 0.1026 | 0.0974 | 0.1523 | 0.1162 | 0.6036 | 0.6813 | 0.6941 | 0.9623 | 0.7813 |
| Alanine | Amino acid | 0.6464 | 0.7498 | 0.5031 | 0.8273 | 0.8168 | 1.3021 | 1.2341 | 1.4066 | 1.1186 | 0.8278 | 1.8656 | 1.8042 | 1.8878 | 1.7679 | 1.9803 | 0.9227 | 1.0690 | 0.8542 | 0.8307 | 0.9529 |
| Betaine | Amino acid | 0.5129 | 0.4118 | 0.4644 | 0.5706 | 0.4848 | 0.7537 | 0.9264 | 0.6040 | 1.1997 | 0.7052 | 1.1389 | 0.9993 | 1.0189 | 1.1757 | 0.9989 | 0.3653 | 0.3978 | 0.3672 | 0.2915 | 0.2748 |
| Citrate | Organic acid | 0.9052 | 0.9804 | 0.7926 | 0.8512 | 0.8324 | 0.6645 | 0.4306 | 0.4585 | 0.6217 | 0.6098 | 0.4904 | 0.6335 | 0.5767 | 0.7048 | 0.6306 | 0.3541 | 0.4047 | 0.1861 | 0.2964 | 0.2789 |
| Creatine | Amino acid | 0.9531 | 0.9234 | 1.0170 | 0.9590 | 0.8659 | 0.4415 | 0.6117 | 0.3546 | 0.8162 | 0.7832 | 0.2482 | 0.2237 | 0.2621 | 0.3065 | 0.2779 | 0.2675 | 0.4369 | 0.3557 | 0.4898 | 0.2958 |
| Creatinine | Amino acid | 0.1920 | 0.2375 | 0.2142 | 0.2667 | 0.2134 | 0.1972 | 0.2250 | 0.1833 | 0.2396 | 0.1565 | 0.1670 | 0.2310 | 0.2669 | 0.1944 | 0.2552 | 0.2493 | 0.2911 | 0.2342 | 0.3425 | 0.2340 |
| Formate | Other | 0.0908 | 0.1044 | 0.1142 | 0.0684 | 0.0976 | 0.1039 | 0.0928 | 0.0683 | 0.1148 | 0.0992 | 0.0925 | 0.0632 | 0.0920 | 0.1251 | 0.0865 | 0.1211 | 0.0935 | 0.1445 | 0.1088 | 0.1251 |
| Fumarate | Organic acid | 0.0344 | 0.0326 | 0.0252 | 0.0280 | 0.0260 | 0.0153 | 0.0156 | 0.0125 | 0.0183 | 0.0236 | 0.0242 | 0.0338 | 0.0296 | 0.0230 | 0.0285 | 0.0139 | 0.0230 | 0.0187 | 0.0215 | 0.0156 |
| Glutamine | Amino acid | 1.0530 | 1.1482 | 1.2670 | 0.9916 | 0.9243 | 0.7332 | 0.7345 | 0.4120 | 0.7133 | 0.7971 | 0.8059 | 0.8813 | 0.9050 | 0.8469 | 0.8123 | 0.6849 | 0.6617 | 0.8132 | 0.6636 | 0.6723 |
| Glycine | Amino acid | 2.2653 | 2.5476 | 2.1647 | 2.3969 | 2.3370 | 1.4846 | 1.3789 | 1.0411 | 1.6939 | 1.6193 | 1.4015 | 1.2979 | 1.5463 | 1.8144 | 1.5721 | 1.5594 | 1.9000 | 1.2627 | 1.8532 | 1.2826 |
| Homoserine | Amino acid | 1.7053 | 1.8488 | 1.5945 | 1.5499 | 2.0184 | 1.7406 | 1.8384 | 1.5014 | 1.1151 | 1.1662 | 2.7698 | 2.9309 | 3.1047 | 2.9134 | 2.1669 | 0.9660 | 0.8984 | 1.2853 | 0.8863 | 0.8724 |
| Hypoxanthine | Other | 0.0173 | 0.0264 | 0.0186 | 0.0295 | 0.0229 | 0.0206 | 0.0170 | 0.0182 | 0.0278 | 0.0218 | 0.0235 | 0.0377 | 0.0224 | 0.0294 | 0.0249 | 0.0133 | 0.0138 | 0.0154 | 0.0186 | 0.0189 |
| Lactate | Organic acid | 16.2868 | 17.2334 | 17.8219 | 16.1818 | 19.9519 | 7.3732 | 13.1044 | 9.4262 | 10.1493 | 8.2943 | 13.0787 | 12.9056 | 8.2931 | 10.2705 | 8.4295 | 11.0907 | 10.3985 | 8.2938 | 12.3008 | 13.5761 |
| Leucine | Amino acid | 1.3138 | 1.2571 | 1.3309 | 1.2741 | 1.3363 | 0.2574 | 0.2840 | 0.2435 | 0.3791 | 0.4333 | 0.2347 | 0.1883 | 0.2560 | 0.3007 | 0.2485 | 0.3363 | 0.3311 | 0.2500 | 0.3177 | 0.2807 |
| Lysine | Amino acid | 1.3199 | 1.4357 | 1.5979 | 1.6123 | 1.4946 | 0.2851 | 0.5543 | 0.4097 | 0.6349 | 0.4477 | 0.4280 | 0.3498 | 0.4597 | 0.3608 | 0.4238 | 0.4169 | 0.2469 | 0.3591 | 0.4345 | 0.3862 |
| Malate | Organic acid | 1.8008 | 1.3387 | 1.2594 | 1.0375 | 1.2363 | 0.4093 | 0.4207 | 0.4968 | 0.6288 | 0.7310 | 0.5338 | 0.8710 | 0.7657 | 0.8370 | 0.8030 | 0.5507 | 0.5462 | 0.4112 | 0.6174 | 0.6111 |
| Mannose | Sugar | 0.1752 | 0.0893 | 0.1255 | 0.1257 | 0.1034 | 0.1521 | 0.0930 | 0.1063 | 0.1084 | 0.0918 | 0.1243 | 0.1201 | 0.1762 | 0.1417 | 0.1646 | 0.1006 | 0.1211 | 0.1497 | 0.1274 | 0.1655 |
| Methionine | Amino acid | 0.2408 | 0.2920 | 0.2849 | 0.2831 | 0.3029 | 0.0950 | 0.1517 | 0.1094 | 0.1897 | 0.1838 | 0.0547 | 0.1340 | 0.1869 | 0.1613 | 0.1901 | 0.1014 | 0.1036 | 0.1082 | 0.1795 | 0.1204 |
| 3−methylhistidine | Amino acid | 0.0371 | 0.0381 | 0.0596 | 0.0308 | 0.0370 | 0.0332 | 0.0255 | 0.0273 | 0.0349 | 0.0300 | 0.0485 | 0.0412 | 0.0377 | 0.0649 | 0.0570 | 0.0526 | 0.0483 | 0.0336 | 0.0355 | 0.0350 |
| Myo−inositol | Sugar alcohol | 0.6134 | 0.6968 | 0.5609 | 0.7639 | 0.6369 | 0.5340 | 0.4394 | 0.3966 | 0.7608 | 0.4788 | 1.5073 | 1.6949 | 1.7363 | 2.5717 | 1.7397 | 0.3792 | 0.6492 | 0.5069 | 0.5578 | 0.5278 |
| O−phosphocholine | Other | 0.2319 | 0.3533 | 0.2352 | 0.3163 | 0.3561 | 0.1350 | 0.1219 | 0.1729 | 0.1509 | 0.2211 | 0.1576 | 0.2326 | 0.2604 | 0.2370 | 0.1803 | 0.1860 | 0.1640 | 0.2222 | 0.3093 | 0.1852 |
| Phenylalanine | Amino acid | 0.0788 | 0.0631 | 0.0648 | 0.0816 | 0.0642 | 0.3599 | 0.5543 | 0.3555 | 0.3841 | 0.4817 | 0.0620 | 0.0575 | 0.0775 | 0.0687 | 0.0744 | 0.0679 | 0.0723 | 0.0685 | 0.0642 | 0.0685 |
| Succinate | Organic acid | 0.4313 | 0.4242 | 0.6439 | 0.3337 | 0.3711 | 0.2850 | 0.1675 | 0.1928 | 0.2351 | 0.2207 | 0.2298 | 0.2377 | 0.3375 | 0.3465 | 0.2455 | 0.2201 | 0.1650 | 0.1297 | 0.1204 | 0.1463 |
| Trimethylamine N−oxide | Other | 0.5959 | 0.6258 | 0.6589 | 0.6382 | 0.6530 | 0.7570 | 0.5633 | 0.6034 | 0.6156 | 0.7191 | 0.6011 | 0.5678 | 0.6818 | 0.8011 | 0.6723 | 0.6816 | 0.6560 | 0.5607 | 0.5862 | 0.5682 |
| Tyrosine | Amino acid | 0.0824 | 0.0565 | 0.0592 | 0.1125 | 0.0839 | 0.0895 | 0.0608 | 0.0695 | 0.1077 | 0.0770 | 0.1044 | 0.1022 | 0.0962 | 0.0973 | 0.0838 | 0.0832 | 0.0980 | 0.0772 | 0.0642 | 0.0675 |
| Valine | Amino acid | 0.5002 | 0.3715 | 0.5035 | 0.4194 | 0.5100 | 0.3752 | 0.4954 | 0.4489 | 0.6228 | 0.8355 | 0.3707 | 0.2828 | 0.4009 | 0.4455 | 0.3826 | 0.8847 | 0.7985 | 0.9483 | 0.8631 | 0.8485 |
| Xanthine | Other | 0.0939 | 0.1007 | 0.0743 | 0.0978 | 0.0847 | 0.1165 | 0.1011 | 0.0772 | 0.1363 | 0.1086 | 0.0881 | 0.0754 | 0.1110 | 0.1340 | 0.1068 | 0.1237 | 0.1108 | 0.1011 | 0.1231 | 0.1339 |
| α−glucose | Sugar | 0.1794 | 0.1525 | 0.1967 | 0.1268 | 0.1624 | 0.3914 | 0.3894 | 0.4460 | 0.3253 | 0.2881 | 0.8374 | 0.9726 | 0.8305 | 0.7009 | 0.9264 | 0.1316 | 0.0969 | 0.1233 | 0.1296 | 0.0767 |
| β−glucose | Sugar | 0.1794 | 0.1525 | 0.1967 | 0.1268 | 0.1624 | 0.3914 | 0.3894 | 0.4460 | 0.3253 | 0.2881 | 0.8374 | 0.9726 | 0.8305 | 0.7009 | 0.9264 | 0.1316 | 0.0969 | 0.1233 | 0.1296 | 0.0767 |

**TABLE S5** Semi-quantitation on annotated metabolites for pectoralis major of different chicken breeds (mM)

| **Metabolite** | **Metabolite Classification** | **Authentic**  **village chicken** | | | | | **Coloured Broiler**  **(Hubbard)** | | | | | **Broiler**  **(Cobb)** | | | | | **Spent Layers**  **(Dekalb)** | | | | |
| --- | --- | --- | --- | --- | --- | --- | --- | --- | --- | --- | --- | --- | --- | --- | --- | --- | --- | --- | --- | --- | --- |
|  |  | **1** | **2** | **3** | **4** | **5** | **1** | **2** | **3** | **4** | **5** | **1** | **2** | **3** | **4** | **5** | **1** | **2** | **3** | **4** | **5** |
| 3−hydroxybutyrate | Organic acid | 0.2032 | 0.2129 | 0.1805 | 0.1934 | 0.2156 | 0.2177 | 0.2284 | 0.2570 | 0.2808 | 0.1552 | 0.3579 | 0.3222 | 0.3367 | 0.2300 | 0.3102 | 0.2109 | 0.2280 | 0.2154 | 0.1824 | 0.1745 |
| Acetate | Other | 0.1607 | 0.1153 | 0.1075 | 0.1105 | 0.1248 | 0.1774 | 0.1631 | 0.1258 | 0.1732 | 0.1675 | 0.1720 | 0.1553 | 0.1718 | 0.1445 | 0.1781 | 0.1974 | 0.1706 | 0.1167 | 0.2478 | 0.1514 |
| Alanine | Amino acid | 0.6587 | 0.8612 | 1.0855 | 1.2174 | 0.9058 | 0.9670 | 0.7179 | 1.0967 | 0.9813 | 1.1475 | 2.1060 | 1.3412 | 2.2009 | 2.0128 | 1.9533 | 0.6203 | 1.0366 | 0.8082 | 1.0993 | 1.2384 |
| Anserine | Peptide | 28.6769 | 27.4875 | 27.3935 | 28.7954 | 27.1835 | 5.2667 | 5.6841 | 5.0503 | 5.8363 | 5.4754 | 5.7086 | 4.0858 | 4.6933 | 4.9301 | 4.2320 | 4.1744 | 4.0170 | 4.4635 | 5.1327 | 5.2650 |
| Betaine | Amino acid | 0.8429 | 0.8519 | 0.9432 | 0.8502 | 0.8697 | 0.9166 | 0.9014 | 0.8932 | 0.9479 | 0.9296 | 3.8011 | 3.8144 | 3.7538 | 2.7942 | 3.6492 | 0.4833 | 0.6047 | 0.6696 | 0.5555 | 0.5759 |
| Creatine | Amino acid | 14.5343 | 18.2913 | 14.7331 | 16.2967 | 20.6496 | 26.8696 | 27.6356 | 26.5173 | 27.6311 | 27.4276 | 18.9267 | 17.9302 | 21.3259 | 20.8140 | 18.8251 | 32.1466 | 29.6759 | 31.1619 | 31.2459 | 30.1097 |
| Formate | Other | 0.1743 | 0.1479 | 0.1646 | 0.1673 | 0.1605 | 0.1623 | 0.1552 | 0.1403 | 0.2285 | 0.1522 | 0.1817 | 0.1896 | 0.1649 | 0.1792 | 0.1784 | 0.2129 | 0.1545 | 0.1637 | 0.2012 | 0.1378 |
| Fumarate | Organic acid | 0.0046 | 0.0034 | 0.0043 | 0.0051 | 0.0036 | 0.0041 | 0.0039 | 0.0026 | 0.0033 | 0.0031 | 0.0029 | 0.0044 | 0.0058 | 0.0039 | 0.0032 | 0.0029 | 0.0038 | 0.0037 | 0.0034 | 0.0041 |
| Glutamate | Amino acid | 0.8905 | 0.7104 | 0.7317 | 0.8355 | 0.7658 | 0.8260 | 0.5000 | 0.4364 | 0.5296 | 0.6400 | 0.5357 | 0.5367 | 0.5955 | 0.6204 | 0.5707 | 0.4151 | 0.4682 | 0.5854 | 0.5547 | 0.5709 |
| Glutamine | Amino acid | 0.5835 | 0.6312 | 0.5477 | 0.6776 | 0.6086 | 0.5811 | 0.4725 | 0.5726 | 0.7065 | 1.0850 | 0.6044 | 0.6286 | 0.6387 | 0.5759 | 0.5272 | 0.4654 | 0.8408 | 0.4614 | 0.6678 | 0.6759 |
| Glycine | Amino acid | 1.6846 | 1.7077 | 1.6002 | 1.4222 | 1.4307 | 0.8890 | 0.8936 | 1.0141 | 1.0031 | 1.2490 | 2.5953 | 2.9357 | 4.3121 | 3.1803 | 2.6345 | 0.6004 | 0.9586 | 0.5572 | 0.8235 | 0.7692 |
| Guanidoacetate | Amino acid | 47.7935 | 46.7046 | 41.4775 | 44.2673 | 36.8471 | 47.9079 | 47.8748 | 40.5452 | 55.4266 | 42.2970 | 64.3547 | 64.0515 | 62.5721 | 67.4080 | 63.0922 | 48.2840 | 47.2706 | 42.9475 | 51.5826 | 43.5582 |
| Hypoxanthine | Other | 0.8198 | 0.7831 | 0.8217 | 0.8490 | 0.7787 | 0.2085 | 0.1677 | 0.2146 | 0.2810 | 0.2168 | 0.3198 | 0.2394 | 0.2994 | 0.2772 | 0.3956 | 0.2273 | 0.1412 | 0.1519 | 0.1907 | 0.2153 |
| IMP | Nucleotide | 3.4000 | 3.6066 | 3.2088 | 3.5760 | 3.2306 | 1.2060 | 0.7691 | 0.7858 | 1.1848 | 0.9110 | 1.2493 | 0.9216 | 1.0129 | 0.9941 | 1.1270 | 0.7313 | 0.5008 | 0.9059 | 0.5765 | 0.6178 |
| Lactate | Organic acid | 75.0772 | 70.9105 | 77.6369 | 79.8482 | 74.9830 | 22.2895 | 25.0224 | 21.2247 | 31.5405 | 21.4415 | 18.5575 | 14.8472 | 12.9516 | 16.8734 | 20.8780 | 18.9742 | 25.0361 | 16.7709 | 17.1060 | 21.1994 |
| Leucine | Amino acid | 0.2935 | 0.2752 | 0.2682 | 0.3321 | 0.3372 | 0.2804 | 0.2429 | 0.2614 | 0.3393 | 0.3824 | 0.3117 | 0.3105 | 0.3075 | 0.4338 | 0.3425 | 0.2551 | 0.4210 | 0.2968 | 0.4605 | 0.3359 |
| Mannose | Sugar | 0.1751 | 0.1407 | 0.1469 | 0.0984 | 0.1303 | 0.1710 | 0.1023 | 0.1107 | 0.1382 | 0.1106 | 0.1437 | 0.1426 | 0.1320 | 0.1256 | 0.1723 | 0.1746 | 0.1456 | 0.2504 | 0.1815 | 0.1306 |
| Myo−inositol | Sugar alcohol | 0.4394 | 0.4684 | 0.3955 | 0.5910 | 0.3261 | 0.5228 | 0.4442 | 0.4896 | 0.5580 | 0.4439 | 0.7689 | 0.7561 | 0.9679 | 0.8145 | 0.8822 | 0.5758 | 0.3999 | 0.5517 | 0.4046 | 0.3708 |
| N,N−dimethylglycine | Amino acid | 0.0951 | 0.1650 | 0.1076 | 0.1216 | 0.0865 | 0.1353 | 0.1277 | 0.1416 | 0.1582 | 0.1786 | 0.4904 | 0.4176 | 0.6782 | 0.7248 | 0.5914 | 0.1364 | 0.0880 | 0.0928 | 0.1024 | 0.0909 |
| NAD+ | Nucleotide | 0.2981 | 0.3655 | 0.2971 | 0.3357 | 0.2838 | 0.3801 | 0.4389 | 0.2908 | 0.3763 | 0.4127 | 0.7155 | 0.7960 | 0.7257 | 0.7467 | 0.7595 | 0.4238 | 0.3709 | 0.4089 | 0.4459 | 0.3495 |
| Niacinamide | Vitamin | 0.1433 | 0.1301 | 0.1907 | 0.1903 | 0.1968 | 0.1473 | 0.1620 | 0.1532 | 0.1091 | 0.1445 | 0.1160 | 0.1871 | 0.1178 | 0.1060 | 0.1203 | 0.2090 | 0.1755 | 0.1049 | 0.1828 | 0.1087 |
| Phenylalanine | Amino acid | 0.0820 | 0.0728 | 0.0558 | 0.0671 | 0.0500 | 0.2954 | 0.3474 | 0.2874 | 0.3210 | 0.2984 | 0.1486 | 0.1175 | 0.1089 | 0.1221 | 0.1339 | 0.1906 | 0.1656 | 0.1979 | 0.1989 | 0.1837 |
| Taurine | Amino acid | 0.3629 | 0.3271 | 0.4096 | 0.6015 | 0.3369 | 1.4600 | 1.4870 | 1.2782 | 1.3520 | 1.4233 | 0.2480 | 0.2773 | 0.2016 | 0.2736 | 0.2209 | 1.7997 | 1.5772 | 1.3257 | 1.6680 | 1.6045 |
| Trimethylamine | Other | 0.0072 | 0.0058 | 0.0035 | 0.0044 | 0.0046 | 0.0074 | 0.0067 | 0.0085 | 0.0107 | 0.0127 | 0.0207 | 0.0230 | 0.0209 | 0.0271 | 0.0172 | 0.0089 | 0.0121 | 0.0065 | 0.0139 | 0.0110 |
| Uracil | Other | 0.0060 | 0.0089 | 0.0096 | 0.0091 | 0.0081 | 0.0162 | 0.0108 | 0.0130 | 0.0136 | 0.0179 | 0.0167 | 0.0150 | 0.0267 | 0.0245 | 0.0232 | 0.0063 | 0.0049 | 0.0069 | 0.0055 | 0.0055 |
| Valine | Amino acid | 0.2880 | 0.2476 | 0.3343 | 0.2822 | 0.2968 | 0.2916 | 0.2196 | 0.2289 | 0.3818 | 0.3066 | 0.2799 | 0.2515 | 0.2507 | 0.2472 | 0.2692 | 0.2645 | 0.2158 | 0.2143 | 0.2327 | 0.2949 |
| α−glucose | Sugar | 0.6479 | 0.9677 | 0.7632 | 0.7566 | 0.9191 | 0.6519 | 1.0345 | 1.0123 | 0.5887 | 0.8984 | 0.7482 | 0.6620 | 0.9687 | 0.7906 | 0.9358 | 0.8575 | 0.5965 | 0.9340 | 0.8083 | 0.9190 |
| β−alanine | Amino acid | 2.2159 | 2.2713 | 2.2093 | 2.5734 | 2.3011 | 0.3293 | 0.2112 | 0.3276 | 0.2221 | 0.2728 | 0.8658 | 0.9500 | 1.2463 | 1.0288 | 1.0841 | 1.6959 | 1.8104 | 1.7727 | 1.5426 | 1.7703 |
| β−glucose | Sugar | 0.6479 | 0.9677 | 0.7632 | 0.7566 | 0.9191 | 0.6519 | 1.0345 | 1.0123 | 0.5887 | 0.8984 | 0.7482 | 0.6620 | 0.9687 | 0.7906 | 0.9358 | 0.8575 | 0.5965 | 0.9340 | 0.8083 | 0.9190 |

**Table S6** Comparison of relative intensity mean values of ^1^H resonances for identified metabolites in serum from any two chicken breeds between clusters

| **Metabolite** | **AVC vs HCB** | | **AVC vs DSL** | | **AVC vs CBC** | | **CBC vs DSL** | | **HCB vs DSL** | |
| --- | --- | --- | --- | --- | --- | --- | --- | --- | --- | --- |
|  | **FI** | **P*** | **FI** | **P*** | **FI** | **P*** | **FI** | **P*** | **FI** | **P*** |
| 3−hydroxybutyrate | 2.858 | < 0.05 | 5.754 | < 0.05 | 1.496 | < 0.05 | 3.846 | < 0.05 | 2.013 | < 0.05 |
| Acetone | 1.209 | 0.203 | 0.209 | < 0.05 | 1.340 | 0.078 | 0.156 | < 0.05 | 0.173 | < 0.05 |
| Alanine | 0.602 | < 0.05 | 0.765 | < 0.05 | 0.381 | < 0.05 | 2.010 | < 0.05 | 1.272 | < 0.05 |
| Betaine | 0.584 | < 0.05 | 1.441 | < 0.05 | 0.458 | < 0.05 | 3.143 | < 0.05 | 2.469 | < 0.05 |
| Citrate | 1.566 | < 0.05 | 2.869 | < 0.05 | 1.437 | < 0.05 | 1.997 | < 0.05 | 1.832 | < 0.05 |
| Creatine | 1.569 | < 0.05 | 2.556 | < 0.05 | 3.579 | < 0.05 | 0.714 | < 0.05 | 1.629 | < 0.05 |
| Creatinine | 1.122 | 0.246 | 0.832 | 0.100 | 1.008 | 0.936 | 0.825 | 0.130 | 0.741 | < 0.05 |
| Formate | 0.992 | 0.949 | 0.802 | 0.075 | 1.035 | 0.804 | 0.775 | 0.074 | 0.808 | 0.083 |
| Fumarate | 1.714 | < 0.05 | 1.577 | < 0.05 | 1.051 | 0.609 | 1.501 | < 0.05 | 0.920 | 0.576 |
| Glutamine | 1.588 | < 0.05 | 1.540 | < 0.05 | 1.266 | < 0.05 | 1.216 | < 0.05 | 0.970 | 0.782 |
| Glycine | 1.623 | < 0.05 | 1.490 | < 0.05 | 1.534 | < 0.05 | 0.971 | 0.787 | 0.919 | 0.490 |
| Homoserine | 1.184 | 0.149 | 1.776 | < 0.05 | 0.628 | < 0.05 | 2.829 | < 0.05 | 1.500 | < 0.05 |
| Hypoxanthine | 1.088 | 0.549 | 1.434 | < 0.05 | 0.832 | 0.236 | 1.724 | < 0.05 | 1.318 | 0.051 |
| Lactate | 1.809 | < 0.05 | 1.572 | < 0.05 | 1.651 | < 0.05 | 0.952 | 0.706 | 0.869 | 0.302 |
| Leucine | 4.077 | < 0.05 | 4.296 | < 0.05 | 5.302 | < 0.05 | 0.810 | < 0.05 | 1.054 | 0.698 |
| Lysine | 3.200 | < 0.05 | 4.047 | < 0.05 | 3.689 | < 0.05 | 1.097 | 0.389 | 1.265 | 0.193 |
| Malate | 2.484 | < 0.05 | 2.438 | < 0.05 | 1.751 | < 0.05 | 1.392 | < 0.05 | 0.982 | 0.894 |
| Mannose | 1.122 | 0.481 | 0.932 | 0.637 | 0.852 | 0.272 | 1.094 | 0.450 | 0.830 | 0.190 |
| Methionine | 1.924 | < 0.05 | 2.290 | < 0.05 | 1.931 | < 0.05 | 1.186 | 0.452 | 1.190 | 0.361 |
| 3−methylhistidine | 1.343 | 0.084 | 0.988 | 0.941 | 0.813 | 0.221 | 1.216 | 0.202 | 0.736 | < 0.05 |
| Myo−inositol | 1.254 | 0.106 | 1.248 | < 0.05 | 0.354 | < 0.05 | 3.529 | < 0.05 | 0.996 | 0.977 |
| O−phosphocholine | 1.862 | < 0.05 | 1.399 | 0.053 | 1.398 | < 0.05 | 1.001 | 0.994 | 0.752 | 0.127 |
| Phenylalanine | 0.165 | < 0.05 | 1.033 | 0.611 | 1.036 | 0.662 | 0.996 | 0.949 | 6.255 | < 0.05 |
| Succinate | 2.002 | < 0.05 | 2.820 | < 0.05 | 1.578 | < 0.05 | 1.788 | < 0.05 | 1.409 | < 0.05 |
| Trimethylamine N−oxide | 0.973 | 0.664 | 1.039 | 0.402 | 0.954 | 0.487 | 1.089 | 0.282 | 1.067 | 0.379 |
| Tyrosine | 0.975 | 0.882 | 1.011 | 0.942 | 0.815 | 0.135 | 1.240 | < 0.05 | 1.037 | 0.784 |
| Valine | 0.830 | 0.300 | 0.531 | < 0.05 | 1.224 | 0.060 | 0.433 | < 0.05 | 0.640 | < 0.05 |
| Xanthine | 0.836 | 0.141 | 0.762 | < 0.05 | 0.876 | 0.285 | 0.870 | 0.218 | 0.911 | 0.373 |
| α−glucose | 0.444 | < 0.05 | 1.465 | < 0.05 | 0.192 | < 0.05 | 7.647 | < 0.05 | 3.297 | < 0.05 |
| β−glucose | 0.444 | < 0.05 | 1.465 | < 0.05 | 0.192 | < 0.05 | 7.647 | < 0.05 | 3.297 | < 0.05 |

* Statistically significance was indicated by P < 0.05

**Table S7** Comparison of relative intensity mean values of ^1^H resonances for identified metabolites in pectoralis major from any two chicken breeds between clusters

| **Metabolite** | **AVC vs HCB** | | **AVC vs DSL** | | **AVC vs CBC** | | **CBC vs DSL** | | **CBC vs HCB** | |
| --- | --- | --- | --- | --- | --- | --- | --- | --- | --- | --- |
|  | **FI** | **P*** | **FI** | **P*** | **FI** | **P*** | **FI** | **P*** | **FI** | **P*** |
| 3−hydroxybutyrate | 0.883 | 0.264 | 0.994 | 0.928 | 0.646 | < 0.05 | 1.540 | < 0.05 | 1.367 | < 0.05 |
| Acetate | 0.767 | < 0.05 | 0.700 | 0.059 | 0.753 | < 0.05 | 0.930 | 0.603 | 1.018 | 0.799 |
| Alanine | 0.963 | 0.772 | 0.985 | 0.921 | 0.492 | < 0.05 | 2.002 | < 0.05 | 1.958 | < 0.05 |
| Anserine | 5.109 | < 0.05 | 6.053 | < 0.05 | 5.900 | < 0.05 | 1.026 | 0.763 | 0.866 | 0.052 |
| Betaine | 0.950 | 0.058 | 1.508 | < 0.05 | 0.245 | < 0.05 | 6.166 | < 0.05 | 3.882 | < 0.05 |
| Creatine | 0.621 | < 0.05 | 0.548 | < 0.05 | 0.864 | 0.079 | 0.634 | < 0.05 | 0.719 | < 0.05 |
| Formate | 0.971 | 0.776 | 0.936 | 0.477 | 0.911 | < 0.05 | 1.027 | 0.757 | 1.066 | 0.512 |
| Fumarate | 1.235 | 0.091 | 1.173 | 0.137 | 1.040 | 0.797 | 1.128 | 0.428 | 1.188 | 0.302 |
| Glutamate | 1.342 | < 0.05 | 1.516 | < 0.05 | 1.376 | < 0.05 | 1.102 | 0.189 | 0.975 | 0.841 |
| Glutamine | 0.892 | 0.518 | 0.980 | 0.872 | 1.025 | 0.633 | 0.956 | 0.724 | 0.870 | 0.439 |
| Glycine | 1.554 | < 0.05 | 2.115 | < 0.05 | 0.501 | < 0.05 | 4.222 | < 0.05 | 3.101 | < 0.05 |
| Guanidoacetate | 0.928 | 0.330 | 0.929 | 0.227 | 0.675 | < 0.05 | 1.376 | < 0.05 | 1.374 | < 0.05 |
| Hypoxanthine | 3.722 | < 0.05 | 4.374 | < 0.05 | 2.646 | < 0.05 | 1.653 | < 0.05 | 1.407 | < 0.05 |
| IMP | 3.505 | < 0.05 | 5.108 | < 0.05 | 3.209 | < 0.05 | 1.592 | < 0.05 | 1.092 | 0.442 |
| Lactate | 3.114 | < 0.05 | 3.819 | < 0.05 | 4.500 | < 0.05 | 0.849 | 0.184 | 0.692 | < 0.05 |
| Leucine | 1.000 | 0.999 | 0.851 | 0.233 | 0.883 | 0.190 | 0.964 | 0.786 | 1.133 | 0.291 |
| Mannose | 1.093 | 0.527 | 0.783 | 0.151 | 0.965 | 0.746 | 0.811 | 0.172 | 1.132 | 0.298 |
| Myo−inositol | 0.903 | 0.361 | 0.964 | 0.795 | 0.530 | < 0.05 | 1.819 | < 0.05 | 1.704 | < 0.05 |
| N,N−dimethylglycine | 0.777 | 0.080 | 1.128 | 0.449 | 0.198 | < 0.05 | 5.685 | < 0.05 | 3.915 | < 0.05 |
| NAD+ | 0.832 | 0.061 | 0.790 | < 0.05 | 0.422 | < 0.05 | 1.873 | < 0.05 | 1.971 | < 0.05 |
| Niacinamide | 1.189 | 0.142 | 1.090 | 0.591 | 1.315 | 0.078 | 0.829 | 0.325 | 0.904 | 0.446 |
| Phenylalanine | 0.211 | < 0.05 | 0.350 | < 0.05 | 0.519 | < 0.05 | 0.674 | < 0.05 | 0.407 | < 0.05 |
| Taurine | 0.291 | < 0.05 | 0.256 | < 0.05 | 1.669 | < 0.05 | 0.153 | < 0.05 | 0.174 | < 0.05 |
| Trimethylamine | 0.554 | < 0.05 | 0.487 | < 0.05 | 0.234 | < 0.05 | 2.078 | < 0.05 | 2.367 | < 0.05 |
| Uracil | 0.583 | < 0.05 | 1.433 | < 0.05 | 0.393 | < 0.05 | 3.646 | < 0.05 | 1.484 | < 0.05 |
| Valine | 1.014 | 0.903 | 1.185 | 0.061 | 1.116 | 0.085 | 1.062 | 0.389 | 0.909 | 0.413 |
| α−glucose | 0.969 | 0.816 | 0.985 | 0.889 | 0.988 | 0.904 | 0.998 | 0.982 | 0.981 | 0.886 |
| β−alanine | 8.489 | < 0.05 | 1.347 | < 0.05 | 2.236 | < 0.05 | 0.602 | < 0.05 | 3.797 | < 0.05 |
| β−glucose | 0.969 | 0.816 | 0.985 | 0.889 | 0.988 | 0.904 | 0.998 | 0.982 | 0.981 | 0.886 |

* Statistically significance was indicated by P < 0.05
